# Electropolymerization of Organic Mixed Ionic-Electronic Conductors: Fundamentals and Applications in Bioelectronics

**DOI:** 10.1021/acs.chemrev.5c00183

**Published:** 2025-12-17

**Authors:** Jennifer Y. Gerasimov, Mary J. Donahue, Dace Gao, Deyu Tu, Simone Fabiano

**Affiliations:** Laboratory of Organic Electronics, Department of Science and Technology, 4566 Linköping University, Norrköping 60174, Sweden

## Abstract

Conducting polymers, particularly those capable of transporting both ionic and electronic charges—commonly referred to as organic mixed ionic-electronic conductors (OMIECs)—have played a transformative role in enabling bidirectional communication between biological systems and electronic circuits. This ability has driven the field of bioelectronics to expand in three distinct directions: biointerfacing, sensing, and neuromorphic computing. Biointerfacing and sensing allow for the extraction of interpretable chemical and electrochemical signals from living organisms, while neuromorphic computing, in addition to efficiently processing complex signals, can translate electronic signals into the frequency domain that the nervous system uses to communicate. In the bioelectronics context, OMIECs have been untethered from previous requirements of high charge mobility, fast switching times, and long-range crystallinity, which makes electropolymerization a more attractive route to fabricate OMIECs on bioelectronic devices. This review examines the fundamental principles, practical aspects, and prominent applications of OMIEC materials fabricated by electropolymerization.

## Graphical abstract

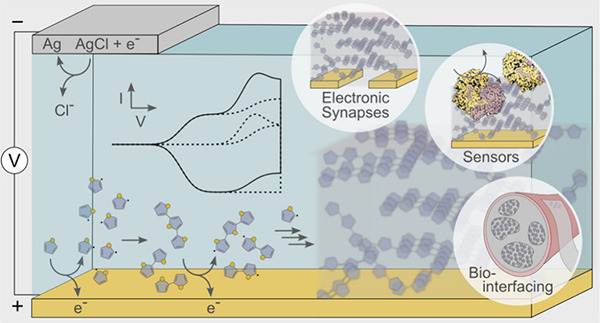

## 1 Introduction

Electrochemical conversion of soluble reactants into insoluble products as a means of modifying conductive surfaces has a long history of practical applications that can be traced back to the early 19th century with the first report of metal electrodeposition. In 1803, Luigi Brugnatelli, a professor and close associate of Alessandro Volta at the University of Pavia, used the voltaic pile, a recently invented device that made sustained current possible, to electrochemically deposit a layer of gold onto silver medals.^1^ This process, using a gold-saturated aqua regia and ammonia electrolyte, demonstrated the potential of electrochemical deposition to create metal coatings and sparked a growing interest in electrodeposition as a means of surface modification in chemistry and materials science.^2^

Although a clear analogy can be drawn between the electrochemical deposition of metals and of conducting polymer films, the two processes are mechanistically very different. Metals are deposited via a simple reduction process, wherein one or more electrons are transferred from an electrode to a metal cation dissolved in the electrolyte, producing a solid material that deposits on the electrode. In contrast, electrochemical polymerization of conducting polymers proceeds through a series of oxidation steps involving multiple charged and neutral intermediates that each have distinct solubilities, diffusion coefficients, electrophoretic mobilities, and electron affinities. While Brugnatelli’s work laid the foundation, it was not until more than half a century later that electropolymerization of polyaniline was first reported^3^ and not until a century and a half later that conducting polymers were recognized as such.^4^

Electropolymerization was the first and initially preferred mode of electrode functionalization with conducting polymers because it allows for highly localized synthesis on select conductive surfaces, facile separation of reactants and products, and precise control of polymer thickness. Notable reviews describing the state-of-the-art in electropolymerized conducting polymers at the time include reviews by Waltman,^5^ Heinze,^6^ and Otero.^7^ However, the approach has faced numerous challenges that have historically limited its widespread adoption. Early work with conducting polymers primarily aimed to create highly ordered conducting films for applications in solar cells, light-emitting diodes, capacitors, and organic transistors, where high charge mobility, fast switching times, and long-range crystallinity are paramount. These characteristics are difficult to achieve consistently through electropolymerization, especially when attempting to control crystallinity, molecular weight distribution, and defect density.^8–10^

The rapid development of organic mixed ionic-electronic conductors (OMIECs)—electrically conducting polymers that can also accommodate the mass transfer of ions throughout the material bulk—has expanded the relevance of conducting polymers to applications that do not necessitate long-range crystallinity, triggering a renewed interest in electropolymerization.^11,12^ The mixed mode of conduction allows ions that penetrate into the OMIEC from the electrolyte to effectively compensate for electronic charge traveling through the conducting polymer, providing for a soft, three-dimensional electrode–electrolyte interface that greatly reduces interfacial impedance. OMIECs have become almost ubiquitous in the fields of biological interfaces and neural implants by reducing the impedance of microscale electrodes and providing improved compatibility with the mechanical properties and ionic signaling pathways of biological systems.^13,14^ Recent advances in the use of water-soluble and solubilized OMIEC precursors have demonstrated that polymerization can also be induced *in vivo* within the water-splitting potential window, reducing the risk of side reactions and broadening the scope of bioelectronic applications.^15–17^ This development has initiated a renewed interest in the field, as more researchers focus on materials that allow for safe, low-voltage polymerization that is compatible with biological environments. Other technologies, like neuromorphic synapses, even derive some benefits from the comparatively low mobility of OMIECs, as it leads to reduced power consumption for a device with equivalent dimensions. Overall, electropolymerization promises to be a disruptive technology in the field of bioelectronics because it offers a simple, versatile strategy toward local device fabrication. Further, it introduces new functions to bioelectronics technologies that cannot be attained by photolithography, like encasing biological structures,^18^ integration with tissue,^16^ embedding enzymes,^19^ and programmable device fabrication.^20^

While the scope of this review is targeted to cover electropolymerized OMIECs rather than materials that are not electroactive throughout the bulk, the distinction between these two categories is somewhat fuzzy. A certain extent of ionic conductivity is shared by virtually all conducting polymers.^21^ When a suitable electrolyte is used, even materials that are notoriously impermeable to ions can reveal mixed conduction.^22^ Other electropolymerized materials form a highly porous structure that facilitates the penetration of ions into the material bulk, which makes it somewhat indistinguishable from ‘true’ OMIECs.

In this review, we aim to provide the reader with an overview of the fundamental and practical knowledge necessary to design, implement and troubleshoot research in the field of electropolymerized OMIECs. We further present a snapshot of the fields in which these materials are advancing bioelectronics technologies, such that the reader is well-equipped to expand on the current state-of-the art. In Section 2, we outline important factors to consider when designing an electropolymerization experiment, including materials selection, the various electropolymerization mechanisms proposed to date, experimental design, morphology control, instrumental configuration, and *in situ* characterization of the electropolymerization process. In Section 3, we review the emerging bioelectronic applications of electropolymerized OMIECs, including biological interfacing to bridge the communication divide between biological systems and electronics, sensors and biosensors for monitoring a variety of analytes from protons to viruses, and neuromorphic synapses for information processing.

## 2 Designing an Electropolymerization Experiment

### 2.1 Materials

#### 2.1.1 Organic Mixed Ionic-Electronic Conductors

The nature of charge transport within OMIECs requires that the electronic charge traveling through a network of polymers or small molecules is compensated by an adjacent ion of opposite charge to maintain charge neutrality within the material. Thus, the process of electrochemical doping, which modulates the conductivity of organic semiconductor materials, comprises both the injection of electrical charge from an electrode into the material and the injection of ions from the electrolyte.^23^ OMIECs are unique within the more general category of organic semiconductors because ions can penetrate into the material bulk, allowing electronic charge to travel throughout the volume of the material rather than exclusively along the interface with an electrolyte or polarized dielectric.^24,25^

To achieve effective charge compensation throughout the material bulk, the components that confer electrical conductivity and ionic conductivity are generally blended or covalently linked to produce a material that provides a continuous paths for both the movement of electronic charge throughout the material and for the movement of ionic charge from the electrolyte into the internal volume of the material (Figure 1).^12^ In an OMIEC material, the transport of electronic charge in the form of holes (p-type materials) or electrons (n-type materials) happens along the delocalized π system of a conjugated polymer chain and between overlapping π systems of neighboring chains within a network. In contrast, ionic charge is transported through the negative volume surrounding the conjugated polymer chains, which is composed of ion-permeable micro- and nanodomains within the material. The transport mechanisms of both electronic and ionic charge in OMIECs have been clearly summarized by Kim et al.^13^ While n-type OMIECs are the focus of a highly active and fascinating field of research, they are challenging to synthesize by oxidative electrochemical polymerization. This is because they are depleted of their primary charge carrier and form an insulating film upon oxidation. For this reason, the discussion will be limited to p-type OMIECs in this review.

**Figure 1:**
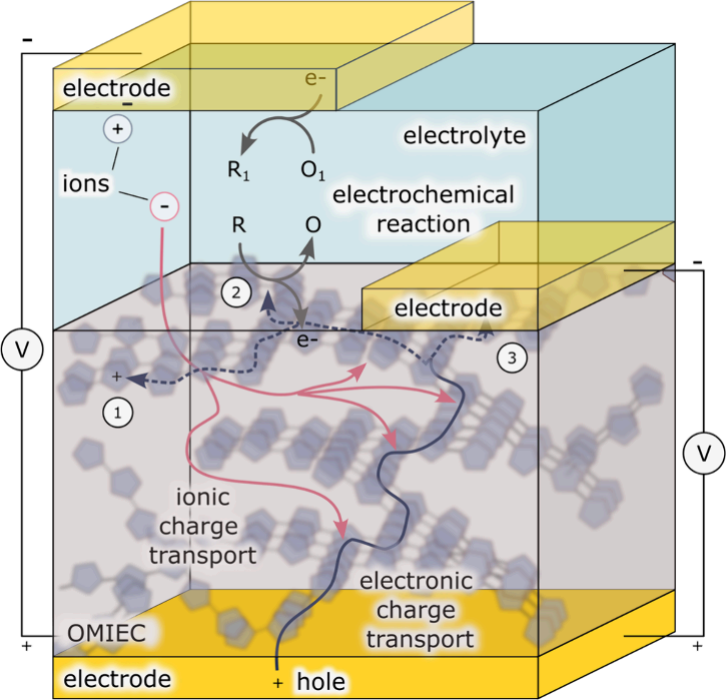
Charge transport pathways and charge transfer processes within an OMIEC material. OMIEC materials are conducive to the transport of both electronic charge (blue arrows) and ionic charge (pink arrows). In the illustrative example, the electrode in contact with the OMIEC acts as the anode while the electrode that contacts the electrolyte acts as the cathode. Once electronic charge is injected into an OMIEC material deposited at an electrode (solid blue line), there are three potential pathways that it could take (dashed blue arrow). The preferred pathway is determined by device structure and the magnitude of the applied voltage. If the OMIEC is not in direct contact with the cathode, the charge can either be stored in the OMIEC material (1) or, if the applied potential is sufficient, transferred to a redox species in the electrolyte (2). Otherwise, if the cathode is in direct contact with the OMIEC, charge will flow through the OMIEC between the two electrodes (3).

Depending on the experimental conditions, one or more of the following processes can take place when a potential is applied to an OMIEC material deposited at an electrode. In the simplest case, when the electrode of opposite polarity is in contact only with the electrolyte and the potential applied between the electrodes is insufficient to drive electrochemical reactions in solution, positive charge accumulates within the conjugated polymer network and is compensated by anions from the electrolyte. Simultaneously, an equal amount of charge accumulates at the cathode to compensate for the unpaired cations in the electrolyte. Alternatively, at a nonpolarizable cathode like Ag/AgCl, Cl^–^ anions are generated through a Faradaic process. In both of these cases, the OMIEC behaves as a three-dimensional electrical double layer (EDL) capacitor.^26–28^ In the charged, conductive state, the electrode–electrolyte interface of an OMIEC-coated electrode exhibits a much lower impedance than metal electrodes. The dramatic reduction in impedance renders these interfaces highly sensitive to ionic fluctuations in the electrolyte, which confers many of the properties required to construct effective biological interfaces (Section 3.1).

When the potential applied to the OMIEC exceeds the oxidation potential of a dissolved species, the Faradaic transfer of electronic charge occurs at the OMIEC-electrolyte interface. In the example given in Figure 1, an electron is abstracted from a species dissolved in solution (R) and shuttled to the electrode in contact with the OMIEC, which leads to the conversion of R to its oxidized form (O) in solution. At the opposing electrode, an electron is transferred either to O or to a different redox species entirely to maintain the charge neutrality of the electrolyte. Direct electrochemistry on dissolved species at the surface or with the internal volume of OMIECs enables many of the sensing applications discussed in Section 3.2 of this review.

In the third experimental configuration, when a second electrode is in contact directly with the OMIEC materials, charge can travel through the material between the two electrodes. In this configuration, the doping level within the OMIEC channel between the two electrodes can be independently controlled by a third electrode that is in contact with the electrolyte. This device configuration, referred to as the organic electrochemical transistor (OECT), has developed into something of a bioelectronics workhorse, as it is vital to the amplification of biological ionic currents, biosensing, and neuromorphic applications.^29–32^

#### 2.1.2 Key Materials Properties

The performance of OMIEC materials can only be evaluated in the context of a given application. In optimizing material properties toward an application, a set of key material parameters are maximized, minimized, or balanced, depending on the applicational requirements. In this section, we provide a brief overview of the parameters important for material optimization in the service of each of the applications outlined in this review—biological interfacing, sensing and biosensing, and neuromorphic synapses. It should be noted that some of the parameters listed are not truly independent of each other. For example, due to the mixed mode of conduction, electrical conductivity is inextricably linked to ionic conductivity. Likewise, response time is determined by the mobility of ionic or electronic charge carriers, which are also integral to ionic and electrical conductivity. Nevertheless, as seen in Table 1, which summarizes the importance of key OMIEC parameters for each of the applications outlined in this review, parameters that share contributing factors are not always correlated in importance.

**Table 1:** Summary of the Directionality of Parameter Optimization for Each of the Applications Reviewed Herein^a^

Application		Parameter Optimization					
	Electrical conductivity	Ionic conductivity	Response time	Elasticity/flexibility	Aqueous monomer solubility	Oxidation potential	Monomer toxicity
**Biological interface**							
Stimulation	+++	+++	- - -	+++	0	-	-
Recording	+++	++	- -	+++	0	-	-
*In vivo* electrode formation	+++	+++	- -	++	+++	- - -	- - -
**Sensors**							
Enzymatic	+	++	-	0	++	- -	-
Molecular imprinting	+	++	-	0	+/0	0	-
Direct redox	++	+	-	0	0	-	-
Intrinsic polymer properties	+	+++	-	0	0	-	-
**Neuromorphic synapse**	-/0	*++*	-	0	0	-	-

a The symbols ‘+’ and ‘–‘ indicate whether the application calls for the parameter to be maximized or minimized, while the number of symbols indicates the importance of parameter optimization for adequate performance. The symbol ‘0’ indicates that the parameter is not vital to performance for a given application.

##### 2.1.2.1 Electrical Conductivity

The electrical conductivity of an OMIEC, or the ease with which electrical charge can pass through the material, is measured as the conductance (inverse of the resistance) across the material normalized by the material dimensions (multiplied by the length, divided by the cross-sectional area). Assuming that charge compensation is not a limiting factor, electrical conductivity in an OMIEC material is proportional to the concentration and mobility of the charge carriers within the material. While the mobility depends on the order and interconnectivity of the electrically conducting elements within the OMIEC, the concentration of charge carriers depends on the doping level and the surface area of the interface between the electrically conducting elements. The mechanisms of electronic charge transfer within OMIEC materials have been thoroughly reviewed by Paulsen et al.^33^

Of all the applications reviewed herein, maximizing the electrical conductivity is the most essential to the field of biointerfacing—an active area of research that is concerned with investigating the interface between electronic systems and electrogenic cells.^34–37^ One of the challenges in this field is that the process of scaling down the area of a recording electrode to achieve single-cell spatial resolution is accompanied by a proportional increase in the interfacial impedance, but not a reduction in the noise. When the signal and the noise are with the same order of magnitude, it is difficult to distinguish the action potential generated by the electrogenic cell under investigation and environmental electromagnetic fluctuations. The three-dimensional nature of the OMIEC EDL greatly reduces the impedance of the interface, making electropolymerized OMIECs quite useful to downscale electrode dimensions without sacrificing the signal-to-noise ratio. High electrical conductivity of the interface material enhances the sensitivity of the recording electrode to the electrical signal originating at electrogenic cells, leading to an improved signal-to-noise ratio. The reverse communication mode, or stimulation, involves applying a constant current to an electrogenic cell through an electrode to produce an action potential. Stimulation also benefits from high electrical conductivity of OMIEC interfaces because ohmic losses across the interface lead to higher voltages applied to deliver the same current, which results in unwanted side reactions and tissue damage.

In contrast to biointerfacing, electrical conductivity is a metric that should be balanced, rather than maximized, when constructing neuromorphic synapses. Electronic synapses are constructed by electropolymerizing an OMIEC material between unconnected metal electrodes. In this way, the conductance between the electrodes can be easily modulated and is used to represent the ‘synaptic weight’ of the connection between electronic neurons in larger neuromorphic circuits. Overall, highly conductive synapses contribute to high power consumption in neuromorphic processors. This drawback can be mitigated by operating at lower voltages or scaling down the dimensions of the synapse. This may not always be feasible, however, because operating the voltage may be defined by other components in the processor, like the neurons. Likewise, miniaturization, when possible, is likely to affect the linearity and dynamic range of the resulting synapse. The lower limit of the electrical conductivity is set by the level below which different states are no longer distinguishable due to instrumental sensitivity limits or noise.

##### 2.1.2.2 Ionic Conductivity

The ionic conductivity, or the ease with which ions are able to pass through a material, is analogous to electronic conductivity in that it is proportional to the mobility and concentration of ionic charge carriers. Ionic charge carriers, however, can vary in size, valency, and solvation. It follows that the overall ionic conductivity is the sum of the partial conductivities of each ion in the electrolyte. In electropolymerized materials, the ionic conductivity depends on the structure, density, and interconnectivity of the ion-conducting pathways as well as the identity of the ion. As with electronic charge transfer, the mechanism of ionic charge transfer within OMIECs has been thoroughly reviewed by Paulsen et al.^33^

Ionic conductivity is important in designing biological interfaces because ionic currents are the language of the cell and good ionic conductivity, as well as a high interfacial area between ionic and electronic conductors, make for more efficient transduction of the ionic current into an electronic current. In sensing applications, high ionic conductivity has the largest benefit for ion-specific and pH sensors, which require the penetration of ions into the material volume. Neuromorphic synapses also benefit from high ionic conductivity because the mixed mode of conduction allows for the conductance of the synapse to scale with the volume of the conductive channel between two electrodes, rather than the surface area.

##### 2.1.2.3 Response Time

The response time is not exactly a material property, but a somewhat convoluted metric of material and device properties that reflects how quickly a device can switch between different doping levels. Overall, the response time of a device depends on device dimensions^38^ and configuration^39^ as well as the mobility of the limiting charge carrier (ion or electron).^40^

Each application is associated with a unique characteristic time scale at which events happen. In order for an electrode, sensor, or device to be well-suited for its intended application, its maximum response time should be at least 5–10 times shorter than the characteristic time scale of that application. At the faster end of the spectrum, the characteristic time scales are defined the action potential in bioelectronic recordings (ms-range for a neuron) and the required phase length for stimulating pulses at neural interfaces (tens of μs for cochlear implants^41^). For sensors, the response time is generally limited by other factors, like enzyme kinetics, interaction with the analyte, or electron transfer. In neuromorphic systems, the response time of the synapses should be shorter than the spiking of the neurons. However, longer response times can also be used to introduce interesting behaviors like short-term plasticity^42^ and spike timing-dependent plasticity^43^ that may benefit computation.

##### 2.1.2.4 Softness, Elasticity, Flexibility

The softness, elasticity, and flexibility of a material are related, but not equivalent properties describing the ease with which a material can be deformed by and withstand compression, stretching, and bending, respectively.^44^ All of these properties are important for interfacing with living systems, which are soft and dynamic. When interfaced with cells and tissue, sensors and neuromorphic circuits also benefit from these properties. The mechanical properties of OMIEC materials can be modulated by blending with additives like plasticizers, cross-linkers, and hydrogels.^44^

##### 2.1.2.5 Aqueous Monomer Solubility

The electropolymerization of conducting polymers, with the exception of polyaniline (PANI), has historically been carried out predominantly in organic solvents due to the poor aqueous solubility of conjugated molecules. However, the solubility of the monomer in aqueous solutions is essential for 1) *in vivo* electropolymerization, due to the water-based nature of living systems, and 2) applications where electropolymerization is conducted in the presence of biological molecules, like DNA or enzymes, that would either be insoluble or unstable in nonaqueous solvents. Other applications, like sensors, neuromorphic devices, and biointerfaces, where a device is prepared outside of a biological or a biocompatible environment, derive no direct benefits from aqueous monomer solubility.

##### 2.1.2.6 Oxidation Potential

The oxidation potential of the monomer is the potential above which the electropolymerization reaction proceeds at the electrode whereas the oxidation potential of the polymer is the potential at which the OMIEC material transitions between the insulating and conducting state. The oxidation potentials of both the monomer and the polymer—distinct but correlated values—are important parameters to consider for every application. The compatibility between these values and the application is determined by whether the monomer oxidation potential 1) falls within the electrochemical window of the solvent, 2) is low enough to not induce side-reaction with other electroactive or electrolabile species in the electropolymerization mixture, and 3) is high enough to not occur spontaneously in the presence of other chemical oxidants present in the reaction mixture. In optimizing this parameter, each application should be evaluated on a case-by-case basis, but a general trend in our evaluation is that this optimization becomes more important in complex electropolymerization mixtures. In the case of the neuromorphic synapse, minimizing the monomer oxidation potential leads to a reduction in the power consumption for programming a synaptic weight. The oxidation potential of the polymer is important because it defines the potential boundary between the conducting and insulating states, which affects the performance and power consumption of biological interfaces^45^ and OECT-based sensors.^46,47^

##### 2.1.2.7 Toxicity

Toxicity, or how harmful a substance is when it is allowed to interact with a biological organism, is generally not a favorable material property for any application because electropolymerization itself is often carried out by biological organisms. However, the minimization of toxicity arising from OMIEC materials, solvents, and the electropolymerization process is especially important for *in vivo* applications.

##### 2.1.2.8 Stability

Outside of some niche applications, like transient electronic devices,^48^ stability should be maximized for all applications, and was therefore not included in Table 1. There is no widely accepted standard metric for stability, but it is often reported as time until failure or as the extent of conductance loss within a given period of time. OMIEC-based materials and devices are subject to various modes of failure, and their relevance depends on the specific environment and operational parameters established by the application.

The most common modes of failure are not specific to electropolymerized devices, but are relevant in tailoring materials to the applications outlined here. Mechanical breakdown of electropolymerized films commonly occurs due to stresses from dynamic environments or repeated swelling and contraction as ions move in and out of the film. A film’s resistance to mechanical breakdown depends on the internal microstructure, which can be modified by the electropolymerization conditions, as described by Wang et al.^49^ and in Section 2.3.6. Delamination occurs when weak substrate adhesion, coupled with mechanical stresses, causes the film to detach. Mechanical and chemical methods of improving adhesion between the electropolymerized film and the substrate are reviewed in Section 2.3.2. Electrochemical breakdown of the material occurs when the conjugated backbone is irreversibly oxidized in response to an applied potential^50^ and when reactive oxygen species are electrochemically generated during operation.^51,52^ Electrochemical breakdown of the material can be mitigated by limiting the operating range of the electrode or device or, alternatively, by coating the OMIEC with layer that is impermeable to oxygen.^52^ Thermal degradation mechanisms vary depending on the material and the temperature to which the material is exposed.^53,54^ Thermal stability in electropolymerized conducting polymers has been improved by backbone engineering^55^ and blending with clay composites.^56^ Lastly, OMIEC-based electrodes and devices used in vivo are prone to inducing an inflammatory response, which leads to their immunological degradation.^57^ Zwitterionic conducting polymers have been shown to resist degradation induced by the immune response.^58^

#### 2.1.3 Survey of Materials

Among organic conductors suitable for electropolymerization, polythiophene and its derivatives have garnered considerable attention due to their unique properties (Figure 2). The simplicity of the thiophene ring structure, coupled with its ease of modification, renders it a versatile material for various applications. Unsubstituted thiophene has a high oxidation potential of 2.05 V (vs Ag/AgCl).^59^ Alkyl-substituted polythiophenes (**1**), such as the renowned P3HT, feature alkyl chains that reduce the oxidation potential of the monomer (1.35 V vs Ag/AgNO_3_), while enhancing the solubility, processability, and flexibility of the electropolymerized films.^60^ Substitution at the 3-position of the thiophene has limited impact on polymer conjugation and has thus been used to produce monomers with a broad variety of functional groups, including acrylate,^61^ p-nitrophenyl,^62^ and oligonucleotide^63^ groups. 3,4-Ethylenedioxythiophene (EDOT, **2**) is a thiophene derivative characterized by an electron-donating ethylenedioxy bridge. Such substitution effectively reduces the oxidation potential (∼1.4 V vs Ag/AgCl),^64^ resulting in improved conductivity, structural/morphological integrity, and more stable oxidation state in poly-EDOT (PEDOT) and its derivatives. To date, structural modifications of the EDOT core have been achieved to optimize electropolymerization parameters, primarily by reducing the oxidation potential,^65,66^ enhancing the polymer’s electrochemical and mechanical durability,^67^ modulating its micromorphology,^68^ and improving the electrochemical properties^69^ of the resulting conductive polymer, thereby imparting novel functionalities at the device level.

**Figure 2:**
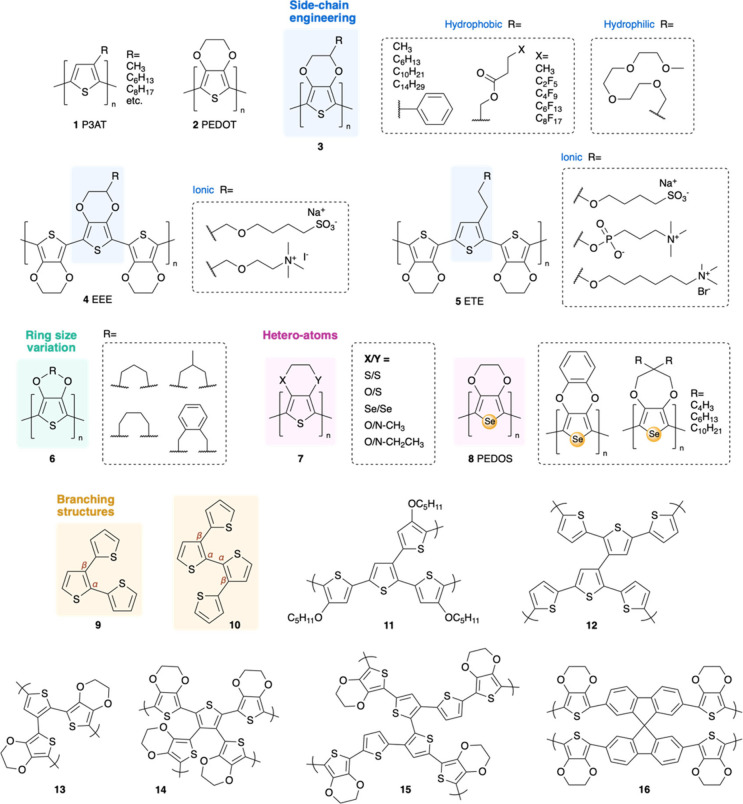
Structures of electropolymerizable polythiophene derivatives.

EDOT demonstrates remarkable versatility in grafting side chains to the ethylenedioxy bridge (**3**). Various derivatives of EDOT, featuring tailored pendant chains—including hydrophobic, hydrophilic, and ionic groups—have been developed to refine the properties of the resulting polymers.^70^ The incorporation of hydrophobic alkyl side chains^71,72^ enhances the solubility of EDOT derivatives in organic solvents, thereby facilitating the electropolymerization process and allowing for improved control over film growth. When fluorinated side chains are introduced to EDOT, the electropolymerized films exhibit a fiber-like surface morphology, achieving superhydrophobic characteristics (with water contact angles exceeding 150°) and demonstrating exceptional antiwetting properties.^73^ Conversely, the integration of hydrophilic groups,^74^ particularly ethylene glycol side chains,^75^ significantly enhances solubility and compatibility in aqueous environments, resulting in smooth, well-adhered films that are crucial for applications in such settings. The hydrophilicity conferred by glycolated side chains also promotes efficient ion transport, a vital attribute for devices such as OECTs and biochemical sensors. Furthermore, these chains improve biocompatibility, rendering glycolated PEDOT derivatives suitable for various biological applications, such as *in vitro* extracellular recordings of neuronal activities^75^ under physiological conditions.

Ionic side chains have similarly been incorporated into EDOT derivatives, facilitating electropolymerization in aqueous, physiological environments. ETE trimers (**4**)^20,42,76^ have demonstrated notable success *in vivo*, allowing polymerization within living tissues and organs^15^ to establish bioelectronic interfaces. The trimeric structure of ETE is particularly advantageous: it features a central thiophene group that serves as an anchoring site for side chain incorporation, while the two outer EDOT molecules possess lower oxidation potentials compared to thiophene monomers, thus rendering the trimer highly reactive and conducive to polymerization (0.63 V vs Ag/AgCl for ETE-S,^65^ in contrast to 1.4 V for EDOT). Recently synthesized EEE trimers (**5**), which replace the central thiophene with EDOT, exhibit further reduction in polymerization potential (0.47 V vs Ag/AgCl for EEE-S)^65^ and enhanced electrical conductivity due to improved planarity. The polarity of the ionic side chains plays a crucial role in both the onset potential of electropolymerization and the electrochemical properties of the derived conductive polymers. Anionic moieties, such as sulfonate groups, facilitate self-doping of the thiophene backbone, effectively enhancing intrinsic conductivity without the need for external dopants. In contrast, cationic moieties, such as ammonium groups, do not confer a self-doping effect and may shift the electropolymerization of ETE/EEE trimers toward higher potentials,^76^ likely due to lower solubility and the electrostatic repulsion between the positively charged side chains and the oxidizing electrode. Although these cationic variants may be less reactive and conductive, their slower kinetics offers improved control over film formation,^65^ which could be advantageous for forming bioelectronic interfaces.

Varying the size of the alkylenedioxy ring in EDOT (**6**) presents an alternative approach for generating PEDOT derivatives with distinct rigidity and electrochemical properties.^72^ The introduction of a propylene group^66^ in place of the ethylene moiety imparts additional flexibility, resulting in a reduced oxidation potential of approximately 0.9 V (vs Ag/AgCl), enhanced intrinsic conductivity in organic solvents (even in the absence of dopants), and an improved color contrast in electrochromic applications. Conversely, substituting the ethylenedioxy unit with a benzodioxane group^77^ serves to expand the π-conjugation and increase the rigidity of the polymer chains. This modification promotes a more planar structure, which can lead to greater stability and a higher degree of conjugation during electropolymerization, ultimately enhancing the electrochemical stability of the resultant polymer. However, the fused-ring xylene group also increases monomer oxidation energy, requiring more positive potential (1.7 V vs Ag/AgCl) than PEDOT to initiate electropolymerization.^77^

Additionally, replacing the oxygen atoms in the ethylenedioxy bridge of EDOT with other heteroatoms, such as sulfur or nitrogen (**7**), results in a slight reduction of the monomer’s oxidation potential, facilitating easier electropolymerization. Sulfur, being less electronegative than oxygen, enhances electron donation to the thiophene ring and stabilizes the radical cation intermediates generated during polymerization. For instance, the disulfur analogue of EDOT (i.e., 3,4-ethylenedithiothiophene, EDTT) shows an oxidation potential 0.18 V lower than EDOT^78^ ascribing to the stronger electron-donating aptitude of alkylsulfanyl groups.^79^ However, the incorporation of alkylsulfanyl groups may elevate the oxidation potential of the resulting polymer and limit its effective conjugation length due to increased steric hindrance. Nitrogen, while more electronegative than sulfur yet less so than oxygen, introduces a degree of electron-donating character and adds potential sites for hydrogen bonding or protonation.^80^ This property is particularly advantageous for applications in supercapacitors and energy storage devices. Furthermore, nitrogen with alkyl pendant groups can induce steric effects that modify polymer packing,^81^ potentially impacting film morphology. Substituting sulfur with selenium in the thiophene ring of EDOT yields 3,4-ethylenedioxyselenophene (EDOS) and its polymer form (**8** PEDOS),^82,83^ a selenophene-based conjugated polymer with lower electronegativity, lower aromaticity, and more quinoid preference over PEDOT.^83^ DFT calculations suggest that the band gap of polyselenophenes (1.85 eV) is lower than that of polythiophene (2.03 eV), which favors electron removal (oxidation) and reduces the electropolymerization potential.^84,85^ These factors in synergy allow EDOS to electropolymerize at a lower voltage (∼1.22 V vs Ag/AgCl)^86^ compared to EDOT. Furthermore, the larger atomic radius and greater polarizability of selenium atoms, relative to sulfur, contribute to increased π-conjugation, leading to heightened conductivity and a red-shifted, broader absorption spectrum.^82^ These characteristics render PEDOS particularly suitable for optoelectronic applications, such as OPVs and photodetectors. Additionally, the greater atomic size of selenium in PEDOS facilitates a more flexible polymer backbone compared to that of PEDOT, thereby enhancing the mechanical resilience of PEDOS films and making them well-suited for flexible or stretchable electronic applications. However, selenium’s lower bond dissociation energy may compromise the thermal and oxidative stability of PEDOS relative to PEDOT, rendering it more susceptible to degradation.^87^ Consequently, while PEDOS presents promising advancements in electronic and optical performance, it may necessitate stabilization for applications in more demanding environments.

Apart from linear thiophene derivatives, the electropolymerization of terthiophene (**9**) or quaterthiophene (**10**) cores facilitates the formation of branched polythiophenes (**11, 12**) characterized by three-dimensional structures and functional advantages.^88^ Larger oligomers not only promote more efficient polymerization at reduced potentials but also enhance conjugation along each branch of the resultant polymer network. By manipulating branching density and incorporating functional groups, these structures can yield dendrimeric networks that exhibit improved π-conjugation and ion transportation, meanwhile potentially lowering the bandgap and broadening light absorption across a wider spectral range, making them suitable for electrochemical and electro-optical applications. Moreover, higher-order thiophene oligomers provide a greater degree of morphological control during electropolymerization. For instance, organic conductive films exhibiting long-range order and a nanotextured morphology^89^ have been achieved by introducing a rigid spirobifluorene core (**14**) into a quaterthiophene oligomer prior to electropolymerization. Additionally, researchers have utilized thiophene as the core while incorporating more reactive EDOT as the end group^90^ (**13–16**) to further lower oxidation potentials and attain denser, more uniform thin films through the electropolymerization process.

In addition to polythiophene derivatives, classical organic conductors such as polypyrrole (PPy, **17**)^91^ and PANI (**18**) are also well-suited for the electropolymerization technique (Figure 3). Pyrrole exhibits an oxidation potential in aqueous solutions around 0.7 V (vs SCE),^92^ while aniline’s oxidation potential hovers around 0.8 V (vs Ag/AgCl),^93^ influenced by the pH of the solution, with lower potentials observed in acidic environments. However, the prominence of PPy and PANI in electropolymerization studies has been lower than polythiophenes, particularly PEDOT derivatives, for several reasons. First, the conductivity of PPy and PANI in their doped states can be more than an order of magnitude lower than that of doped PEDOT,^94^ such as the commercially prevalent PEDOT:PSS. Additionally, PPy films are often more brittle than the PEDOT counterparts, rendering them less suitable for flexible electronic applications. Furthermore, PPy lacks the extensive tunability in optical properties that polythiophene derivatives provide, which limits its potential in optoelectronic applications. Its primary advantages lie in its straightforward synthesis and robust electrochemical stability. PANI’s conductivity and stability are notably sensitive to environmental conditions, particularly pH and humidity. PANI films also exhibit reduced flexibility and tend to degrade more rapidly in highly alkaline environments. Moreover, both PPy and PANI have fewer derivatives available compared to polythiophene, which constrains opportunities for further optimization through structural design.

**Figure 3:**
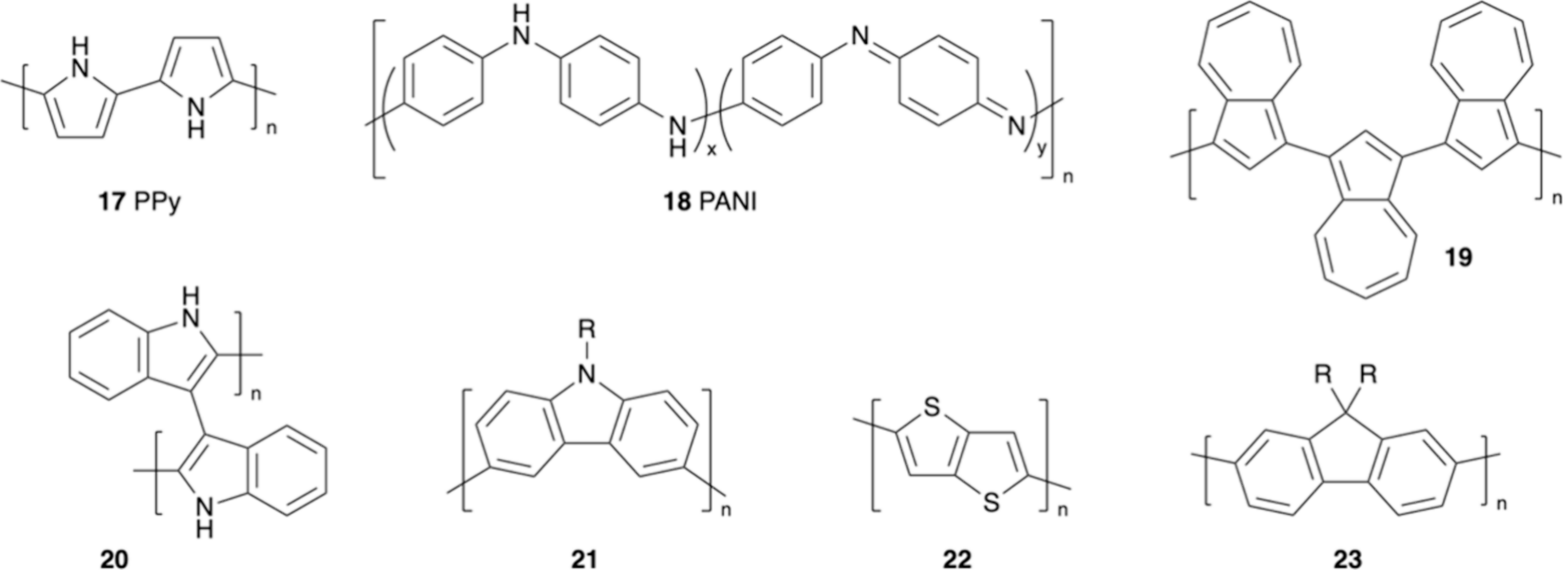
Structures of PPy, PANI, and fused-ring conjugated polymers that are electropolymerizable.

Fused-ring organic conjugated polymers, including polyazulene, polyindole, polycarbazole, polythieno­[3,2-*b*]­thiophene, and polyfluorene, are also electropolymerizable while exhibiting exceptional electronic properties stemming from their extensive π-conjugation and robust ring structures. Polyazulene (**19**),^95^ characterized by its nonbenzenoid fused five- and seven-membered rings, demonstrates unique redox characteristics and remarkable oxidation stability,^96^ rendering it an ideal candidate for applications in supercapacitors, sensors, and electrochromic devices. Structurally similar, polyindole (**20**)^97^ possesses fused five-membered rings and showcases lower oxidation potentials alongside moderate flexibility, which enhances its performance in electrochemical capacitors and biosensors. Transitioning to six-membered rings, polycarbazole (**21**)^98^ integrates a nitrogen heteroatom within a fused tricyclic system, offering superior hole mobility, stability, and optical properties. These features are particularly beneficial for electrochromic and energy storage applications.^99^ Polythieno­[3,2-*b*]­thiophene (**22**),^100–102^ a polymer based on fused thiophene rings, exhibits high planarity and thermal stability attributable to its electron-rich sulfur atoms and conjugated architecture, making it a stable material suitable for organic field-effect transistors (OFETs) and OPVs.^103^ Lastly, polyfluorene (**23**),^104^ with its fully fused conjugated hydrocarbon backbone, is noted for its high photoluminescence efficiency and blue light-emitting properties,^105,106^ positioning it as a crucial material for display technologies. Collectively, these fused-ring polymers significantly broaden the spectrum of electropolymerizable organic electronics, with each structure contributing distinct redox behavior, stability, or electrochemical characteristics that are well-suited for bioelectronic and neuromorphic technologies.

### 2.2 Electropolymerization Mechanism

#### 2.2.1 Methods of Studying the Mechanism

The view that radical cations play a key role in the electrochemical polymerization of organic materials has been widely accepted in the literature for many decades,^5^ predating even the modern understanding of conducting polymers.^107–109^ However, the high reactivity of these species presents a challenge in the direct experimental observation of reaction intermediates. Mechanisms are therefore often extrapolated from observations of the polymer structure, reaction kinetics, the influence of reaction conditions, and the effects of the reaction on temperature and pH. The lifetimes of short-lived intermediates can be assessed electrochemically by cyclic voltammetry implementing a fast scan rate, which can be used to observe the reduction of the radical cations before they are able to react.^110,111^ Mass spectrometry has also been used to detect reaction intermediates.^112^ The presence of unpaired electrons in the reaction intermediates makes electron paramagnetic resonance (EPR) spectroscopy one of the most direct methods of assessing the spin density distribution of monomer and oligomer radicals.^113–117^ However, difficulties in measuring anodically generated radicals arise due to the highly localized nature of the electropolymerization reaction at the electrode and the rapid reaction kinetics of monomer radicals. The few approaches available to facilitate the observation of electrogenerated radicals by EPR include the use of flow cells,^118^ radical-stabilizing solvents,^119,120^ and derivatives with blocked reactive sites.^120–122^ Spin density, as it relates to the electropolymerization mechanism, has also historically been calculated by semiempirical^123–126^ methods and is now more commonly assessed by density functional theory (DFT) studies^127–132^ as well as other theoretical methods.^133^

Given the challenges of studying electrogenerated radicals, some find it worthwhile to evaluate the mechanistic insights provided by alternative methods of radical generation. Chemical, radiolytic, and photolytic^134^ polymerization proceed by a similar mechanism, with the exception that cation radicals are generated uniformly throughout the solution by dissolved species with a fixed redox potential rather than only at the electrode surface. These methods are capable of producing monomer radicals at low concentrations, potentially at low temperatures, and in large volumes, increasing both the radical lifetime and the sensitivity of the measurement. Chemical, photolytic, and radiolytic techniques are often coupled to the evaluation of radical species by EPR^114,135,136^ and UV–vis^137^ spectroscopies. Some caution should be exercised, however, when extrapolating mechanistic details to electropolymerization from alternative polymerization methods due to the vast differences in the experimental conditions. For example, while a chain reaction mechanism was proposed based on observations of the radiolytic oxidation of 3-octylthiophene,^138^ the authors claim that it is not likely to apply to electrochemical electropolymerization due to the high local concentrations of oxidized monomers generated at the anode compared to radiolysis.

#### 2.2.2 Dominant Mechanisms

Electrochemical formation of an OMIEC film is controlled by a number of processes, including 1) interfacial electron transfer at the electrode, 2) chemical reactions among the electrochemically generated soluble intermediates, 3) mass transport through electrically driven migration of charged species and diffusion of uncharged species at the electrode surface, as well as 4) the precipitation and accumulation of insoluble material at the electrode.^139^ When the electropolymerization mechanism is discussed in the literature, it is in reference either to the electrochemical and chemical reactions or to the physical process of nucleation and growth. In this section, we discuss the (electro)­chemical reaction mechanism while mass transport and growth are discussed in more detail in Section 2.3.6.

The mechanism for the oxidative electropolymerization of conducting polymers that is generally accepted in the literature, expertly reviewed by Heinze et al.,^6^ proceeds by radical–radical coupling. This mechanism has been proposed for two major classes of materials: 1) heterocyclic aromatic five-membered rings (most commonly thiophene and pyrrole) and 2) substituted benzene (most commonly aniline). For both material classes, electrode-mediated polymer film formation proceeds in three stages—oxidation, coupling, and propagation.^130^ The process is initiated by monomer oxidation, whereupon a cationic monomer radical is generated through the Faradaic transfer of one electron to the anode (Figure 4). The next stage of the reaction (coupling) occurs when two monomer radical cations recombine in solution to form a covalent bond. The dimerization of these radical cations is known to be extremely fast, with rate constants on the order of 3 × 10^9^ M^–1^ s^–1^.^140,141^ The electrode does not play a direct role in the coupling, but still affects this process by exerting an electromotive force on charged reactants and intermediates.^142^ In the absence of a suitable proton acceptor, the rate of polymer growth is limited by the subsequent proton-elimination step, which governs the conversion of the coupled dication into a neutral molecule.^6^

**Figure 4:**
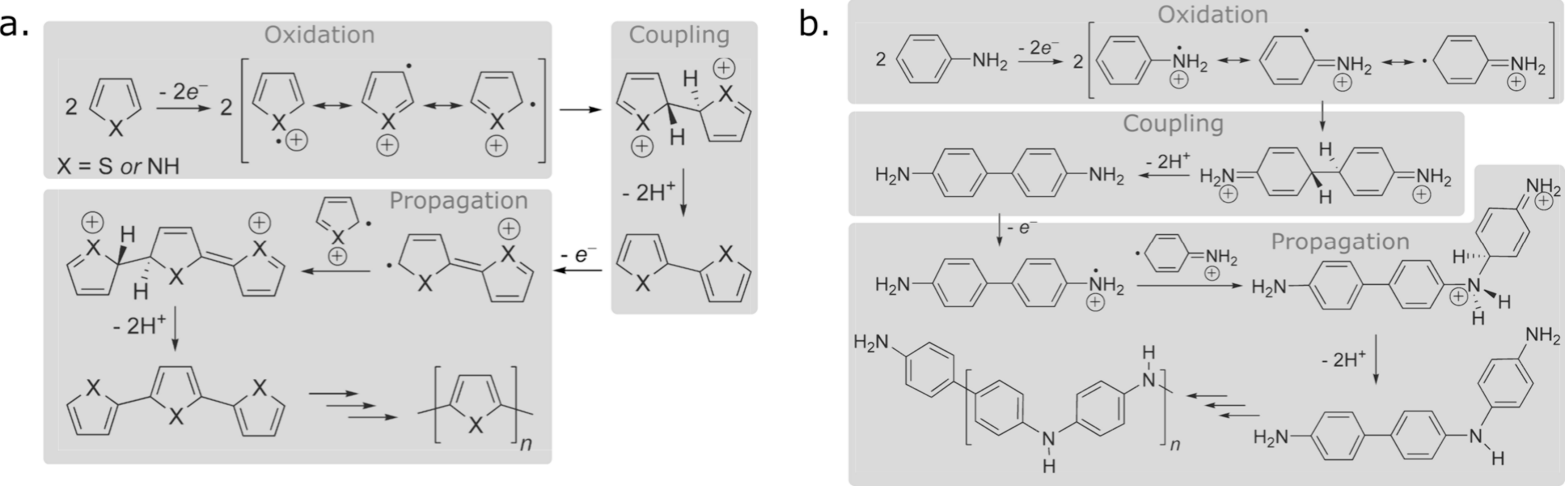
Electropolymerization mechanism for a) polythiophene (X = S) and polypyrrole (X = NH), as well as b) PANI. Adapted from reference 130. Copyright American Chemical Society, 2010.

During propagation, the oxidation-coupling cycle repeats with the electroactive species (neutral monomers, dimers, trimers, etc.) available at the electrode. Several theories have been proposed regarding the n-distribution of the oligomer intermediates during propagation. Based on the observation that the reaction rate constant of the 3-methoxythiophene monomer radical far exceeds that of the dimer radical,^143^ it was postulated that monomers react predominantly with monomers while dimers react predominantly with dimers to form tetramers.^6,144^ However, it is likely that the oligomer distribution is subject to experimental conditions, as the reaction rate constants of pyrrole monomers, dimers, and trimers do not differ dramatically.^141^ Furthermore, trimer radicals have been directly observed by UV–vis and mass spectrometry during the electropolymerization of thiophene,^139,145,146^ functionalized EDOT,^147^ terthiophene,^148^ and aniline.^149^ As the conjugated oligomers formed at the surface increase in mass through monomer addition and cross-coupling among the oligomers, they precipitate and deposit directly onto the electrode, gradually forming a conducting film.^150^

The main distinction between the mechanisms of polythiophene and PANI electropolymerization lies in the number of resonant forms of the monomer radical cation that can successfully combine to form a bond. The thiophene radical, acting here as a representative of the five-membered conjugated heterocycle class, is most reactive at the α-position.^151^ In the case of pyrrole, however, β-coupling becomes increasingly evident at longer chain lengths.^91^ Aniline, however, exhibits two modes of coupling: head-to-tail and tail-to-tail coupling. While head-to-head coupling is possible, it undergoes benzidine rearrangement^152^ to produce the tail-to-tail variant. According to some reports, the tail-to-tail coupling mode dominates at high concentrations of monomer radical,^130,153^ but since only headgroups are available for coupling from a tail-to-tail dimer, the predominant coupling mode of the polymer is head-to-tail. At some conditions, however, it has been reported that no evidence of tail-to-tail coupling was observed.^154^

While the coupling of two radical cations is the most commonly cited initiation mechanism for electropolymerization of conducting polymers, Wei et al. proposed an alternative mechanism based on the observation that the addition of 0.1% dithiophene or trithiophene, which polymerize at lower applied potentials, can dramatically increase the rate and reduce the onset potential of thiophene electropolymerization.^151,155^ A similar effect was reported for aniline, wherein the addition of short oligoaniline additives was shown to reduce the monomer oxidation potential^156^ and increase the polymerization rate by a factor of 10.^157^ This set of observations strongly suggests that oligomeric radical cations are able to couple to neutral thiophene monomers (Figure 5). In this mechanism, monomer oxidation constitutes the first rate-limiting step of the reaction.^151^ Once the oligomers with two or more units are present in solution, their radical cations participate in an electrophilic aromatic substitution reaction with neutral thiophene monomers, followed by oxidation and deprotonation of the resulting oligomer with n+1 subunits. Since its proposal, the mechanism has been refined to account for the instability of the radical on the β-carbon.^158^ Studies of the oxidation of pyrrole by FeCl_3_ ^158^ and ammonium persulfate^159^ also support a departure from strict first-order kinetics, suggesting that oligomers play a catalytic role in the reaction. This is consistent with observations that oligomers with extended π-conjugation can more easily accommodate a radical cation and thus have a lower oxidation potential.^122,141,154,160^ From this mechanism, it can be assumed that the autocatalytic nature of the electropolymerization reaction arises from the ability of oligomer radicals to more readily react with monomers. An alternative explanation is that the oligomer radical acts as a true catalyst, facilitating the oxidation of a monomer, which then participates in coupling reactions.^6^

**Figure 5:**
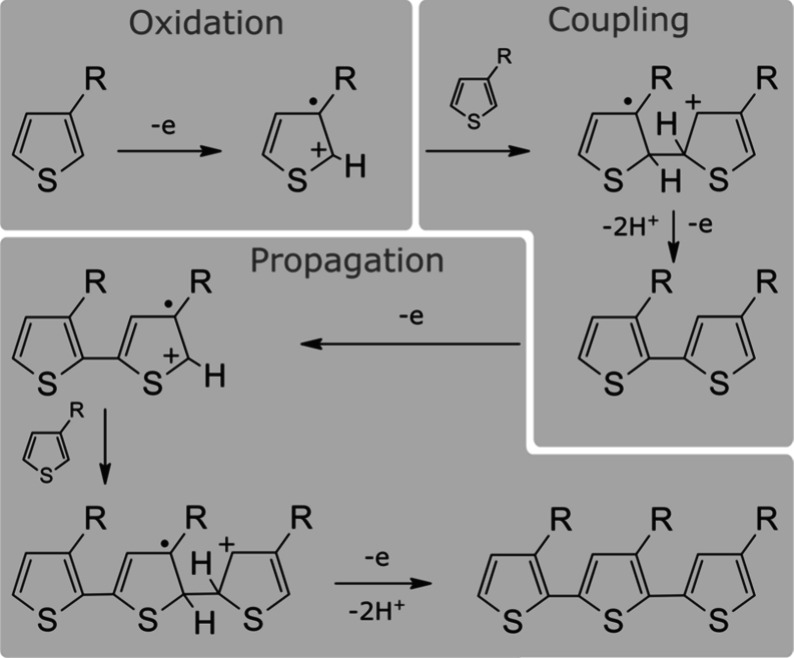
Alternative electropolymerization mechanism for thiophene electropolymerization, which posits that monomer radicals generated at the electrode interface are capable of reacting with neutral monomers in solution.^151^

#### 2.2.3 Mechanism Fusion

It is possible that the controversy over the mechanism is a result of multiple pathways existing in parallel or of different pathways existing for monomers, dimers, and oligomers. This possibility was proposed when density functional theory (DFT) calculations, applied to a mechanistic study of pyrrole polymerization, produced a more probabilistic interpretation of the mechanism (Figure 6).^128^ The results of this study indicate that monomer radical cations can react with monomer, dimer, and oligomer radical cations as well as neutral monomers and dimers. This view, however, does not account for the catalytic effects of oligomers in electropolymerization, as it presents the reaction of the dimer/oligomer radical cation with a neutral monomer as unlikely. A more recent work based on *in situ* spectroscopic examination of oligomeric intermediates suggests that while reactions of a radical oligomer and neutral monomer contribute to chain elongation, radical–radical coupling plays a terminating role.^139^

**Figure 6:**
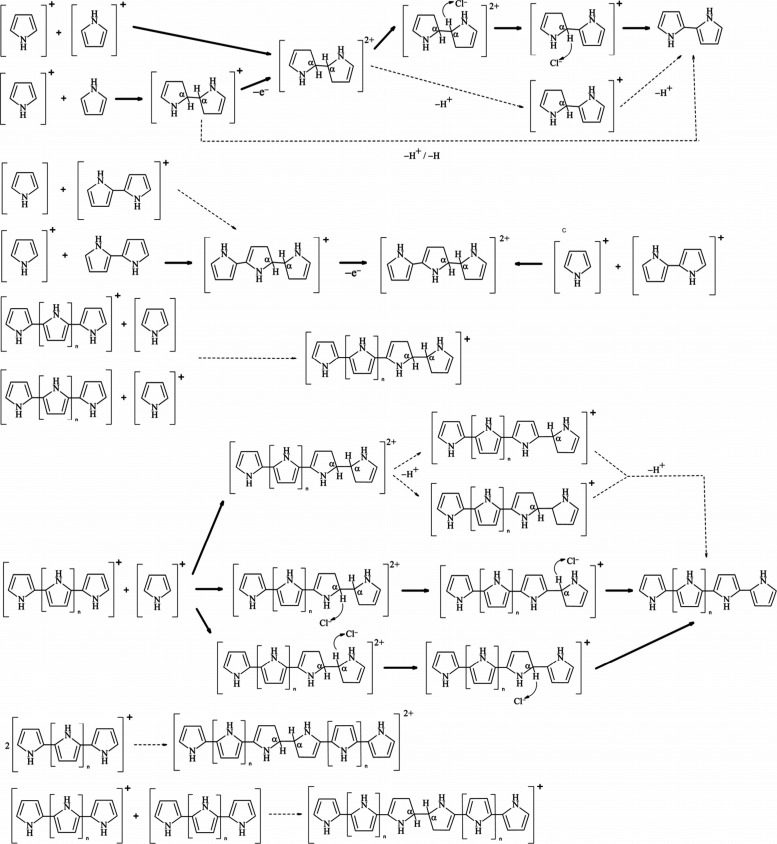
Mechanistic pathways of pyrrole electropolymerization. Dominant pathways are indicated with solid arrows while unlikely pathways are indicated with dashed arrows. Adapted with permission from reference 128. Copyright Elsevier, 2020.

### 2.3 Experimental Considerations

Given the long history and broad applications of electropolymerized conducting materials, the most commonly used electropolymerization techniques (i.e., potentiostatic, potentiodynamic, galvanostatic, and pulsed),^6,161^ as well as the specific effects of monomer concentration,^162^ and additives^6,162^ on the electropolymerization process have been thoroughly reviewed. Here, we will instead provide a set of design principles that are actionable and assist in decision making rather than focusing on optimizations that other researchers have made. This section includes a set of practical considerations in setting up an electropolymerization experiment.

#### 2.3.1 Electrodes

Conducting polymers can be electrically deposited on most conductive materials that are not corroded at the reaction conditions required for electropolymerization. Recently, even these boundaries have been pushed when poly­(*N*-ethylaniline) was deposited on copper, a notoriously electroactive material, through the use of a passivating agent to protect the copper surface during the electropolymerization process.^163^

The electrode type is generally chosen to suit the application or characterization technique of interest. The types of electrodes used for electropolymerization in the applicational fields reviewed here can broadly be classified into three categories—disc electrodes, thin film electrodes, and printed electrodes (Figure 7). High surface area electrodes like carbon fiber, metal foam, and nanomaterial-functionalized electrodes are often preferred for energy applications, which fall outside the scope of this review. The choice of electrode confers a set of benefits, limitations, and a selection of available materials.

**Figure 7:**
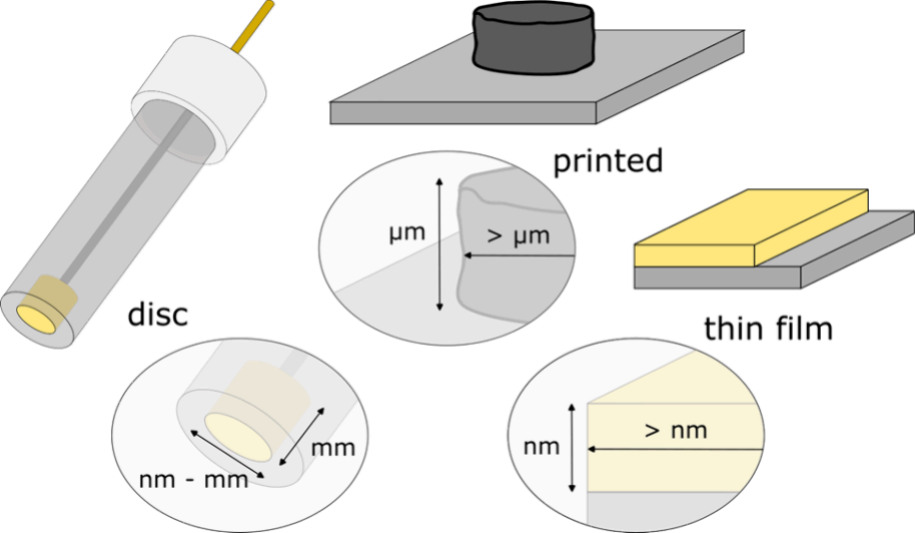
Application requirements play a main role in the selection of the electrode form factor. The most commonly used electrode form factors for the applications outlined in this review include the disc electrode, thin film electrode, and printed electrode.

Disc electrodes, generally consisting of a pellet of conductive material encased in an insulator, with diameters in the range of micrometers to millimeters and thicknesses in the millimeter range, have the longest history of use in electrochemistry. They are often preferred over wires, pellets, and sheets because they provide a well-defined surface area that can be regenerated through mechanical polishing and reused hundreds of times. Disc electrodes are especially suitable for mechanistic studies,^164^ in part because they can be integrated with a motor to produce the rotating disc electrode, which allows for simplified decoupling of mass transport and charge transport contributions to the current.^165^ Electrode diameters down to the nanometer scale can be obtained by a variety of techniques.^166–168^ While nanoscale electrodes are less reusable, they can be manipulated in three dimensions to provide highly localized analyte detection^169–171^ in sensing applications and can bypass mass transfer limitations^172^ in the study of the reaction mechanism and kinetics. The most common electrode materials used in this configuration include gold,^173^ platinum,^174^ graphite,^175^ and potentially glassy carbon.^176,177^

Thin films, deposited on a flat substrate by evaporation or sputtering, can be patterned and selectively insulated to produce electrodes and devices of any 2D geometry with features down to the nanometer scale in both thickness and lateral dimensions.^178^ Each application determines the optimal balance between lateral resolution, throughput, and instrumentation cost provided by the patterning approach. One extreme of this spectrum is represented by electron beam lithography, which provides for features in the nm range, while the other extreme is represented by simple patterning techniques like metal and insulator deposition through a shadow mask, with resolution limits in the tens of micrometers. The nanometer-scale thickness of the film renders these electrodes only moderately reusable because most available cleaning methods result in etching of the electrode material.^179,180^ Gold^181^ and platinum^182,183^ electrodes, deposited on an adhesion layer of chromium or titanium, are entrenched as the most common thin film materials used as substrates for electropolymerization. Specific applications and evaluation techniques have also benefited from the use of conducting polymer,^184^ optically transparent indium tin oxide (ITO),^185^ and nanostructured^186^ substrates.

Printed electrodes, often preferred for large-scale and single-use applications, can be manufactured by a wide variety of printing technologies. Each printing method is characterized by a range of accessible throughput and resolution values (Figure 8).^187^ While the resolution of printed electrodes, which is generally limited to tens of micrometers, does not compare to that of electrodes fabricated by subtractive patterning methods like photo- and e-beam lithography, printing is more cost-effective due to the reduced costs associated with materials, labor, and instrumentation.^188^ The development of inks is currently an active area of research, with rapid improvement in the diversity and performance of the materials that can be printed with high resolution in 2D and 3D.^189^ Some benefits of printed electrodes include a large selection of electrode and substrate materials and low materials and manufacturing costs.^190^

**Figure 8:**
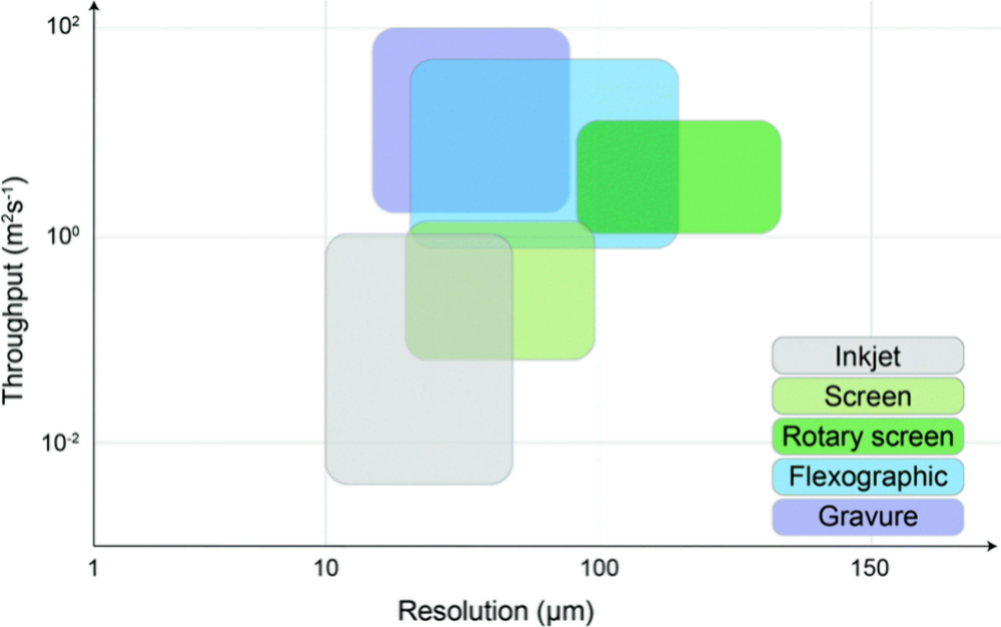
Throughput and resolution ranges of different printing technologies. Reproduced with permission from reference 187, Copyright The Royal Society of Chemistry, 2018.

#### 2.3.2 Electrode Pretreatment

Electrode pretreatment is often conducted with the aims of altering surface morphology, removing contaminants, or chemically activating the electrode. Swain provided an excellent, in-depth overview of pretreatment protocols for a variety of electrode materials.^191^ Each of the following methods offers unique advantages in preparing electrodes for electropolymerization and is chosen based on the type of contamination, the electrode material, and the type of electrode. In many cases, a combination of techniques is used to achieve a fully cleaned and activated electrode, ensuring high-quality electrochemical performance.

Mechanical polishing is one of the most straightforward and widely used methods for cleaning disc electrodes. It involves using abrasives, such as alumina or diamond slurries with particle diameters down to 50 nm deposited on a microfiber cloth to physically regenerate an electrode surface between experiments. Polishing in a figure-eight pattern while periodically rotating the electrode promotes the exposure of a flat, uncontaminated surface that is perpendicular to the electrode axis. After polishing, the particles are removed by sonication in an appropriate solvent.

Solvent cleaning involves soaking the electrode in organic solvents, most commonly acetone and isopropanol, and is often coupled to sonication. This method is effective in dissolving, desorbing, and mechanically removing environmental particulates and organic contaminants from the electrode surface. To maximize efficiency, the high-purity solvents should be purchased or purified through distillation and stored over activated carbon to remove impurities. This ensures that no additional contaminants are introduced during the cleaning process. Solvent cleaning is a noninvasive approach that can be applied to both disc and film electrodes, which preserves the electrode’s microstructure.

Gold and platinum metal electrodes are often cleaned by potential cycling in an aqueous sulfuric acid solution within a range that just exceeds the electrochemical window. During this process, the applied potential leads to the reversible formation and elimination of metal oxide layers as well as the oxidation or reduction of adsorbed organic residues. Electrochemical cycling leads to increased surface roughness, the relevance of which should be considered for the final application. It is a versatile and efficient approach, suitable for use immediately before an electropolymerization experiment.

Chemical etching, most commonly performed in piranha solution (3–4:1 H_2_SO_4_:H_2_O_2_) effectively dissolves surface layers and contaminants, exposing a fresh and clean electrode surface. It is the preferred cleaning method for ITO. Due to its aggressive oxidative properties, piranha solution is not compatible with many of the resins used to insulate disc electrodes and potentially the insulating layer in microfabricated devices. Like electrochemical cycling, chemical etching alters the surface roughness and can, in some cases, compromise the integrity of thin film metal electrodes.

Plasma treatment by inert or reactive ionized gases, such as argon, nitrogen, or oxygen, not only removes organic contaminants through oxidation to volatile constituents, but can also be used to alter the surface chemistry of the electrode and the supporting substrate by covalent addition of the reactive ions. UV-ozone treatment functions by a similar oxidative mechanism as oxygen plasma treatment. Deep UV light is used both to break covalent organic bonds at the sample surface and to generate highly reactive ozone that oxidizes the surface. When cleaning gold metal electrodes, treatment by oxygen plasma and UVO have minimal effects on the mass and surface roughness of the electrode.^192–194^

Beyond removal of contaminants that interfere with the electrical connection between the electrode surface and the electrodeposited film, electrode pretreatment can also be used to improve film stability. Delamination is a commonly observed mode of failure encountered in both mechanical and electrochemical stress tests of electropolymerized conducting polymer films.^195–197^ The instability of the interface is a major obstacle to the use of electropolymerized films in applications that require long-term use. Several physical and chemical solutions have been proposed to address this deficiency. Increasing the roughness of the metal electrode by chemical,^198,199^ electrochemical,^200^ or physical^196,200,201^ means produces an increase the interfacial area between the electrode and the polymer film, minimizing the effects of interfacial resistance and providing improved physical adhesion.

Chemical modification of the electrode and/or monomer to stabilize the electrode–film interface has also been shown to greatly improve film adhesion. For example, covalent anchoring of EDOT to a platinum electrode through diazonium chemistry,^202^ carboxylated EDOT to activated ITO surfaces,^203^ and an amine-functionalized PEDOT thin film electrografted to ITO to serve as a primer layer for a conductive PEDOT film^204^ have been demonstrated to improve the stability of the interface. A complementary approach, which takes advantage of the adhesive properties of polydopamine, led to improved adhesion of PPy to ITO surface by copolymerizing Py with dopamine.^205^ Thin films of polyurethane have also been shown to significantly enhance the adhesion of PEDOT, PPy, and PANI on both conducive and insulating substrates.^206^ Adhesion of electropolymerized conducting polymer to oxidizable metals like steel and iron has been shown to be improved by treatment with 10% aqueous nitric acid, which passivates the reactive metal interface while still allowing for monomer oxidation through the passivating layer.^207^

Interaction of the monomer with the substrate that supports a thin film or printed electrode plays an important role in determining the extent of polymer spreading away from the electrode during electropolymerization.^76,208,209^ Strong interactions between the monomer and the substrate have the effect of preconcentrating the monomer in the plane of the electrode and promote lateral spreading of the polymer film away from the electrode.

#### 2.3.3 Solvent System

The choice of solvent is particularly important in designing an electropolymerization experiment, as it affects the solubility, transport, and reactivity of the monomer and oligomer species, which affects the morphology and electrochemical behavior of the polymer film. For a given monomer or set of monomers, a solvent must be chosen that is chemically inert, compatible with the substrate, has a suitable electrochemical window, and in which the reactants are sufficiently soluble or able to be solubilized using surfactants. A solvent’s capacity for hydrogen bonding has the potential to either enhance^210,211^ or inhibit^212^ the rate of electropolymerization, depending on the identity of the monomer. Thiophene-based conducting polymers have historically been electropolymerized in organic solvents like acetonitrile, dimethyl sulfoxide (DMSO), dimethylformamide (DMF), and propylene carbonate, which were chosen based on their broad electrochemical windows and the ability to dissolve hydrophobic conjugated molecules. In contrast, PANI-based polymers are generally electropolymerized in acidic aqueous solvents.^213^ More recently, ionic liquids have been demonstrated as a suitable medium to support the electropolymerization of PEDOT,^214,215^ PANI,^216^ Py^217^ and PT.^218^

Solvent plays a major role in the morphology and properties of an electropolymerized film. Poverenov et al. conducted electropolymerization of EDOT in acetonitrile and propylene carbonate, showing that propylene carbonate leads to films with low surface roughness, high coloration efficiency, and high contrast ratio, which they attributed to the increased solubility of EDOT oligomers in PC, which facilitates the formation of longer polymer chains.^68^ Chiang et al. performed a similar study in a different set of solvents, comparing electropolymerization of EDOT in acetonitrile to that in DMSO and DMF.^219^ They observed that the conductivity increases 3-fold for films fabricated in DMSO, which they attributed to the conformational change of the PEDOT from the coiled benzoid to the more linear quinoid structure. Many similar studies showing the effect of solvent on electropolymerized PEDOT,^220^ PMDTO,^221^ PDOS,^222^ PEDOP,^223^ donor–acceptor polymers,^224^ poly­(thieno­[3,2-*b*]­thiophene),^225^ acetic acid modified polyterthiophene,^226^ and various copolymers^96^ have been reported.

Electropolymerization in ionic liquids (ILs) is a special case because ILs act as both the solvent and the electrolyte/dopant during electropolymerization. ILs are interesting in the practical sense because they are nonvolatile, nonflammable, stable at elevated temperatures, recyclable, and have a wide electrochemical window.^227,228^ Depending on the monomer used, electropolymerization may be benefited by the use of either aprotic (Py^229^) or protic (aniline^230,231^ ) ILs while some monomers, like thiophene, have been electropolymerized in both protic^232^ and aprotic ILs.^233–235^ When a protic IL is necessary to support electropolymerization, an exogenous proton source may also be introduced.^231^

There are several ways in which electropolymerization in ILs is different from that in organic/aqueous electrolytes. Some evidence has been presented that certain degradation mechanisms observed in aqueous electrolytes can be suppressed in ILs.^236^ Unlike electropolymerization in organic/aqueous electrolytes, both anions and cations have been suggested to integrate into the polymer matrix.^217^ Further, pure ILs exhibit high solution resistance due to their high viscosity, which leads to low ion diffusion compared to solubilized electrolytes.^227,237^

#### 2.3.4 Electrolyte

The size and charge of the ions in the electrolyte affects the nature of their interaction with the electrogenerated monomer, oligomer, and polymer radical, which in turn affects the reactivity and effective size of the monomer as well as the packing efficiency of the polymer.^238,239^ The counterions integrated within the electrodeposited OMIEC film additionally serve as the dopant, thus having an effect on the electrical properties of the film. Páramo-García et al. evaluated the effects of seven different anions (I^–^, NO_3_^–^, Br^–^, Cl^–^, ClO_4_^–^, SO_4_^2–^, F^–^) on the electropolymerization of PPy,^240,241^ finding that changing the identity of the anion in the supporting electrolyte produces films with thickness and capacitance values of spanning 4 orders of magnitude. In their discussion, they postulate that larger ions facilitate PPy nucleation at the electrode, leading to an increased growth rate. The concentration of the electrolyte can also affect the morphology of an electropolymerized film, which can then impact applicational figures of merit like enzyme loading and sensitivity in an enzymatic biosensor fabricated by electropolymerization.^242^ A study of electrochemically polymerized PEDOT films showed dopant-dependent effects on the thickness, dopant mobility, and ion exchange capacity of the resulting films.^243^ Currently, there is no widely applicable set of principles for selecting the appropriate electrolyte during electropolymerization. Thus, a screen of the electrolyte can be a simple solution if electropolymerized OMIEC films are not suitable based on the criteria set by the application.

#### 2.3.5 Substrate Interactions

For a given application, it may be advantageous to either encourage or restrict the lateral spreading of the electropolymerized OMIEC beyond the electrode from which it is formed. This is especially relevant when functionalizing high-density electrode arrays for neural recordings or connecting neighboring metal electrodes with OMIECs to fabricate resistive gas sensors and neuromorphic devices. Several groups have shown that lateral spreading is controlled by the interactions between the monomer and the insulating substrate that supports the electrode, presumably due to the accumulation of monomer in the plane of the substrate during electropolymerization.^76,208,244^ By functionalizing the monomer^76^ or substrate,^76,208,244–246^ changing the solvent,^208^ or introducing surfactants,^208^ these interactions can be enhanced to promote lateral spreading of a thin film away from the electrode. Substrate modification by hydrophobic silanes is a general approach to promote monomer–substrate interactions due to the hydrophobicity of the conjugated moieties of monomer precursors. Patterning of the silane can also be used to define the dimensions of the OMIEC channel between source and drain electrodes.^246^ Inversely, weakening the monomer–substrate interactions encourages OMIEC localization on the electrode^245^ or the growth of OMIEC wires between neighboring electrodes.^247^

One demonstrative example of using monomer–substrate interaction to control the spreading of electropolymerized conducting polymer can be seen in Figure 9. In this investigation, the authors evaluated the rate at which three different polymers, composed of identical conjugated backbones, but bearing anionic, zwitterionic, and cationic side chains, spread away from the active electrode at which electropolymerization occurs. A second grounded electrode is placed 30 μm away from the active electrode and allows for in situ conductance measurements. The pairing matrix of the three monomers with three different substrates, modified with silanes with different surface charge and polarity, showed that electrostatic and hydrophobic interactions between the substrate and the monomer impact both the lateral growth rate of the polymer film and the rate of conductance increase between the electrode adjacent electrodes.

**Figure 9:**
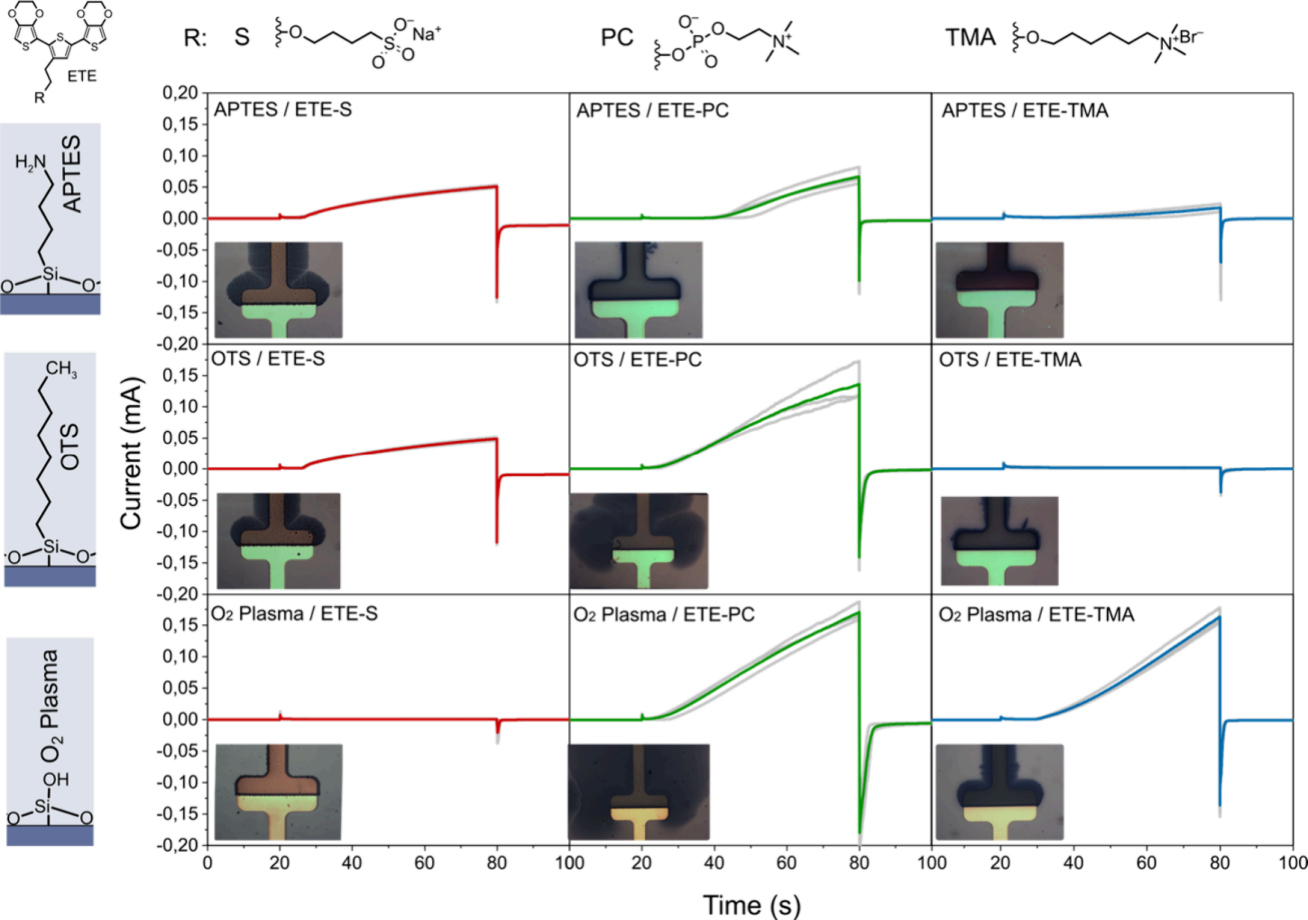
Monomer–substrate interactions. The pairing matrix between three differently charged ETE derivatives—negatively charged ETE-S, positively charged ETE-TMA, and zwitterionic ETE-PC—and three substrates modified to display different types of surface charge—partially negative oxygen plasma-cleaned silicon, partially positive APTES, and hydrophobic OTS. The electropolymerization was carried out at a voltage proportional to each independent monomer oxidation potential and allowed to proceed for 60 s. During this time, the current between the electrode at which electropolymerization was carried out and an adjacent electrode placed at a distance of 30 μm was recorded (plots). The inset images show the extent of polymer spreading at the end point of electropolymerization. This image was adapted from reference 76 (CC BY 4.0).

#### 2.3.6 Morphology Control

The surface morphology and internal structure of an electropolymerized conducting film is of paramount importance for most applications. For example, nanostructured materials have been shown to facilitate cell adhesion,^248^ affect protein expression,^249^ and establish conformal contact with the cell surface, which facilitates the extraction of electrical signals.^250^ In OECT applications, surface roughness reduces the effective thickness of a channel produced by electropolymerization, which in turn reduce the transconductance of a device, but also the time constants of ion migration into and out of the channel.^251^

The implementation of hard and soft templates to control film morphology is an active and fascinating field of research,^6,254–259^ but is too specialized and diverse to fit within the scope of this review. Instead, we center our discussion on how experimental conditions can be altered to produce electrodeposited films or long-range wires—two of the most common OMIEC structures employed in bioelectronic and neuromorphic applications. A simplified rule that determines the morphology of an electrodeposited material is that compact films are formed in the reaction-limited regime, when the rate of monomer depletion by means of oxidation at the electrode is insignificant compared to the rate of monomer replacement. In contrast, dendrites and nanowires are formed when experimental conditions are more conducive to the mass transfer-limited regime, when reaction rates exceed the rates of monomer replacement. The morphology of an electrodeposited material is then dictated by the distance from equilibrium conditions defined by these processes (Figure 10a).^252^ This behavior is observed in a broad variety of natural phenomena, like bacterial colony growth,^260^ branching of lung epithelium,^261^ and geological deposits.^262^

**Figure 10:**
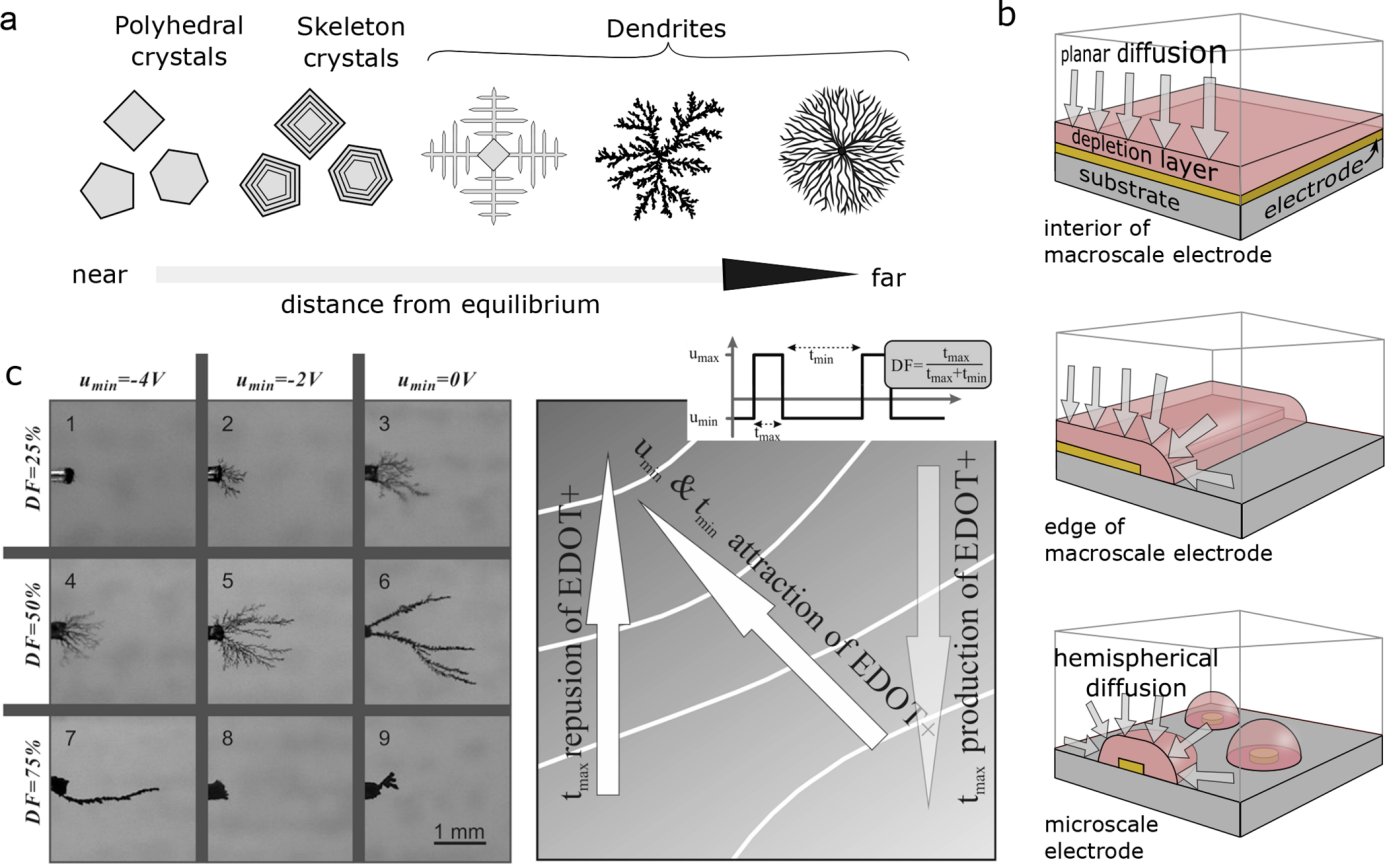
Morphology control. a) The morphology of an electrodeposited material is dictated by the distance from equilibrium conditions defined by monomer consumption at the electrode and the delivery of new monomer to the electrode. Panel a adapted from reference 252 Copyright American Chemical Society, 2007. b) The flux of monomer delivery to an electrode by diffusion depends on the size and geometry of the electrode.^253^ c) The branching behavior of an electropolymerized OMIEC can be controlled by varying the potential waveform applied to the electrode to modulate the rates of monomer oxidation and migration of the cationic reaction intermediates. Panel c adapted from reference 142 (CC BY 4.0).

A lot of the work establishing the relationship between experimental conditions and film morphology was performed on electrodeposited metals^252,263–265^ using the general principles of diffusion-limited aggregation.^266,267^ The models used to describe this system can generally be extended to approximate the electropolymerization of OMIECs, as both systems involve a soluble reactant undergoing heterogeneous electron transfer at a biased electrode to produce an insoluble conductive product that deposits on the electrode. However, there are two main differences between the systems that preclude the drawing of a direct analogy between the two processes. First, the conversion from a soluble reactant to an insoluble product is more gradual for OMIEC electropolymerization, as the reaction of an OMIEC monomer must generally progress through several steps involving soluble intermediates before arriving at an insoluble product.^110,268^ (Section 2.2.2) Further, monomers and soluble oligomeric intermediates are converted to radical cations as a consequence of oxidation at the electrode and are thus subject to an electrophoretic mobility that differs from that of their uncharged counterparts. Nevertheless, the diffusion-limited aggregation framework is helpful in conceptualizing how experimental conditions affect the morphology of electrodeposited OMIECs, even though mass transfer is complicated by different rates of migration for monomers and soluble oligomeric reaction intermediates.^269–271^

There are a variety of experimental conditions that impact reaction and mass transfer rates, the balance of which then defines OMIEC morphology. In the simplest case, reactants are depleted at the electrode interface at a rate that is controlled by the applied potential that exceeds polymerization onset potential of the reaction. In real systems, however, the deposition of conductive material may lead to local and dynamic changes in the work function^272^ of the electrode, inhomogeneities in the electric field,^273^ or a potential drop within the deposited material,^274^ which can all lead to inhomogeneities in the reaction rate at the electrode surface. The effective reaction rate can also be artificially modulated by implementing an alternating or pulsed potential waveform that limits monomer depletion to a defined length of time within a period.^142,273,275^

The rate at which reactants are replaced at the electrode–electrolyte interface is controlled by several drivers of mass transfer: diffusion, migration, and fluid dynamics. Diffusion rates vary depending on the effective radius of the monomer or monomer aggregate (which includes counterions and the solvation shell),^276,277^ the concentration gradient of the monomer between the electrode surface and in bulk solution,^278^ and the viscosity and temperature of the solvent.^276^ The dimensionality of diffusion is also defined by the size (macroscale vs microscale) and the local geometry (center, edge, or corner) of the electrode (Figure 10b).^253^ For example, the interior of a macroscale thin film electrode would be subject to planar diffusion of monomer to electrode surface while the edge of the same electrode^253^ would be subject to radial diffusion. A microscale electrode with a size comparable to the diffusion layer thickness^279^ would be subject to hemispherical diffusion. Migration mainly affects charged species encountering an electric field at low electrolyte concentrations.^280^ In the case of electropolymerization, this includes the cationic monomer radicals and soluble oligomeric intermediates generated at the anode^140^ as well as monomers exhibiting charged side chains.^42^ The rates of migration are influenced by 1) the magnitude and direction of the electric field, which is influenced by the applied potential, electrode configuration, and the ionic conductivity of the electrolyte, 2) the charge of the traveling particles, and 3) factors contributing to ‘friction’ between the charged species and the electrolyte, which include the size and shape of the charged particle, the viscosity of the electrolyte, and the presence of solid matrix elements. Eickenscheidt et al. have provided a systematic demonstration of how reaction and migration rates can be modulated to control the branching behavior of electropolymerized PEDOT (Figure 10c).^142^ Hydrodynamic conditions attained by mixing, flow, or electrode rotation accelerate the delivery of monomers to the electrode, but also accelerate the removal of soluble radicals from the electrode surface.^281–283^ For this reason both the Faradaic electropolymerization current and the thickness of the resulting film are lower at these conditions. An interesting solution to improving electropolymerization efficiency by hydrodynamic flow was proposed by Leventis et al.^284^ They showed that due to the paramagnetism of radicals generated during the reaction, a magnetic field can be used to exclusively affect the flow of reaction intermediates either toward or away from the electrode independently of the electrolyte.^284^

OMIEC films are widely used due to their excellent performance in biosensors and biointerfacing applications (Sections 3.1 and 3.2). As mentioned previously, the optimal range of film porosity, conductivity, and surface roughness depends highly on the intended application. The effect of electropolymerization conditions on film morphology varies for different OMIEC precursors, especially due to the entanglement of reaction and migration rates as a function of the applied potential, and must be experimentally optimized to suit each monomer and application. Many examples of this process exist in the literature and several recent examples are highlighted here. The morphology of thiophene-based materials can range from dense, compact films^67^ to films of globular nanostructures. In organic solvents, higher surface area structures like trees and tubes can be attained by reducing trace water at the electrode between electropolymerization cycles to form microscale hydrogen bubbles.^285,286^ Najafisayar et al. investigated the effects of the current density, frequency, and duty cycle of a pulsed current electropolymerization experiment, finding that shorter pulses produced a film with finer structure, improved ionic penetration into the film and, consequently, higher capacitance.^287^ PANI is generally electropolymerized in aqueous solvent systems. Due to the presence of an acidic proton, PANI morphology is highly sensitive to the pH of the reaction mixture. At different pH values, Peng et al. observed the formation of PANI nanowires, coral-like structures, and flakes.^288^ The effect of the pH on PANI morphology is likely due to its effect on the proportions between oligomers and nucleates during electropolymerization.^289^

Electrogenerated OMIEC wires have recently been shown to be a promising synaptic mimic in emerging neuromorphic applications (Section 3.3). In an early work on electrogenerated PEDOT wires that bridge neighboring electrodes, Das et al. used high constant voltages (10 V) to induce the formation of PEDOT wires between flat and pointed electrodes. They postulated that wires only form in the presence of high electric fields due to the migration of reactive species along the field.^290^ Musumeci et al. further showed that the rapid nucleation, diffusion-limited growth and electromigration processes observed in high electric fields promote the formation of aligned conductive wires that bridge the gap between neighboring electrodes.^291^ In other words, the process of wire formation can be attributed to electropolymerization of a directed stream of soluble active intermediates that forms when electrode features such as a sharp tip or conductive nuclei concentrate excess charge and induce a strong local electric field.

Wires can also be attained using significantly lower anodic potentials when a pulsed or alternating voltage is used. Several groups have investigated and modeled the formation of OMIEC fibers grown in a pulsed electric field to assess the origin of this effect. Koizumi et al. evaluated the growth of PEDOT fibers at wireless electrodes under pulsed deposition conditions.^71^ They report that DC voltage and low frequency pulsing produces films and clusters while high frequency pulsing produces fibers. From their observations, they postulate that cationic polymers formed at the electrode migrate away from the anode along the electric field, which leads to a decrease in the effective concentration of active species at the electrode surface as a function of time. In a follow-up publication from the same group, Ohira et al. showed that reducing the dimensionality of the diffusion by placing the wires in a vertically confined cell with vertical dimensions of 50 μm or less reduces the branching by enhancing monomer depletion around the body of the growing PEDOT electrode.^292^ A systematic approach to investigate pulsed polymerization to grow conductive wires was taken by Eickenscheidt and colleagues, wherein they investigated the effects of both the duty factor and the negative offset of the pulse. From their findings, they implicate the balance between the surface concentration of PEDOT radical cations and the diffusion length of the radical cations during the anodic pulse is the primary determinant of wire morphology.^142^

Kumar et al. modeled fiber formation in a more complex system, wherein potential pulses are used to electropolymerize EDOT in an aqueous solution of polystyrenesulfonate (PSS) electrolyte and a sacrificial benzoquinone, which provides the cathodic counter-reaction.^275^ The model consisted of charged particles traveling in a two-dimensional spatiotemporal potential map generated by two opposing wire electrodes over the course of a pulse sequence. When a particle approached within a certain distance from either electrode, it was immobilized at that electrode with a selected probability. They found quite good agreement between the model and experimental data, replicating the frequency-dependent tendencies of dendrite density and time to form a connection between opposing electrodes. The model was later refined to incorporate changes in the potential map as a result of the growth of conductive dendrites.^273^

#### 2.3.7 Counter-reaction

When designing an oxidative electropolymerization experiment, some consideration should be given to the reduction process that serves as the counter-reaction, especially in a two-electrode system where the cathode serves the function of both counter and reference electrodes.^293^ The cathodic reaction should not be rate-limiting or involve reactants or products that affect the anodic reaction. The hydrogen evolution reaction is the most commonly used counter-reaction in aqueous systems, as a platinum counter electrode serves as an effective catalyst by facilitating the reduction of adsorbed protons to hydrogen gas. Nickel^294–296^ and steel^297^ can be used as inexpensive substitutes. The main benefit of hydrogen evolution as a counter-reaction is the abundance of reactants, especially in acidic environments, and the removal of the products from the reaction solution, which minimizes interference with anodic reactions^293^ and the impact on the reaction equilibrium caused by the cathodic reaction. Since protons serve as a reactant, the redox potential of this reaction, like that of oxidative electropolymerization, is also sensitive to pH. For this reason, when studying electropolymerization conditions that affect the pH, some measures should be taken to independently assess the effects of these conditions on the hydrogen evolution reaction. In cases where the counter-electrode is not easily interchangeable, such as in bipolar electropolymerization experiments, a metal ion^298^ or an organic redox molecule^71,256,292^ can implemented as an electron acceptor at the cathode. In the absence of an intentionally introduced redox process that supports charge neutrality, reduction of the solvent or dissolved gases will proceed when a sufficiently high potential is applied between the working and counter electrode.

#### 2.3.8 Open Circuit Potential

The open circuit potential (OCP), or the potential difference between the working electrode and the reference electrode when no current flows through the system, is a useful parameter to consider when evaluating the effects of a set of conditions on the electropolymerization onset potential. While the OCP can be measured quite simply with a potentiostat or even a voltmeter, comparing the measured value to the theoretical OCP of a system requires a thorough understanding of the reactions and processes occurring in the electrochemical cell. A clear theoretical overview and some common pitfalls in calculating the OCP has been outlined by del Olmo et al.^299^ Since the OCP is proportional to the difference between the chemical potentials of all of the reactions occurring at the anode and those occurring at the cathode, it is important to evaluate the OCP in the absence of monomer to identify whether the shift in the onset potential is attributed to a change in the thermodynamics of the electropolymerization process or of the other reactions occurring within the electrochemical cell. This is especially true when current may be passing through the reference electrode (e.g., two-electrode systems, pseudoreference electrodes like Ag wire or Ag/AgCl pellet, leaky single-junction reference electrodes). In practice, the OCP can be affected by the dissolved oxygen,^182^ electrolyte concentrations,^300^ pH,^300,301^ and reducing agents.^300^

### 2.4 Instrumentation and Electrode Configuration

The most basic configuration of an electrochemical cell that can be used to drive electropolymerization requires two electrodes connected by a power source and suspended in a solution containing monomer and electrolyte.^302^ Monomer oxidation will be initiated at the anode when the potential applied between the two electrodes exceeds the potential difference between this oxidation reaction and the reduction counter-reaction occurring at the cathode. Here, we use illustrative examples where voltage is controlled while the current is measured, but most modern instrumentation is also capable of functioning in the galvanostatic mode, such that the current is controlled and the voltage difference is measured.

#### 2.4.1 Two- and Three-Electrode Configurations Coupled to a Potentiostat

The instrumentation required for conducting and characterizing the electropolymerization process varies in complexity, ranging from electronically sophisticated equipment, such as a potentiostat, to more basic power sources (Figure 11). In the simplest two-electrode configuration with a basic power source, voltage drops both across the solution and at the cathode-solution interface, making it difficult to extract the exact potential drop at the anode, where the reaction of interest occurs. To resolve this issue, a potentiostat can be used in the three-electrode configuration to designate the voltage control function of the cathode to the reference electrode (R) and the current flow function to the counter electrode (C). The potentiostat limits the current flow through a reference electrode by placing it outside of the circuit and compensates for the measured voltage difference between working and reference electrodes using a control amplifier. Coupled with a stable reference electrode, a potentiostat can accurately apply the potential waveform input by the user. The three-electrode configuration is thus preferred for characterizing the kinetic and thermodynamic properties of the electropolymerization reaction due to its ability to selectively monitor the current attributed to electrochemical processes occurring at the surface of a working electrode (W).

**Figure 11:**
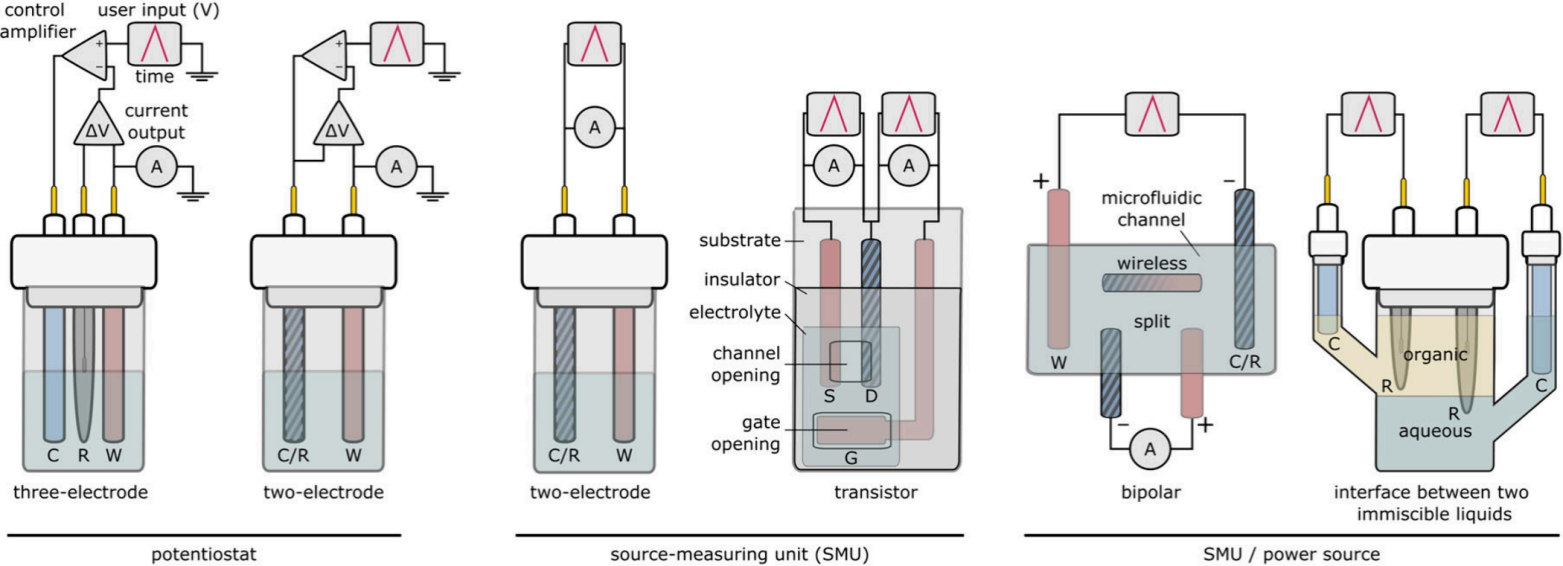
Schematic of different electrode configurations and electrochemical cells that can be used to electropolymerize OMIECs. Each illustration depicts the working (red) electrode at which electropolymerization occurs as well as the reference (black) and counter (blue) electrodes, where applicable. The schematic indicates the terminals at or across which a potentiodynamic input (nonstatic voltage) is applied by the symbol labeled ‘user input’ in the leftmost configuration whereas the terminals across which the current is measured are indicated by a circle surrounding the letter ‘A’. While a potentiodynamic input function is depicted here, constant voltage and constant current inputs are equally common.

A two-electrode configuration can likewise be implemented on a potentiostat by shorting the counter and reference leads. In this way, the current passing through the reference electrode will produce the above-mentioned changes in the interfacial potential of both the working and reference electrodes, which will be difficult to deconvolute. The effects of this complication can be minimized by ensuring that the joint counter/reference (C/R) electrode has a much higher surface area than the working electrode, that the counter-reaction is not rate-limiting, and that the counter-reaction does not significantly affect the composition of the electrolyte.

#### 2.4.2 Transistor Configuration

While a potentiostat is primarily designed to monitor the electrochemical reaction happening in solution, a source-measuring unit (SMU) is designed to accurately measure circuit behavior in response to an electrical signal. SMUs therefore prioritize high temporal and measurement precision. The main difference between the potentiostat and SMU is that the SMU does not provide feedback to a control amplifier based on the true voltage difference measured between the two electrodes. As such, SMUs are used more frequently to monitor in situ electropolymerization of OMIECs on devices and the characteristics of the devices thus formed. The transistor configuration comprises two independent SMUs connected to the prepatterned source (S) and gate (G) electrodes, with a common counter (ground) electrode connected to the drain (D). In the specific case of electropolymerization, the definition of the source and drain electrodes differs from the standard definition used for p-type OECTs (grounded source). This is because when electropolymerizing in the transistor configuration, a positive voltage is applied to drive the oxidation reaction. In this case, the positive terminal is the source of the charge carriers (holes) while the grounded terminal acts as the drain. Openings in the insulating layer covering these electrodes define the dimensions of the channel and the gate. In this configuration, an OECT can be formed by electropolymerizing OMIEC materials to form a channel between the source and drain electrodes.^76,303^ Alternatively, an OMIEC can be electropolymerized at the gate of a device with a prefabricated channel. The transistor configuration can be used to both fabricate and measure the OECT device.

#### 2.4.3 Bipolar Configuration

In the past decade, the bipolar electrode configuration, which relies on the polarization induced at wireless electrodes situated in an external electric field, has also been introduced as a means of driving electropolymerization of conducting polymers.^71,228,304–307^ In a bipolar electrochemical cell, a wireless electrode is situated in an electrolyte between two driving electrodes that are connected to an external power supply.^308^ In a highly resistive electrolyte, the potential drops more or less uniformly between the driving electrodes, which both impedes redox reactions at the driving electrodes and produces a potential gradient across the bipolar electrode in the direction of the electric field.^309,310^ The effective potential difference across a bipolar electrode, which can be used to drive oxidation reactions at one end of the electrode and reduction reactions at the other end, is proportional to the electrolyte resistance and the electrode length.

There are several ways that the experimental configuration can be arranged in a bipolar experiment. As noted, a high solution resistance is required to maintain a linear potential drop across the electrolyte. There are two approaches that can be used to satisfy this condition. The first is limiting the ionic conductivity between the driving electrodes by reducing the ionic strength of the electrolyte.^311^ Alternatively, a capillary or microchannel can increase solution resistance through geometry.^309,312^ Bipolar electrodes can be either continuous, where the entire electrode is immersed in the electrolyte, or split, where only the electrode termini are immersed in the electrolyte, which allows for measurement of the current between the termini.^313^

The theoretical framework of the bipolar effect on wireless porous electrodes was described by Richard Alkire in 1973.^314^ The research within this field at that time was mostly aimed at developing more effective electrochemical reactors,^315–317^ but new interest arose when bipolar electrochemistry was shown to be useful in modifying conductive objects that are difficult to contact either due to their size, number or inclusion in a complex device geometry.^305,318^ Several groups have used bipolar electrochemistry to asymmetrically functionalize nanomaterials and microparticles in suspensions and on surfaces.^306,312,319^ Recently, Kuhn et al. have advanced this technique by immobilizing the conductive particles in a hydrogel positioned between the driving electrodes, which allows for precise control of the orientation of the electric field relative to the stationary particles and the formation of complex patterns of electrodeposited materials on the particles.^320^ This three-dimensional addressing has, however, not yet been demonstrated for conducting polymers. The bipolar configuration is also uniquely suited to generate a continuous potential gradient along a wireless electrode.^306,321^ In this way, it can produce a rapid screening of the effects of the applied potential on the electropolymerization process using a single power supply. The few drawbacks of bipolar electrochemistry include the use of high voltages in the electrodeposition and a large parasitic current, which leads to reactant depletion and power inefficiency.

The harvesting of pressure-induced potentials to drive electrochemical reactions is an interesting subcategory of bipolar electrochemistry because it allows one to bypass an electrical voltage source entirely. Just as a voltage applied across the length of a charged capillary induces an ionic current at the capillary wall due to the flow of the EDL, so does inducing the movement of the EDL by applying a continuous flow through a charged capillary generate a voltage. As shown by Dumitrescu et al., this electrical potential can be used to drive a pair of electrochemical reactions at opposite ends of a bipolar electrode and can generate potentials as high as 8.1 V across a microfluidic channel 4.5 mm in length.^322^ This approach was further explored by Iwai and colleagues, who use PEEK tubing packed with cotton wool to generate both a large pressure drop and a high concentration of stationary charge within the channel.^323^ These devices were shown to generate a potential of 2–3 V across the channel, which is enough to drive the polymerization of Py and EDOT using flow rather than a power supply.^323^

#### 2.4.4 Electropolymerization at the Interface between Two Immiscible Electrolyte Solutions

One benefit of electropolymerization as a means of modifying conducting surfaces is that the polymer film adheres quite well to the electrode compared to deposition by spin-coating. This set of attributes also renders electropolymerized conducting polymers difficult to study in isolation as free-standing films. In contrast, electropolymerization at the interface between two immiscible electrolyte solutions (ITIES)^324–326^ can be used to form free-standing conducting polymer films.^327,328^ In the ITIES configuration, two sets of reference and a counter electrode pairs, are positioned at either side of a liquid | liquid (L | L) interface that is formed by an organic and an aqueous layer. Since the electrolytes in either phase cannot cross to the opposite phase, the L | L interface becomes polarized when a potential is applied between the counter electrodes in each phase. The potential difference across the L | L interface can then be used to drive the interfacial electron transfer between an oxidant in one phase and the monomer precursors in the other phase. Depending on the nature of the oxidant, either neat polymer films^328^ or films with embedded metal nanoparticles^329–331^ can be produced at the interface.

There are several distinctions between electropolymerization of conducting polymers on solid electrodes and at the ITIES. First, ITIES does not rely on a substrate for support and is therefore not subject to strong adhesion to a substrate or substrate-templated surface defects. This results in the formation of pure, free-standing, defect-free films, which are not currently possible to produce by other methods. Furthermore, the double layer of the interface between two liquids is electrochemically distinct from the double layer formed at a solid electrode. The ionic structure at the interface resembles two ionic monolayers of opposite charge, which shields the remaining electrolyte from the interfacial potential, prevents the formation of the diffuse double layer, and thus confines the potential drop to a very confined region.^324,332^

### 2.5 In Situ Characterization

An in situ experiment is defined here as one that wherein the characterization can proceed either simultaneously with the electropolymerization or in the same region of interest while electropolymerization is paused, thus minimizing the effects of sample handling on the measurement. This approach ensures that fine structures at the film surface remain undisturbed by external factors such as mechanical disturbance or solvent evaporation. *In situ* characterization can be split into two distinct categories—characterization of the reaction and characterization of the film properties. Characterization of the reaction allows one to extract information about the thermodynamics of monomer oxidation, lifetime of the intermediates, and the reaction mechanism. In contrast, in situ characterization of the properties of an electropolymerized film allows for real-time monitoring of various orthogonal parameters, like mass, conductance, capacitance, topography, dielectric properties, and vibrational energy modes over the course of the film growth process, leading to an improved understanding of the deposition and growth of the OMIEC films. Methods used to characterize the thermodynamics and kinetics of the reaction as well as those used to assess film properties will be described here while methods used to carry out more fundamental investigations regarding the reaction mechanism are outlined in Section 2.2.

#### 2.5.1 Cyclic Voltammetry

Cyclic voltammetry (CV) has a long history of being used to characterize electrochemical reactions^333^ and is the most widespread method used to study OMIEC electropolymerization.^334^ CV is a three-electrode electrochemical technique wherein the potential of the working electrode is linearly and reversibly cycled between two predefined values while the current through the electrode is recorded. In a cyclic voltammogram, the current is plotted as a function of the applied potential. An excellent practical guide to selecting experimental conditions, understanding the underlying electron transfer processes, and interpreting CV data has been constructed^335^ and recently updated.^336^

CV is the most common representative of the potentiodynamic approach to electropolymerization because it is historically well-established in electrochemical research. The real power of CV, however, lies in its capacity to provide thermodynamic, kinetic, and mechanistic information about an electrochemical process. The most attractive feature of OMIEC deposition using CV is that it provides somewhat decoupled information about Faradaic processes, which involve charge transfer between the electrode and species that are either dissolved in the solution or deposited at the electrode surface, and non-Faradaic processes, which instead are a result of the accumulation of ions at either side of the interface. These attributes often make it possible to dynamically track the properties of both the reaction at the electrode surface and properties of the OMIEC film *in situ*.

Primarily, there are three processes that contribute features to the voltammogram of a conducting polymer film undergoing electropolymerization by CV: 1) polymer oxidation, 2) polymer charging and discharging, and 3) monomer oxidation. Figure 12 shows a qualitative sketch of the decoupled features observed in a voltammogram of a smooth electrode immersed in a monomer solution that produces an insoluble p-type polymer film upon electropolymerization. In the first cycle, absent an existing OMIEC layer or significant surface roughness, only the monomer oxidation contributes significantly to the observed current (Figure 12a). In the depicted case, one electron is transferred from the monomer to the anode to produce highly reactive monomer radical cations at the electrode surface. At sufficiently high monomer concentrations, the radical monomer cations react rapidly and spontaneously to form oligomers that precipitate at the surface of the electrode, producing a CV signature that is characteristic of an irreversible electron transfer process.

**Figure 12:**
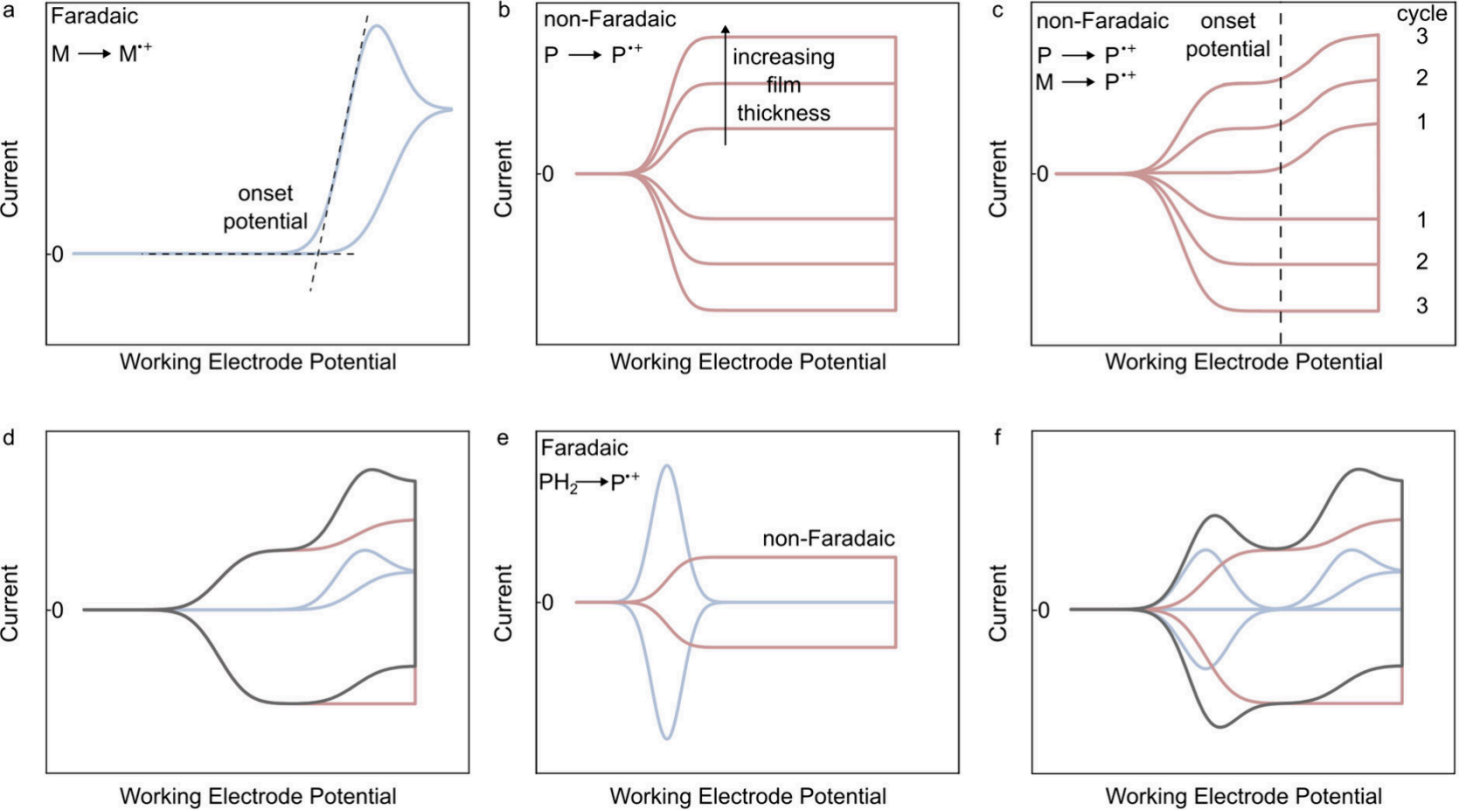
Idealized schematic illustrating the different processes contributing to the total current observed during *in situ* characterization of the electropolymerization reaction by cyclic voltammetry (black). Voltammograms capture both Faradaic currents (blue), which result from charge crossing the electrode–electrolyte interface, and non-Faradaic currents (red), which result from charge accumulation at the electrode–electrolyte interface.

The first linear sweep of the voltammogram is used to characterize the onset potential of electropolymerization, which, at sufficiently slow scan rates, reflects the thermodynamic energy barrier to monomer oxidation at the selected set of conditions. The onset potential has no universal definition, but is most often determined either as the potential at which the current surpasses a certain threshold or as the potential at which the lines fitted to the capacitive region and the Faradaic region intersect (Figure 12a, dashed lines).^337^ CV can also be used to extract the kinetics of monomer oxidation.^338^ On the reverse scan, the current drops rapidly to zero due to the depletion of the monomer from the surface of the electrode. Due to the short lifetime of the intermediates, a reduction peak can be observed only at fast scan rates, which allows for the determination of the lifetimes of the reaction intermediates.^111,339^ While the voltammogram depicted in Figure 12a is a descriptive sketch of an irreversible one-electron process, experimentally obtained voltammograms often exhibit departures from ideality, such as the nucleation loop observed in the first scan^340^ and a shifting of the monomer oxidation peak with subsequent cycles due to changes in the energy level of the electrode on which electropolymerization occurs.^341^

After the potential applied to the electrode surpasses the onset potential in the first CV cycle, a thin polymer film deposits on the surface of the electrode and contributes a capacitive signature to the voltammogram. The exact origin of OMIEC capacitance varies for different materials and is an active topic of discussion.^26,342,343^ There are two proposed models for the capacitance observed at OMIECs—the non-Faradaic EDL model and the Faradaic pseudocapacitive model. The principles underlying both of these models are highly relevant in energy storage and are expertly explained in a review by Costentin et al.^344^ For example, while PANI is generally accepted to behave as a pseudocapacitor, PEDOT exhibits behavior consistent with EDL model.^28^ For both types of capacitance, the polymer film acts as an insulator below the polymer redox potential due to the absence of carriers (polarons and bipolarons) that are required to transport charge through the film (Figure 12b, e). For a non-Faradaic capacitor, there is a smooth transition between the nonconductive and conductive regions of the voltammogram, after which the signature of the polymer resembles a typical EDL capacitor (Figure 12b). Since OMIECs exhibit volumetric capacitance,^345^ enabled by ion penetration into the bulk of the film, the capacitance increases proportionally to film thickness in the potential range where the polymer is in its oxidized state and can be used to estimate the thickness in situ. As additional layer material is deposited in subsequent cycles, the capacitance also increases at potentials above the electropolymerization potential, most prominently in the anodic sweep (Figure 12c). The contributions from the Faradaic and non-Faradaic processes combine to produce the resulting voltammogram (Figure 12d).

Pseudocapacitive polymers are characterized by one or more Gaussian peaks that are symmetric with respect to the *x*-axis, which is a signature of a reversible surface-confined redox process (Figure 12e).^346^ Because the polymer has a longer conjugation length that can better stabilize charge, the redox potential of the polymer is almost always lower than that of the monomer (Figure 12f).^6^ The height of this peak is proportional to the number of ion-accessible redox sites within the polymer film while the width of the peak depends on the microenvironment distribution of these sites. Until recently, it was thought that the capacitive behavior of these polymers originates exclusively from the charging of the embedded redox sites.^347^ However, extensive study of PANI polymers indicates that non-Faradaic behavior also contributes to the capacitance at anodic potentials past the Faradaic oxidation potential.^27^

There are several departures from ideality that would result in a significant deviation of the voltammogram from those presented here, including solubility of the oligomers, migration of charged monomers away from the electrode, disequilibrium of diffusion at the beginning and end of CV, multiple redox sites and microphases within the polymer, dissolved electroactive species (most commonly oxygen at ambient conditions), and slow kinetics of ion penetration into thicker films.

#### 2.5.2 Conductance Measurements

Conductivity, or the quantification of the ease with which charge passes through a given material, is an essential figure of merit used in benchmarking OMIEC films. Since conductivity is a material property, the measured current passing through the material at a given voltage (conductance) has to be normalized by the dimensions of the material being measured. The dimensions of an electropolymerized film can be difficult to assess, however, when the surface roughness of the film is on the scale of the total film thickness. Nevertheless, in situ conductance measurements can provide useful insights into the growth of an electropolymerized OMIEC film between two or more electrodes. For those interested in a more detailed analysis of the literature regarding in situ conductance measurements of electropolymerized films, the review provided by Salinas et al. serves as an exceptional resource on the topic.^348^

To measure conductance in situ, an OMIEC film has to be electropolymerized such that it bridges the gap between two or more electrodes. This can be accomplished, for example, using the transistor configuration described in Section 2.4.2. While electropolymerized films had been grown between microfabricated metal contacts for several years prior,^349–352^ the earliest conductance measurements performed during electropolymerization relied on a set of platinum foil electrodes separated by an insulating mylar film and embedded in an epoxy resin, which produces band electrodes with a defined spacing on the scale of tens to hundreds of micrometers.^353,354^ *In situ* conductance measurement requires a potentiostat with two independent channels or two SMUs such that one channel applies a small voltage and measures the current between the source and drain electrodes while the other channel drives electropolymerization by applying a voltage that is above the polymerization onset value between one of the S/D electrodes and a ‘gate’ electrode immersed in the solution. A simplified version of this technique, where the electrode providing the counter-reaction is grounded along with one of the S/D electrodes and the voltage that drives the electropolymerization is applied at the other S/D electrode, requires only a single channel to both drive the electropolymerization and measure the current between the bands.^42^

Electrochemically depositing OMIECs across a nonconducting gap between electrodes requires some engineering of the substrate surface to improve the speed and reproducibility of polymer spreading.^76,355^ Nishizawa and colleagues showed that modifying the substrate with a hydrophobic silane increases the rate of the lateral growth of PPy away from the electrode, promotes the growth of thin and even films, and can even be used to selectively pattern the surface between the two electrodes.^208,246^

#### 2.5.3 Quartz Crystal Microbalance with Dissipation (QCM-D)

Quartz Crystal Microbalance with Dissipation monitoring (QCM-D) is highly sensitive to changes in mass and viscoelastic properties of thin films.^356^ A QCM-D instrument functions by applying a sequence of AC pulses to a metal coated piezoelectric quartz crystal while monitoring the frequency at which the crystal resonates and the time constant of the resonance decay between the pulses. Changes in the resonance frequency are inversely proportional to the total mass adsorbed at the crystal, including any solvent or electrolyte molecules coupled to the film, while the energy dissipation is inversely related to film rigidity. Electrochemical QCM-D allows for the i*n situ* monitoring of the electropolymerization process, including monomer interaction with the electrode,^357^ the mass of the growing polymer^358,359^ at the electrode surface, and consequently, the electropolymerization rate.^360^ Other parameters, such as the viscoelastic properties of the growing polymer film,^361,362^ the effectiveness of the incorporation of nonconductive materials during electropolymerization,^363^ film adhesion, and even insights into the reaction mechanism,^364^ can also be extracted.

#### 2.5.4 Spectroscopic Techniques

A wide variety of spectroscopic techniques can be implemented to track the electropolymerization reaction *in situ* using specialized electrochemical cells that incorporate optical windows. The measurement modes assess the absorption, refraction, and scattering of light by the growing film at different regions of the electromagnetic spectrum, providing insights about various aspects of the film at different stages of film growth.

Spectroscopy probing absorption in the ultraviolet and visible (UV–vis) region of the electromagnetic spectrum, generally performed in transmission mode, provides information about the electronic transitions characteristic of the monomer, reaction intermediates, and the polymer during electropolymerization.^365,366^ The spectrum is also sensitive to the doping level of the polymer with distinct regions corresponding to absorbance by the neutral polymer, polaron, and bipolaron states. In situ tracking of the UV–vis absorption of the film can be used to quantify the amount of electrically addressable polymer deposited on the electrode surface that is deconvoluted from 1. the efficiency of the reaction (unlike with integrating the charge consumed during electropolymerization), 2. charge mobility (unlike with conductivity measurements), and 3. the mass of solvent and counterions entrapped in the film (unlike with QCM-D). *In situ* UV–vis can also be used to observe transient intermediates, facilitating the extrapolation of the reaction mechanism.^367–369^ Shifting the configuration of the light path to one that is parallel to the electrode additionally allows for the independent measurement of spectra generated by soluble and insoluble intermediates.^368^

UV–vis spectroscopy can be complemented by FTIR spectroscopy, which probes the vibrational properties of the bonds within the polymer and is likewise insensitive to the aforementioned interferences observed in other techniques.^214,359^ As with UV–vis spectroscopy, the IR spectra of conducting polymers are sensitive to the presence of polarons and bipolarons.^370^ The spectra for PANI^371^ and polythiophene^370^ have been extensively studied and can be used to assign the peaks in the spectra of their derivatives. In situ FTIR measurements have been used to track electropolymerization progress^372^ and evaluate the structure of a copolymer of ANI derivatives.^373^

Raman spectroscopy measures the spectrum of scattered light observed upon the reflection of a laser off of a conducting polymer sample and can be used to probe a different set of vibrations than those observed with FTIR, despite monitoring the same frequency range. Surface-enhanced Raman spectroscopy (SERS) significantly amplifies the Raman scattering signal of molecules adsorbed on rough metal surfaces or nanostructures, making it a powerful tool for probing the very early stages of electropolymerization.^374^ The intensity of the SERS signal, however, is not proportional to the film thickness in a predictable way.^375^ Several insights regarding the changes in film properties throughout the electropolymerization process have been made by analyzing i*n situ* Raman measurements. These insights include observations regarding: 1) the oxidation state of the film as a function of electropolymerization time,^376^ 2) the presence of monomer species at early stages of electropolymerization,^377^ and 3) the degree of film polymerization.^378^

The complex dielectric function of the electropolymerized material can be probed *in situ* by several techniques, including spectroscopic ellipsometry (SE), reflectometry, and surface plasmon resonance spectroscopy (SPR). Spectroscopic ellipsometry evaluates the change in the polarization state of light as it interacts with a growing OMIEC film by either transmission or reflection. Careful layer-by-layer construction of a model describing the potential-dependent dielectric function, defined as the square of the complex refractive index, of both the substrate and the film can assist in evaluating the film growth. Spectroscopic ellipsometry was first used to characterize the electrochemical deposition of a PPy film under potentiostatic conditions.^379^ Interpretation of the results revealed distinct phases of film growth—a highly hydrated film with fibers extending away from the substrate up to a thickness of 15 nm, the coalescence of the fibrous structures at a constant film thickness, and the linear increase of film thickness with increasing density. Correia et al.^380^ determined that a PPy film with a titanocene dichloride side chain, deposited by CV, grows linearly as a function of cycle number and has a uniform optical density throughout the film. They observed, however, that there are differences in the extinction coefficients between the inner and outer regions of the film during electropolymerization, which they attribute to the presence of soluble oligomers entrapped in the pores of the film at the outer surface.

Optical fixed angle reflectometry is a simplified version of spectroscopic ellipsometry, which does not provide information regarding the phase shift of the light as it interacts with matter. For this reason, more assumptions must be made when extracting the refractive index. It can prove useful, however, for qualitative *in situ* evaluation of experimental conditions on film growth. For example, Monnin et al. showed that introducing a flow of the monomer solution during electropolymerization surprisingly inhibited the formation of a well-adhered film of bithiophene at the electrode.^381^

Surface plasmon resonance (SPR) measures the characteristics of the charge density oscillations (surface plasmons) in a metal’s free electrons at the metal-dielectric interface. Photons with the appropriate energy and polarization induce these oscillations, generating an evanescent field that extends into the dielectric. This wave is highly sensitive to changes in the refractive index at the surface and is most simply observed as an absorbance of monochromatic light at a given angle of incidence. Due to its high sensitivity to the adsorption of organic materials in the vicinity of the gold surface, SPR is often used as a sensor transduction mechanism for analyte binding. The amplitude of the surface plasmon decays exponentially as it penetrates into the dielectric, which imposes a limit on the sensitivity of standard SPR to approximately 200 nm and of long-range SPR to approximately a micron of the electrode surface.^382,383^ Electrochemical SPR was used to monitor the electrochemical deposition rates of PPy and a PPy-glyphosphate molecularly imprinted polymer (MIP) as well as the adsorption of both PPy and glyphosphate to the gold electrode.^384^ It was found that the reduced rate of MIP deposition is a result of the insulating properties of the adsorbed layer of glyphosphate, which binds with a much higher affinity to gold than PPy. Coupled techniques that provide a parallel analysis of OMIEC properties during electropolymerization, like QCM-D, can be used to great effect to deconvolute the contribution of doping-related changes in the refractive index and the desorption of mass from the surface.

#### 2.5.5 Scanning Probe Techniques

The surface morphology and out-of-plane conductivity of an OMIEC film can also be investigated in situ during electropolymerization by electrochemical scanning probe techniques like atomic force microscopy (AFM) and scanning tunneling microscopy (STM). Electrochemical atomic force microscopy (EC-AFM) is a high-resolution scanning probe method that can be adapted to map the topography, conductivity, and mechanical properties of a surface. AFM functions by measuring the interaction between a nm-scale probe and the surface on which electropolymerization occurs, as the probe is rastered back and forth across the sample. EC-AFM can be performed in either the passive configuration, where the probe is unbiased and maps the topography of the sample, or the active configuration, where the probe is biased, which allows for the mapping of the current flowing between the probe and the underlying electrode.^385^ The nucleation stage of potentiostatic ANI^385^ and bithiophene^238^ electropolymerization was studied by conductive AFM in the passive configuration, demonstrating the growth of nm-scale deposits on the electrode surface. Both potentiostatic and potentiodynamic deposition of PEDOT films were investigated in real-time by tandem passive AFM and SPR. The authors reported an abrupt increase in the surface roughness at potentials between 0.9 and 1.2 V vs Ag wire and a clear difference in the deposition mechanism between potentiostatic and potentiodynamic deposition techniques.^386^ Copolymerization of PPy and horseradish peroxidase (HRP) enzyme was also studied in situ, but periodically, with images acquired between electropolymerization steps, showing unique morphologies for PPy and PPy-embedded HRP.

Unlike AFM, which relies on the physical deflection of the tip, STM relies on the tunneling current to maintain a constant distance from the surface during rastering. Consequently, the resolution is not constrained by the physical dimensions of the probe, allowing for molecular-level resolution on highly crystalline substrates. The adsorption of terthiophene,^387^ EDOT,^388^ and isomers of ethyl aniline^389^ on Au(111) substrates, as well as the structure of the resulting polymers, have been imaged by STM at the molecular scale. The measurements allow for the determination of monomer alignment with the crystal lattice of the substrate, the extent of polymer branching as a function of potential,^389^ and the level of disorder observed in the polymer chains.

## 3 Electropolymerization in Bioelectronics

### 3.1 Biological Interfaces

Conducting polymers have gained significant attention in the field of biological and neural interfaces due to the unique combination of mixed ionic and electrical conductivity, along with their flexibility, and biocompatibility. Unlike traditional metal-based materials, conducting polymers such as PPy and PEDOT offer a softer, more adaptable interface with biological tissues. As a result of the mixed ionic-electronic conductivity, these materials are capable of efficiently transducing ionic signals to electronic ones, or vice versa, making them ideal for applications like neural stimulation and recording.^390^ The performance improvement for these applications manifests itself in higher signal-to-noise ratios for recordings or, in the case of neural modulation, reduced necessary voltages for a given stimulation current pulse or increased charge injection capacity.^391,392^ These improvements can be attributed to a reduction in the electrochemical impedance of a specific biological interfacing site when utilizing conducting polymer films or coatings. The improved electrochemical impedance in turn allows for miniaturization of electrode sites while maintaining good performance. The small size of neural interfacing site is needed to allow precise recording capability (i.e., single cell/action potential) as well as precise control over the tissue affected by neural stimulation. In addition to these benefits, the ability of conducting polymers to be chemically modified and tailored for specific functionalities, such as drug delivery or promoting cell growth, further enhances their potential in neural interfaces, particularly in treating neurological disorders and developing brain-machine interfaces.

When practically creating conducting polymer films for biological interfacing applications, electrochemical polymerization offers a variety of advantages compared to chemical oxidative or vapor phase polymerization. This process allows the growth of the polymer precisely on the specific area of interest, bypassing additional patterning steps and allowing many electrodes to be modified in parallel. In addition, by adjusting the electropolymerization conditions, such as the method (i.e., galvanostatic, potentiostatic, cyclic voltammetry, etc.), current/voltage used, counterion(s), and electrolyte composition, the morphology and thickness can be controlled. The possibility to incorporate growth factors or various drugs in a straightforward manner during the deposition process is also of great interest.^393^ Finally, a significant benefit of electropolymerization is that it enables polymer growth on or in complex structures, including electrodes or conductive substrates of any shape or size. This level of customization is particularly advantageous in neural interfaces, where specific functionalities like localized drug release or tissue integration are crucial. By fine-tuning the polymerization process, electrochemical polymerization can create coatings that improve biocompatibility, enhance electrical conductivity, and support nerve tissue repair, making it especially useful in treating neurological disorders and advancing biological/neural interfacing technology.

Many of the early investigations into the use of conducting polymers for biological interfaces involved PPy. When focusing specifically on neural interfaces, a great deal of the seminal work exploring the use of conducting polymers was led by David C. Martin and colleagues. The first example to study nerve cell interactions with conducting polymers, however, was Schmidt et al. in 1997, with the aim of using such systems to aid in nerve regeneration.^397^ PPy:PSS was demonstrated to support PC-12 cell cultures and explanted primary chicken sciatic nerves equally well to typical polystyrene dishes for tissue culture in the absence of stimulation. When employing stimulation protocols through the PPy films, significantly increased neurite outgrowth length was observed in comparison to the control samples, a promising result for the aspired nerve regeneration application. Initial investigations of conductive polymer coatings on neural electrodes also employed PPy, exploring the use of various counterions to include biomolecules intended to attract cells.^394^ Cui et al. used the synthetic protein polymer SLPF and laminin fragment CDPGYIGSR, incorporated in the PPy films precisely deposited on the electrode sites of silicon neural probes through electropolymerization (Figure 13a). A reduction in the electrochemical impedance was observed compared to uncoated sites, along with high-quality *in vivo* recordings from the guinea pig cerebellum. Additionally, *in vitro* studies showed selective cell growth on PPy/CDPGYIGSR-coated sites compared to PPy/CH3COO^–^.

**Figure 13:**
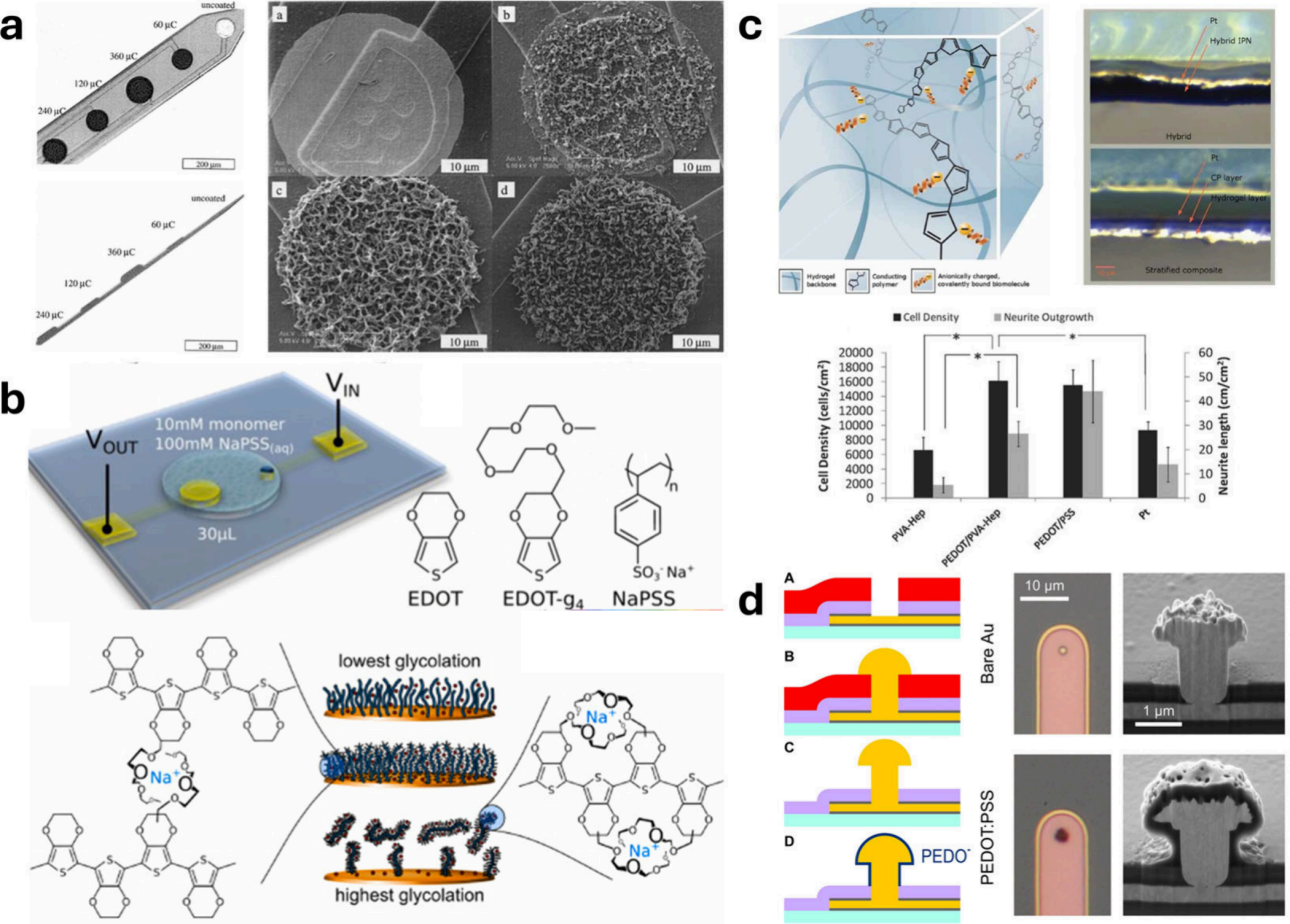
a) Deposition of PPy/SLPF on neural probe electrode sites with varying deposition charges. The fuzzy structure (right panels), dependent on the deposition parameters, increases the surface area, while the incorporation of biomolecules encourages interaction with neurons.^394^ b) By controlling the glycolation content of MEA coatings, cell-electrode interfacing and signal-to-noise performance are optimized.^75^ c) Conductive hydrogel interpenetrating networks are used to coat platinum electrodes, providing superior mechanical stability and a Young’s modulus significantly closer to that of neural tissue and increasing the charge storage capacity and charge injection capacity.^395^ Using the PC12 cell line, a similar cell density was seen when compared to PEDOT:PSS (lower panel), but lower neurite outgrowth, presumably due to the cell interaction with the substrate. d) Electropolymerization enables coating complex structures such as the Au mushrooms with diameters down to 1 μm shown here. The resulting ultramicroelectrodes are capable of stable recordings over 4 weeks in vitro during development of ex vivo neuronal networks.^396^ Panels a and c reproduced with permission from references 394 and 395 respectively, copyright John Wiley & Sons, Inc., 2001 and 2012. Panel b reproduced with permission from reference 75, Copyright Elsevier B.V., 2023. Panel d reproduced from reference 396 (CC BY).

Although PPy-based work was encouraging for the use of conducting polymers at neural interfaces, this material often proved disadvantageous. Negative results include poor rate capability for neural stimulation, with relatively long current pulses required to achieve reasonable charge densities during controlled current pulsing as well as delamination from the substrate when higher charge densities were utilized.^391,398^ PEDOT has been explored as an alternative, and since the early 2000s, it has received the bulk of attention for bioelectronic applications in general, including work to improve neural recording and stimulation capability. A comparison study was carried out by Cui & Martin in 2003, using coatings of PEDOT:PSS and demonstrating greatly improved electrochemical stability.^399^ PEDOT coatings incorporating the bioactive peptide DCDPGYIGSR were also used in this work, showing preferential growth of rat glial cells in vitro and high-quality recording capability in vivo.

A variety of studies have subsequently investigated the use of copolymerization, counterion variation, and additive inclusion or have made specialized use of electrochemical deposition to create layered or interpenetrating networks. A recent example comparing p­(EDOT*-ran*-EDOTOH) polymerized with different counterions (ClO4 or PSS) and in the presence of a variety of additives (EG, EMIM PF6, EMIM TFSI, MW CNTs, MXene, PEDOT Nys, Tetra EG) was carried out by Nikiforidis et al.^393^ The process variant using copolymer p­(EDOT-*ran*-EDOTOH) with ClO_4_ dopant and EG as an additive proved to be most optimal for subsequent in vitro study, based on the specific and double layer capacitance values, as well as the electrochemical stability. Good recording and stimulation performance was observed during experiments using mouse primary cortical neurons cultured on polymer-coated MEAs compared to uncoated electrodes. The use of copolymerization was also explored by Ghazal et al., depositing EDOT with its triglymated derivative in the presence of NaPSS on top of gold MEAs (Figure 13b).^75^ Optimal monomer ratios were sought to find the best glycolation degree and, when mouse primary cortical neurons were cultured on the electrodes, a better SNR was seen as well as a higher number of spikes detected by the glycolated PEDOT microelectrodes compared to PEDOT alone and TiN commercial MEAs.

Green and co-workers explored the use of electropolymerization to deposit PEDOT through conductive hydrogels (CHs), forming hybrid interpenetrating networks and often applied to commercial cochlear implant electrodes.^41,395,400,401^ The performance of the explored material system is mainly demonstrated through CSC retention after accelerated aging tests, while in some cases neurite outgrowth is also investigated. The hybrid interpenetrating networks were created by first electrochemically polymerizing PEDOT doped with *para*-toluenesulfonate (PEDOT/pTS) at a Pt surface, followed by photopolymerization of a thin film of 18 wt % poly­(vinyl alcohol) (PVA)/2 wt % heparin methacrylate (Hep-MA), then last electrodepositing the PEDOT throughout the hydrogel network (Figure 13c).^395^ This approach showed improved mechanics for interfacing with neural tissue as well as superior electroactivity retention when compared to uncoated metal electrodes. Hassarati et al. made use of this approach to coat cochlear implant electrodes, resulting in higher charge storage capacity (>2x increase) and charge injection capacity, as well as good stability during lifetime testing with up to 2 billion stimulation pulses.^41^ Building on the layered HG approach, “living electrodes” with PC12 in the outer layer were also proposed to create soft, cell integrated coatings to address the problem of scar tissue encapsulation of stimulating neuroprosthetics.^400^ This approach was demonstrated to successfully embed neural cells and to both support cell growth with some neurite outgrowth observed, and to maintain or improve the electroactivity of the electrodes. More recently, Chik et al. made use of electrodeposited hydrogel coatings poly­(2-hydroxyethyl methacrylate) (pHEMA) with PEDOT:PSS to provide low impedance and bridge the mechanical mismatch between the flexible electronics and soft neural tissue. The performance of both recording and stimulation are highlighted when implanted in the rat hippocampus.^402^

Electrochemical deposition on complex physical structures has also proven beneficial, such as the work of Jones et al. in 2020 through the fabrication of in vitro arrays of gold mushroom ultramicroelectrodes with diameters down to 1 μm. Electrochemical deposition enabled PEDOT:PSS coatings on these irregular surface structures, reducing the electrochemical impedance of the microelectrodes and significantly improving the quality of extracellular recordings (Figure 13d).^396^ Previous work by Yang et al. and Abidian et al. also made use of polymerization on or around highly structured surfaces, however, using the structures to template the conducting polymer layers.^403–405^ The former work used 300 nm PS latex spheres as a template, deposited on the neural probe surface.^403^ Afterward, PEDOT or PPy doped using LiClO_4_ were electrodeposited onto the electrode sites through and around the template, and the PS spheres were then removed, leaving behind a highly microporous 3D structure with low impedance. In 2006, Abidian et al. employed PLGA and PLGA/dexamethasone as a template for PEDOT and PPy on Au neural probe electrodes.^405^

Electropolymerization has even been taken advantage of to explore polymerization in living systems such as plants and parts of the nervous system. Recently, Pham et al. explored the use of electropolymerization in the vascular system of plant leaves, investigating the possibility to embed biofuel cell electrode materials.^406^ Thiophene (T) and ethylenedioxythiophene (E)-based trimers (ETE) anchoring an Os­(2,2′-bipyridine)­2­(1-(3-aminopropyl)-imidazole)­Cl Os-complex were injected into the plant’s vascular system (Figure 14a). Subsequently, electropolymerization is carried out, demonstrating the possibility for in vivo supported electrocatalysts through polymerization of ETE-Os molecules with different standard potentials at specific locations inside the vascular system. Already in 2007 Richardso*n*-Burns et al. carried out work to explore cell templated polymer deposition and in vivo polymerization in living neural tissue (Figure 14b).^17,18^ The in vitro test portion of these studies showed high cell viability (neuroblastoma-derived cells) for 0.001 M EDOT when polymerization was carried out around the cells. To create a cell-templated, biomimetic surface structure, the cells were subsequently removed by exposure to trypsin–versene. Promising results were observed in vitro, showing that cells of the host tissue may be encouraged to repopulate the cell-shaped surface structure, including utilization of the tunnels and crevasses created by templating around neurites and cellular processes. When polymerizing directly in mouse brain tissue, the possibility to grow fibrils within small spaces between cells and extending up to 0.5 – 1 mm from the electrode surface was shown. This result holds potential for creating an intimate contact between the conducting polymer and neural tissue, as well as the possibility to bypass the typical scar encapsulation that results from the body’s foreign body response to implanted devices. Hjort et al. have recently demonstrated polymerization of bioresorbable poly­(3,4-ethylenedioxythiophene)­butoxy-1-sulfonate (PEDOT-S) derivatives directly in the zebrafish brain. The self-doped, water-soluble PEDOT-S derivative A5 was employed along with ETE-S and ETE-PC to improve conductivity (Figure 14c).^16^ These materials were injected using a small diameter capillary into the zebrafish brain, self-organizing into a mixed ion–electron conducting hydrogel. The transient nature of the formed conductive hydrogels was demonstrated in zebrafish allowed to swim for weeks after the injection and polymerization. Additionally, the inflammatory response observed 2 h after injection was shown to be resolved after 9 days of free swimming.

**Figure 14:**
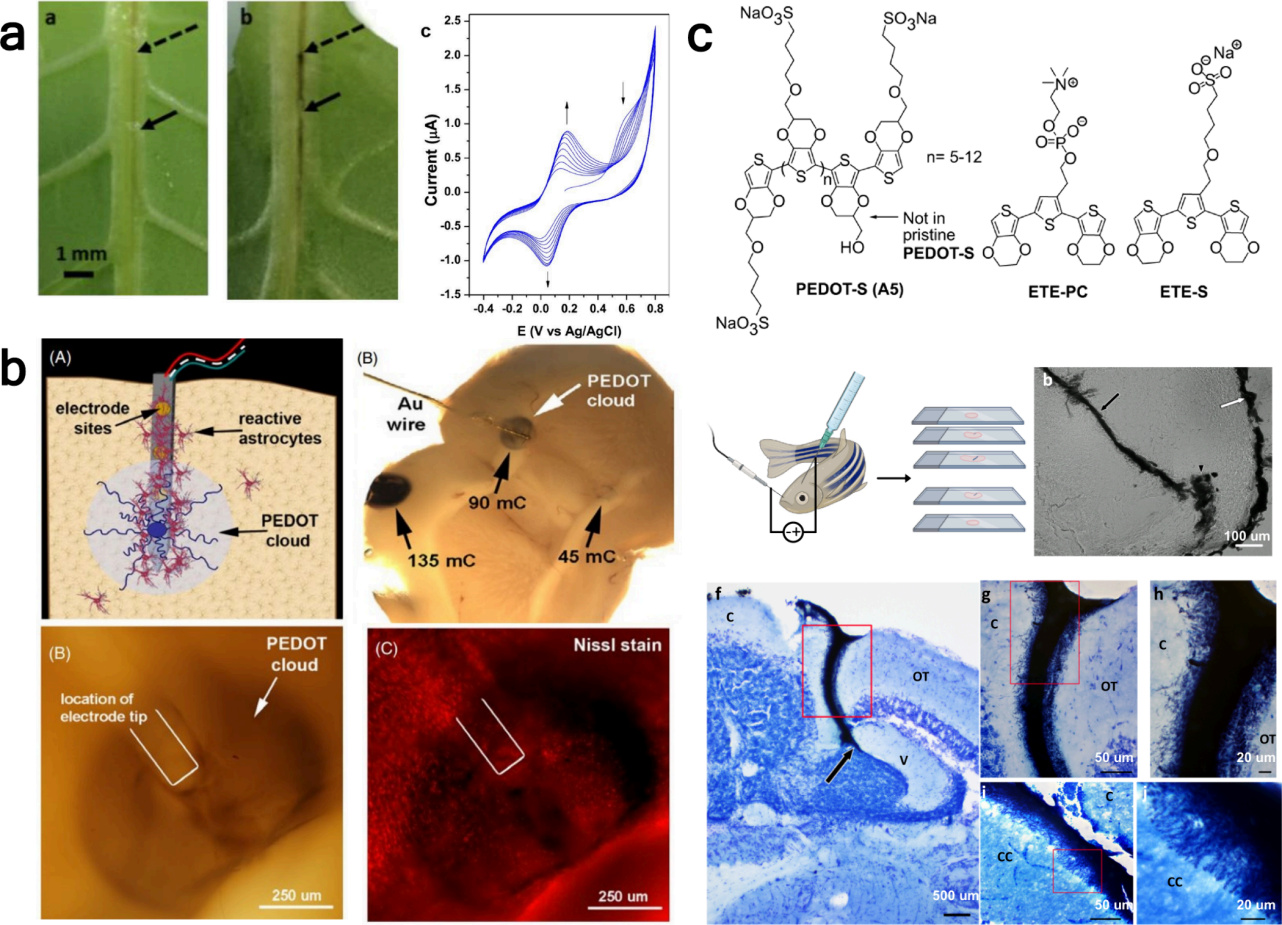
a) Electropolymerization to probe the living plant vascular system is demonstrated through the use of thiophene (T) and ethylenedioxythiophene (E)-based trimers (ETE) anchoring an Os­(2,2′-bipyridine)-2-(1-(3-aminopropyl)-imidazole)­Cl Os-complex.^406^ b) PEDOT polymerized directly in the mouse brain tissue provides the possibility to bypass the glial scar (depicted in the upper left panel). The resulting polymer formation is shown for different deposition charges (upper right) and the PEDOT fibril formation, dependent on tissue morphology (lower left), and intact neurons after polymerization (lower right) are shown.^17^ c) Bioresorbable electronics are fabricated in the zebrafish brain using a PEDOT-S derivative A5 (chemical structure shown in upper panel). A microinjection process was carried out and brain slices were analyzed after the fish were allowed to swim for 2 h or 9 days. The formed polymer follows the injection track for short polymerization durations (1 min, middle right and lower panels), while longer polymerization times result in dendritic outgrowth (5 min, not shown).^16^ Panel a reproduced with permission from reference 406 Copyright Elsevier B.V., 2025. Panel b reproduced with permission from reference 17, Copyright IOP Publishing, 2007. Panel c reproduced from reference 16 (CC BY 4.0).

The overall possibilities in the field of neural interfaces through the use of electropolymerization methods are promising. From improvement of recording or stimulation electrode performance through coating optimization to direct polymerization in vivo, exciting opportunities to intimately interface with neural tissue exist.

### 3.2 Sensors and Biosensors

Sensors detect and respond to physical or chemical properties of interest, converting them into readable signals for measurement and analysis. Biosensors additionally leverage the high specificity of natural binding interactions by incorporating biological recognition elements, such as enzymes, nucleic acids, or antibodies, to detect and quantify the desired analyte. OMIECs are commonly used to fabricate the environmentally sensitive layer for sensors and biosensors that translate the interaction of the sensing layer with the analyte to an electrical signal. Electropolymerization is a highly versatile technique for fabricating OMIEC devices for sensor applications, particularly biosensors, due to several key advantages. The fabrication process is straightforward, and it ensures excellent reproducibility when process conditions are maintained.^407^ This method also offers significant tunability, allowing for control over factors such as counterions, additives, and deposition parameters.^408^ Moreover, electropolymerization is adaptable to any conductive surface, providing precise control over the amount of material deposited, which occurs exclusively at the electroactive surface. The particular suitability for biosensing application stems from the ability to tune the deposition parameters to facilitate biocompatibility and the possibility to immobilize biomolecules. Incorporating biomolecules into biosensors enhances selectivity and sensitivity by enabling the recognition of specific biological targets through natural binding interactions. All features together make electropolymerization a valuable tool in the development of reliable and customizable sensors. Sensors that incorporate OMIEC electropolymerization in the fabrication involve a huge variety of transduction mechanisms and recognition elements have been reported. These sensors are described in detail in this section and summarized in Table 2.

**Table 2:** Summary of the Sensors Described in Section 3 of This Review^a^

First author and reference	Electrode	Analyte	Immobilization/approach	OMIEC material	Recognition element	LOD	Response time	Electrode size	Linear Range
Nicolini^409^	OECT channel	Zn^2+^	Monomer modfication	pTri-DPA (2-(bis(pyridin-2-ylmethyl)amino)ethyl 2-(2,5-bis(3,4-ethylenedioxy thiophene)thiophen-3-yl)acetate)	Ligand–receptor (DPA)	1.5 μM	∼few minutes**	100 μm x 10 μm = 0.001 mm^2^	1 μM to 1 mM
Wustoni^411^	OECT gate	Na^+^, K^+^	Monomer modfication	Crown ether functionalized thiophene (3,4-(15-crown-5)thiophene (T15c5) for Na^+^, and 3,4-(18-crown-6)thiophene (T18c6)) for K+	Ligand–receptor (Crown ether)	20 μM (Na+), 0.1 mM (K+),	<10 s	500 μm × 500 μm = 0.25 cm^2^	10 μM - 1 M, 0.1 mM - 1 M
Mariani^410^	OECT gate	pH	pH dye-doped PEDOT	Bromothymol Blue doped PEDOT	Ligand–receptor (pH dye)	NS	∼few minutes**	5 mm × 5 mm = 0.25 mm^2^	10^–2^ to 10^–7^ M (i.e., pH 2–7), (110 mV/pH)
Sulka^416^	Anodic aluminum oxide membrane (80 nm pores)	pH	HQS-doped PPy	PPy-HQS NWs (Hydroquinone monosulfonate)	Ligand–receptor (redox-active)	NS	<10 s	Area NS, NWs: 80 nm × ∼ 17.6 μm	10^–2^ to 10^–12^ M (i.e., pH 2–12), ∼ 49 mV/pH
Satyanarayana^419^	Carbon nanotube paste	Dacarbazine	Covalent polymer attachment to CNT	Poly(2-amino-1,3,4-thiadiazole)	None (electrocatalytic oxidation)	35 nM	<3 s	Ø 2 mm = 12.56 mm^2^	50 nM – 24 μM
Farshadinia^420^	Carbon paste electrode	Amoxicillin	Adsorption of copolymer clusters	Poly(diphenylamine-*co*-4,4′-diaminodiphenyl ether)	None (electrocatalytic oxidation)	1 μM	NS	Ø = 2.6 mm = 5.3 mm^2^	20 μM – 0.4 mM
Castagnola^421^	CNT-coated glassy carbon microelectrode	Serotonin, 5-HT	PEDOT/CNT nanocomposite	PEDOT/CNT nanocomposite	None (electrocatalytic oxidation)	<100 nM*	∼15 s* (scan time - SWV measurement)	Ø 40 μm = 1256 μm^2^	100 nM - 1 μM
Foulds^424^	Pt ink	Glucose	Enzyme entrapment	PPy	Enzyme (glucose oxidase (GOx))	NS	20–40 s	0.16 cm^2^	∼0–25 mM**
Umana^425^	GC, Pt electrodes	Glucose	Enzyme entrapment	PPy	Enzyme (GOx)	NS	∼minutes**	GC: 0.08 cm^2^, Pt: 0.2 cm^2^	0.1–10 mM**
Shinohara^429^	Platinum fiber	Glucose	Enzyme entrapment	PANI	Enzyme (GOx)	NS	20–40 s	Ø 50 μm = 0.00196 mm^2^	0.1–5 mM
Yang^430^	Pt microelectrode	Glucose	Enzyme entrapment	PEDOT nanofibers	Enzyme (GOx)	0.26 mM (at +300 mV), 0.12 mM* (at +700 mV)	<10 s**	1394 μm^2^	0.1–5 mM
Chen^431^	Carbon fiber microelectrode	Glucose	Electrostatic enzyme adsorption	PEDOT:ClO4	Enzyme (GOx)	NS	<2 s	Ø 7 μm = 38.5 μm^2^	0.5–15 mM
Kuwahara^432^	Gold electrode	Glucose	Enzyme and redox mediator covalent immobilization	3-methylthiophene and thiophene-3-acetic acid copolymer (3MT/T3A)	Enzyme (GOx)	NS	NS	0.25 cm^2^	up to 2.5 mM**
Invernale^433^	Pt-coated stainless steel microneedle	Glucose	Enzyme entrapment	PEDOT/pTS	Enzyme (GOx)	NS	NS	680 μm long × 250 μm wide	2–24 mM
Patil^434^	Mild steel	Glucose	Physical adsorption	Poly(o-anisidine) (POA)	Enzyme (GOx)	2 mM	5 s	10 mm × 15 mm = 1.5 cm^2^	2–20 mM
Singh^435^	ITO glass	Cholesterol oleate	Covalent enzyme attachment (via glutaraldehyde)	PANI	Enzymes (cholesterol esterase + cholesterol oxidase (COD))	<1.3 mM*	∼40 s	NS	50–500 mg/dL (1.3–13 mM)
Emre^436^	Graphite rod	Glucose	Physical adsorption + glutaraldehyde	Poly(4,7-di(2,3)-dihydrothienol[3,4-b][1,4]dioxin-5-yl-benzo[1,2,5]thiadiazole) (PBDT) and poly(4,7- di(2,3)-dihydrothienol[3,4-b][1,4]dioxin-5-yl-2,1,3-benzoselenadiazole) (PESeE)	Enzyme (GOx)	0.05 mM (PBDT), 0.01 mM (PESeE)	NS	Ø 3.05 mm = 7.3 mm^2^	0.05 – 2.0 mM (PBDT), 0.01 – 2.0 mM (PESeE)
Tamer^437^	Pt	Glucose	Covalent enzyme attachment (via glutaraldehyde)	PANI:AuNR (PANI with gold nanorods)	Enzyme (GOx)	5.8 μM	<3 s	0.022 cm^2^	17.6 μM – 1 mM
Tan^438^	Pencil graphite electrode	Glucose	Covalent enzyme attachment (via glutaraldehyde)	p(EDOTBN):AuNPs	Enzyme (GOx)	8.5 μM	NS	Ø 0.5 mm = 0.196 mm^2^	11.7 μM–10 mM
Trettnak^439^	Pt disk	Cholesterol	Enzyme entrapment	PPy (overoxidized)	Enzyme (cholesterol oxidase)	∼5 μM*	∼1–3 min*	Ø 1.75 mm = 0.024 cm^2^	0.07–0.25 mM**
Rahman^440^	Pt microelectrode	Glutamate	Covalent enzyme attachment (via EDC) (GlOx + AsOx)	Poly(terthiophene-3-carboxylic acid):TBAP	Enzyme (glutamate oxidase and ascorbate oxidase)	0.1 μM	<10 s	Ø 25 μm = 490.9 μm^2^	0.2 – 100 μM
Vidal^441^	Pt disc	Cholesterol	Enzyme entrapment	Overoxidized PPy:KCl	Enzyme (cholesterol oxidase)	5.7 μM	7.5 s	Ø 3 mm = 0.071 cm^2^	25 – 300 μM
Rahman^442^	Glassy carbon	Choline	Covalent enzyme (co)attachment (via EDC)	Poly(terthiophene-3-carboxylic acid):TBAP	Enzyme (choline oxidase (ChO))	0.4 uM (ChO), 0.1 uM (ChO + HRP)	<5 s	0.07 cm^2^	1–50 μM (ChO), 1–80 μM (ChO+HRP)
Braik^443^	Glassy carbon	Superoxide	Covalent enzyme attachment (via glutaraldehyde)	PEDOT:PSS/CNT + chitosan	Enzyme (superoxide dismutase)	1 uM	<10 s**	NS	20–3000 μM
Nguyen^444^	Pt microelectrode	Lactose	Covalent enzyme attachment (via glutaraldehyde with BSA)	Graphene/poly(1,5-diaminonaphthalene)	Enzymes (β-Galactosidase + GOx)	3.8 μM	NS	Ø 200 μm = 0.031 mm^2^	∼3.8 – 175 μM
Ho^445^	ITO	Morphine	Entrapped MIP particles	PEDOT:ClO_4_ + MIP	Molecularly imprinted polymer	0.3 mM	NS	1 cm × 0.5 cm = 50 mm^2^	0.1–2.0 mM
Fenoy^447^	Au IDEs (OECT channel)	Thrombin	Covalent coupling via SPAAC click chemistry	PEDOT-N_3_ (azido-EDOT)	Aptamer (HD22-DBCO)	31 nM	∼1 min**	NS	∼25–100 nM**
Alexander^448^	Glassy carbon	Glucose	Dropcast GO-MIP in Nafion binder	GO-MIP composite (GMA/MAA/EGDMA)	Molecularly imprinted polymer	0.1 nM	∼2 min	Ø 3 mm = 0.071 cm^2^	0.01–6.0 mM*
Cui^455^	GCE	Alpha fetoprotein (AFP)	Antibody adsorption (via AuNPs)	PEDOT:PEG + AuNPs	Antibody (anti-AFP)	0.0003 fg/mL	∼30 min*	Ø 3 mm = 0.071 cm^2^	0.001–10 fg/mL
Zidaric^456^	SPCE	Insulin	Template entrapment	PPy	Molecularly imprinted polymer	1.9 pM	∼15 min*	Ø 1 mm = 0.00785 cm^2^	20–70 pM
Wustoni^446^	Au (OECT channel)	Glucose, cholesterol, lactate	Enzyme entrapment	p(EDOT-*ran*-EDOTOH):ClO_4_	Enzyme (GOx, cholesterol oxidase, lactate oxidase)	NS	NS	100 × 10 μm^2^	0.1–1 mM (first of 2 linear ranges for all sensors)
Fenoy^460^	rGO IDEs (gFET channel)	Glucose	Electrostatic enzyme immobilization	rGO/poly(ABA-*co*-aniline) (PABA)	Enzyme (GOx)	4.1 μM	∼190 s	10 μm × 240 mm = 2.4 mm^2^	10 μM–1 mM
Hai^462^	IDEs (OECT channel, material NS)	Influenza A virus	Covalent ligand attachment (via oxime)	poly(EDOTOA-*co*-EDOT)/PEDOT:PSS	Ligand–receptor (2,6-sialyllactose)	0.025 HAU	∼10 min *	25,800 μm x 5 μm = 0.0013 cm^2^	0.03–1 HAU
Janardhanan^463^	NS (OECT channel)	Cortisol	Covalent antibody attachment (via EDC/Sulfo-NHS)	poly(EDOT-COOH-*co*-EDOT-EG3) NTs	Antibody (anticortisol)	0.0088 fg/mL	<10 s	NS	1 fg/mL–1 μg/mL
Tao^464^	Carbon fiber (OECT gate)	Uric acid	Template entrapment on gate	PEDOT:ClO_4_/MIP (polydopamine)	Molecularly imprinted polymer (template: uric acid)	NS	∼minute**	NS	1 nM–500 μM
Tang^465^	Pt (OECT gate)	Dopamine	Template entrapment on gate	PPy (overoxidized)	Molecularly imprinted polymer (template: dopamine)	34 nM	∼100 s	1 cm^2^	1–10 μM**
Fenoy^461^	rGO IDEs (gFET channel)	Acetylcholine	Electrostatic enzyme immobilization	rGO/poly(ABA-*co*-aniline) (PABA)	Enzyme (AchE)	2.3 μM	∼130 s	10 μm × 240 mm = 2.4 mm^2^	5 μM–1 mM

a NS: Not specified/*: implied/**: estimated from figures.

#### 3.2.1 Sensing Based on Intrinsic OMIEC Properties

##### 3.2.1.1 Ion Selectivity

Some recent studies have investigated synthesis of monomers bearing cation-selective chelators for a membrane-free approach to the associated ion-sensitivity and selectivity.^409,411–413^ For example, crown-ether-functionalized thiophene units were made to be specific to either Na^+^ or K^+^ and incorporated into an OECT platform.^411^ When electropolymerized on the gate and used in combination with a PEDOT:PSS channel, an ion-sensitive and selective modulation in *I*_*D*_ was observed and the sensors could be employed for detection in human serum. Nicolini et al. employed a similar method to develop a Zn^2+^ OECT sensor, based on chemical modification of a thiophene trimer (2,5-bis­(3,4-ethylenedioxy thiophene)­thiophene) (Figure 15a).^409^ Dipicolylamine (DPA) was included on the trimer, resulting in 2-(bis­(pyridin-2-ylmethyl)­amino)­ethyl 2-(2,5-bis­(3,4-ethylenedioxy thiophene)­thiophen-3-yl)­acetate or ‘Tri-DPA’. In this case, pTri-DPA was deposited in the OECT channel area with Zn^2+^ present as a template to avoid degradation of the DPA moiety, observed in the case of electropolymerization of the noncomplexed molecules. The favorable effect of using the Zn^2+^ template was shown through optoelectronic, electrochemical, and electrical characterization. Real time OECT measurements were carried out with additions of Zn^2+^, Mg^2+^, Ca^2+^, and K^+^ showing little or no response to interferent ions (typically found in biological media), while a significant change in drain current, *ΔI*_*D*_, was observed for Zn^2+^.

**Figure 15:**
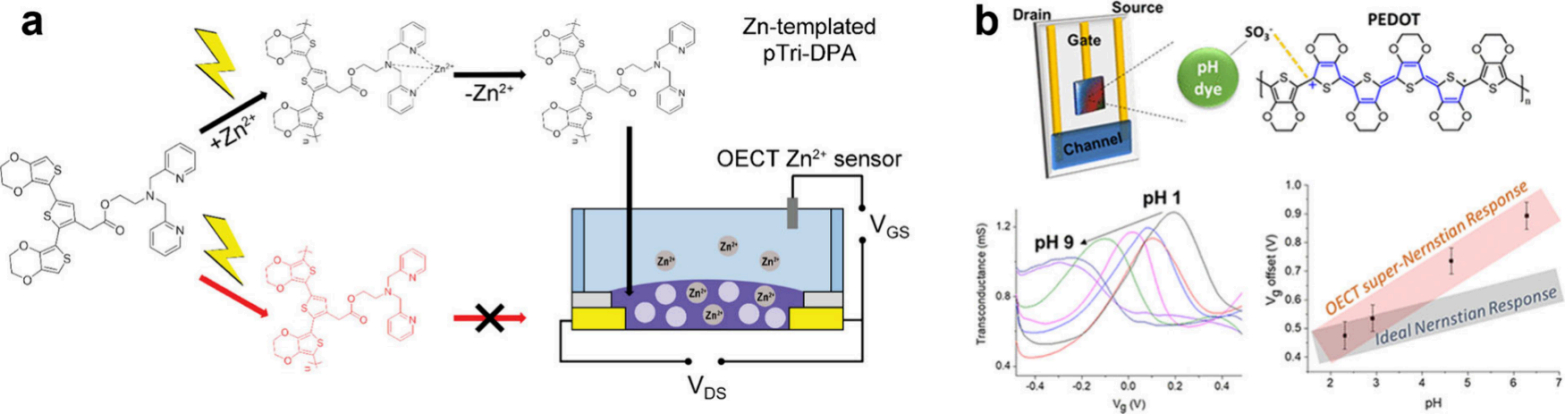
a) Selective detection of Zn^2+^ is achieved using an electropolymerized thiophene-based trimer chemically modified with a dipicolylamine substituent and using a Zn^2+^ template. Incorporation in an OECT platform was possible, showing good selectivity over other biologically relevant cations.^409^ b) Super-Nernstian pH sensitivity is demonstrated utilizing electropolymerized films of pH-sensitive PEDOT composites. pH dyes Bromothymol Blue and Methyl Orange are present as dopants during deposition of the OECT gate coating, successfully providing the capability to convert the pH signal into an electrical one.^410^ Panel a reproduced from reference 409 (CC BY), panel b reproduced from reference 410 Copyright American Chemical Society, 2024.

##### 3.2.1.2 pH Response

pH sensing is important in biological, environmental, and industrial processes. When constructing pH sensors, the materials play a crucial role, determining the sensitivity, stability, and selectivity for detecting the concentration of hydrogen ions. pH-sensitive OMIEC materials have been used as both electrode coatings and incorporated into OECT devices to fabricate pH sensors. Nanostructuring or nanomaterials are often explored to increase surface area and enhance sensitivity. For example, Yun et al. explored the electrochemical growth of different types of nanowires, including PPy wires.^414^ Growth is confined within a patterned SiO_2_ channel between two metal contacts. pH sensitivity was shown in an amperometric measurement using the 500 nm wide, 3 μm long PPy nanowire. Ramanathan further developed this approach in a more detailed report on the galvanostatic growth of well-defined PPy and PANI nanowires.^415^ Individually addressable wires with width/length ratios of up to 100 nm wide/13 μm long were grown were reported in addition to larger resistance changes for a given pH change when using PANI compared to PPy. Sulka et al. reported highly responsive pH sensors based on hydroquinone monosulfonate-doped PPy nanowires fabricated by anodic electropolymerization through a porous aluminum oxide template.^416^

OECT use in pH sensing has proven beneficial, resulting in examples of super Nernstian response. Mariani et al. investigated the use of pH dyes Bromothymol Blue (BTB) and Methyl orange (MO) as dopants for PEDOT.^410^ Electrochemical deposition of PEDOT and the respective dye was carried out on the OECT gate, with PEDOT:BTB resulting in the best performance (Figure 15b). When comparing the material performance, PEDOT:BTB exhibited a sensitivity of 62 ± 2 mV pH unit^–1^ n when use in the OECT platform the sensitivity was 93 ± 8 mV pH unit^–1^ over the pH range of 1–9. Lastly, to demonstrate the potential for bioelectronic application, the sensor was fabricated on a flexible polyethylene terephthalate (PET) substrate. Demuru et al. also made use of the OECT platform to demonstrate super-Nernstian pH sensitivity.^417^ In this case, a PEDOT:PSS thin film was first an inkjet-printed and then covered by an electropolymerized porous PANI layer, creating a PANI–PEDOT:PSS transistor channel. The use of PANI and its pH-dependent doped and dedoped state, a linear response in the pH range of 4–10 was observed, with a sensitivity of 102 ± 22 mV/pH (up to 2x the Nernstian limit).

##### 3.2.1.3 Temperature Response

The thermoelectric properties of thin PEDOT:ClO3 films, electrochemically generated between wireless bipolar electrodes on flexible substrates, were used to create a soft neural thermocouple that demonstrated a response within a biologically relevant range.^307^ Electropolymerized films of naphtalenediimide-triphenylamine (NDI-TPA) showed a temperature-dependent CV response, which could potentially be used to detect changes in temperature.^418^

#### 3.2.2 Electrocatalytic Sensing

Electrocatalytic sensing through investigations and inclusion of specific material is also well suited to electropolymerization due to the possibility of controlled incorporation of dopants, catalysts, or nanomaterials. For example, in 2017 Satyanarayana electropolymerized poly­(2-amino-1,3,4-thiadiazole) (poly-ATD) on a carbon nanotube paste electrode (CNPE) made up of multiwalled carbon nanotubes (MWCNTs).^419^ Characterization showed that the formed *poly*-ATD was strongly linked with the MWCNT carboxyl functional groups. The material combination and fabrication process positively influenced the electrocatalytic response of dacarbazine, an important anticancer drug, resulting in a low LOD (35 nM) and a linear range of 5 × 10^–8^ M – 24 × 10^–6^ M. Selective detection was demonstrated in the presence of electroactive biological interferents (dopamine, serotonin) and measurement in artificial urine samples was possible. In 2019, Farshadinia and Kolahdoozan developed a porous copolymer electrocatalyst building on aniline-based polymers poly­(diphenylamine) (PDPA) and poly­(4,4′-diaminodiphenyl ether) (P4,4′-DADPE), which have shown low performance in previous studies.^420^ Using electropolymerization to carefully control the growth of copolymer poly­(diphenylamine-*co*-4,4′-diaminodiphenyl ether) (DPA-*co*-4, 4′-DADPE) on a carbon paste electrode (CPE), it was possible to optimize the polymer catalytic properties for amoxicillin (AMO) detection. The overall deposition process is simple and green, and result in a good LOD (1 μM) and linear response range (0.02–0.4 mM) of AMO. Recently Castagnola et al. electropolymerized PEDOT/CNT coatings on glassy carbon flexible microelectrode arrays (MEAs) to enable in vivo sensing of tonic serotonin (5-HT).^421^ Through the use of optimized square wave voltammetry (SWV) waveforms and materials, electrochemical oxidation of 5-HT was possible with selectivity over typical interfering species in the brain (epinephrine, dopamine, 3,4-dihydroxyphenylacetic acid, uric acid, and ascorbic acid). Good fouling resistance was observed, and multichannel tonic 5-HT detection was achieved in the mouse hippocampus for both anesthetized and awake head-fixed animals with a record detection period of 1 week.

#### 3.2.3 Enzymatic Sensing

Biomolecules such as enzymes, antibodies, or DNA fragments along with components such as coenzymes, mediators, or stabilizers may be included in the construction of biosensors. The biological recognition elements that lend the prefix to biosensors serve both to contribute to selectivity of the molecular recognition event and to facilitate signal transduction into the optical or electrical domain. These components contribute to the detection and signal transduction process, making biosensors effective in converting biological interactions into measurable signals.^422^ When specifically creating enzymatic biosensors, enzyme immobilization is needed in some form to provide the desired sensitivity. Four main approaches exist for this immobilization – cross-linking, covalent binding to the substrate surface, adsorption, and entrapment.^423^ While there are inherent advantages and disadvantages of each approach, entrapment by electropolymerization offers simplicity, versatility in that it can be applied to a variety of enzymes or other proteins, the possibility to protect the enzymatic activity, and precise entrapment at the electrode of interest improving the catalytic efficiency and potentially enabling direct electron wiring in cases of intimate connection. Depending on the way the sensor transduces biological interactions into detectable signals and the mechanism of charge transfer, enzymatic sensors can be classified into 3 categories: first, second, and third generation. First-generation biosensors directly detect the product or byproduct of an enzymatic reaction; a common example is hydrogen peroxide (H_2_O_2_). Second-generation sensors use mediators to shuttle electrons between the enzyme and the electrode and third-generation sensors eliminate mediators by establishing direct electron transfer between the enzyme and the electrode.

The earliest use of the electropolymerization process to entrap enzymes within conducting polymers at the electrode surface was reported in 1986, separately by Foulds and Lowe^424^ and Umana and Waller.^425^ In both cases, PPy was utilized to entrap glucose oxidase, making use of the possibility to electropolymerize from an aqueous solution and of the capability of the polymer film to provide mass transport as well as electron conduction capabilities. Approximately one year after this first demonstration of enzyme entrapment in a polymer film, Bartlett and Whitaker already include this approach, and a few similar subsequent studies using a variety of polymers (PPy, poly-*N*-methylpyrrole, PANI and polyphenol), as a promising future direction in their review on amperometric enzyme electrodes.^426^ Within 10–20 years further reviews exist based solely on electropolymerization for biosensor development, demonstrating the interest and promise in this method.^422,427^ Initially PPy received the main attention due to restrictions of other monomers, including acidic conditions required for PANI deposition (often too harsh for biomolecules that may be denatured) and the insolubility of thiophene in water. Utilization of, for example, functional groups on the monomer units has aided in overcoming these limitations and enabled the use of PEDOT, PANI, their derivatives, and other conjugated polymer systems for biosensors. In addition to derivative synthesis, the use of process condition variations has greatly increased the possibilities for functional polymer coatings. This may include codeposition of nanostructures, inclusion of nanomaterials, and synthesis of or within ionic liquids.^428^

Glucose sensors have been the most extensively investigated for enzymatic biosensing as a result of the medical importance of glucose (i.e., for managing diabetes) and the stability, and ensuing thorough characterization, of the corresponding oxidoreductase enzyme, glucose oxidase (GOx). After the initial studies mentioned above on the possibility to entrap GOx in polymers films, miniaturization of this method was carried out in 1988 by Shinohara et al. exploring PANI electropolymerization under neutral pH conditions and using a 50 μm platinum fiber.^429^ Miniaturization of sensor size is of general importance to enable targeting of specific biological structures. The microbiosensor fabrication in this work was shown to be feasible and responsive to relatively large concentrations of glucose (10 mM), while larger sensors using the same process conditions showed a response range of 10^–4^ to 5 × 10^–3^ M.

Nanostructuring of electrode surfaces has the potential to improve sensor sensitivity due to effects such as high surface area and unique chemical and physical characteristics. In 2003 Yun et al. investigated nanostructured surfaces and their potential benefit for glucose sensing.^19^ Polymerization of pyrrole in the presence of GOx was carried out on Pt nanowire (NW) bundles (Figure 16a), demonstrating greatly increased sensitivity when compared to planar Pt electrodes. In 2014 Yang et al. used an electrospinning process to create PLLA nanofibrils (NFs) on small neural probe electrodes (1394 um^2^), followed by galvanostatic electropolymerization of EDOT monomer, poly­(sodium-p-styrenesulfonate) (PSS), and GOx on the Pt electrode surface (Figure 16b).^430^ A larger amperometric response was observed for the PEDOT NFs-GOx biosensors when compared to planar films. The sensitivity was compared at biases of 300 mV as well as 700 mV, suggesting a direct electron transfer mechanism. Application-specific structures such as microneedles for smart patches have also been explored in combination with conducting polymer entrapped enzyme.^433^ In this work, Invernale et al. immobilized GOx within PEDOT:PTSA (*p*-toluenesulfonic acid) on a platinum-coated stainless steel in-line 2D microneedle array with linear response in the range of 2 – 24 × 10^–3^ M (S/N = 10.7), a relevant range for glucose monitoring in human blood samples.

**Figure 16:**
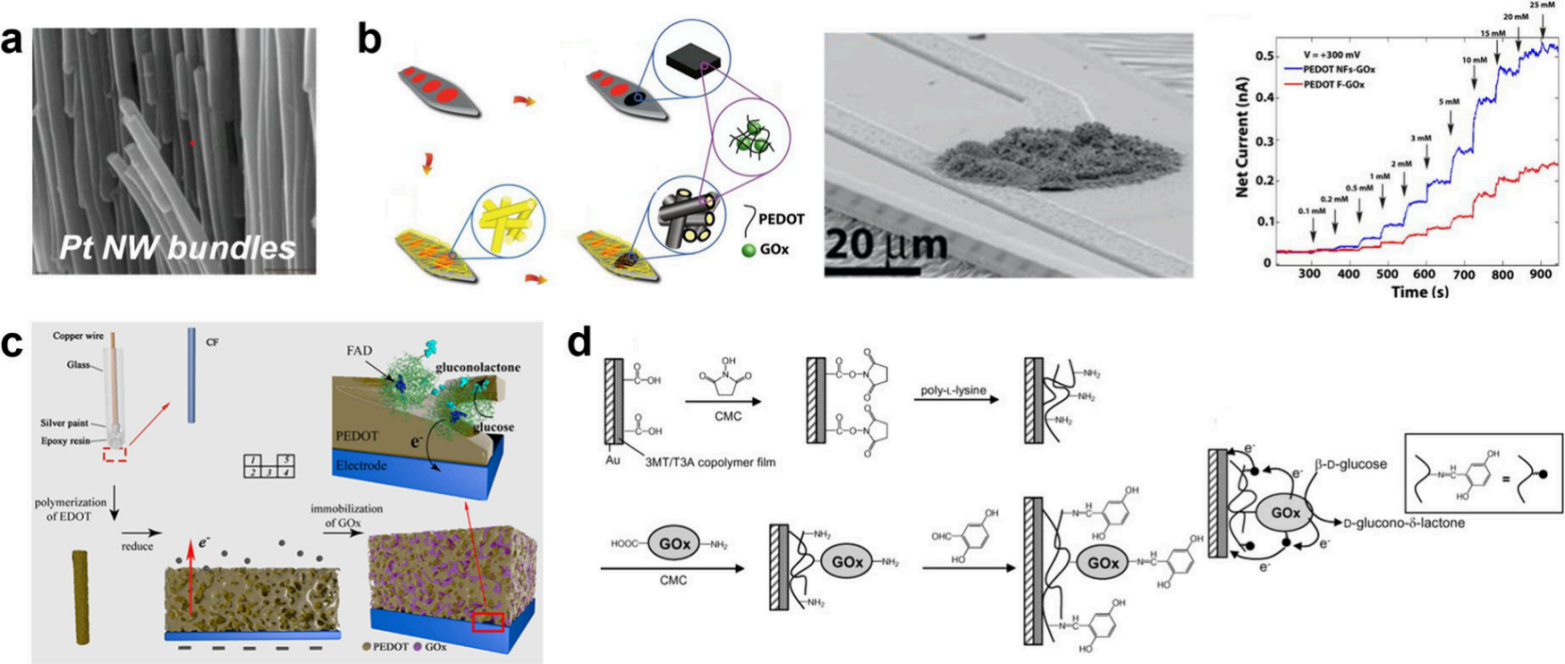
Enzymatic glucose sensors. a) Pt nanowire bundles are created through an electrochemical process and used as a high catalytic surface area substrate for the immobilization of GOx in a PPy film and for the detection of glucose.^19^ b) An electrospinning process is used to coat neural electrodes, followed by the electrochemical deposition of PEDOT:PSS to entrap GOx. Improved performance over planar films was shown (right panel).^430^ c) 7–8 μm diameter carbon fibers are used to electropolymerize PEDOT:ClO_4_ and immobilize GOx. Direct electron transfer is confirmed and the overall system holds potential for use as an implantable glucose sensor platform.^431^ d) Various covalent surface binding strategies are explored for GOx and DHP redox mediator groups. The use of PLL (depicted here) proved to be most effective when compared to the use of differing chain lengths of alkylenediamines (C2–C12, not shown).^432^ Panel a was reproduced with permission from reference,^19^ Copyright Society of Photo-Optical Instrumentation Engineers (SPIE). Panel b was reproduced with permission from reference 430, Copyright John Wiley & Sons, Inc., 2001. Panel c was reproduced with permission from reference 431. Copyright The Electrochemical Society (“ECS”), 2020. Panel d was reproduced with permission from reference 432, Copyright Elsevier, Ltd., 2025.

Alternative approaches to entrapment within polymer films include noncovalent interactions, such as adsorption or electrostatic binding, as well as covalent attachment using chemical methods. When adsorbing biomolecules onto polymer films, weak intermolecular forces facilitate their attachment and preserve their activity while electrostatic binding leverages the natural charges of biomolecules and polymers to create a more stable, yet noncovalent bonds. For example, in 2007 Patil et al. used mild steel substrates to electropolymerize poly­(o-anisidine) (POA) films and subsequently immobilized GOx by physical adsorption through simple immersion of the high surface area films in GOX-containing PBS.^434^ It is shown that GOx is present in the resulting biosensor with amperometric measurements demonstrating sensitivity to glucose in the range of 2–20 mM. The suggested electron transfer mechanism is direct transfer between GOx and POA. Storage stability was shown up to 15 days, with a subsequent rapid decrease in the sensor response, likely due to the weak attachment forces when using the adsorption method. In a more recent study, David et al. utilized electrostatic interactions to carry out a layer-by-layer deposition of alternating chitosan, GOx, and nitrogen-doped graphene (chit^+^(NG + GOx)) and PSS^–^ on a gold substrate with an electropolymerized PEDOT:PSS film.^173^ The multilayer system depends on electrostatic interactions between the positive (chit^+^(NG + GOx)) layer and the SO_3_^–^ groups of the PEDOT:PSS substrate, as well as with the negatively charged PSS^–^ interlayers. Surface plasmon resonance measurements were used to show the successful deposition of each layer and fixed potential amperometry exhibited linear glucose sensitivity in the range of 0.1 and 1.4 mM with a 41 μM limit of detection (LOD). In 2020, Chen et al. also made use of electrostatic forces to create an implantable microbiosensor based on a PEDOT-modified carbon fiber (CF) electrode.^431^ Electropolymerization was first carried out on the 7 μm diameter CF in an EDOT/LiClO4 solution. Afterward, a 20 min treatment at −0.25 V bias was applied to remove the anions as completely as possible. Finally, treatment in GOx solution was carried out at +0.75 V for 20 min to immobilize the negatively charged GOx within and on the polymer film (Figure 16c). A linear sensitivity range of 0.5 – 15 mM was shown as well as 60 days of storage stability with minimal loss in sensor response.

Covalent coupling of GOx is an approach that holds the potential to create more stable chemical bonds, however may also limit the enzyme activity depending bond length and material matrix.^435^ while the enzyme may be directly bonded to the electrode material, cross-linking agents such as gluteraldahyde (GA) have also proven useful in the covalent coupling of enzymes to electrodes due to the possibility to bond to form covalent bonds with amino groups (−NH_2_) or lysine residues.^432,436–438^ A study by Kuwahara et al. in 2005 on the immobilization of both GOx and electron-mediating groups (2,5-dihydroxyphenyl (DHP)) on conducting polymer electrodes (3-methylthiophene (3MT) and thiophene-3-acetic acid (T3A)) investigated the effects of different bonding methods (directly to the carboxyl groups of the T3A units or to poly-l-lysine (PLL)), as well as differing chain lengths of alkylenediamines (C2–C12) for bonding the DHP mediator (Figure 16d).^432^ Optimal material ratios and chain lengths were found, with the best glucose sensitivity revealed when using the longest alkylenediamine chain length and further improved when employing PLL bonded at the polymer surface and without the use of alkylenediamines. Tamer et al. made use of Au nanorods to increase the sensor catalytic activity and GA immobilization of GOx at the sensor surface.^437^ The Au nanorods were modified with PSS to create negative charges and facilitate the doping process during electropolymerization of PANI. Afterward, GA was coated on the polymer surface, covalently bonding to PANI as well as enabling bonding of the following GOX treatment. DIW rinsing was carried out after each step to remove excess/unbound GA or GOx. A low LOD of 5.86 μM and a linear rage of 0.0176 – 1 mM were demonstrated. Recently, Tan et al. also utilized Au nanostructures in combination with GOx/GA functionalization to achieve a LOD of 8.46 μM, a linear range of 1.169 × 10^–2^ – 10 mM, and storage stability of up to 4 weeks.^438^ In this work, pencil graphite electrodes were modified with a PEDOT derivative (4-(dihexylamino)-9,12-bis­(2,3-dihydrothieno­[3,4-*b*]­[1,4]­dioxin-5-yl)-7H-benzo [de]­benzo­[4,5]­imidazo­[2,1-*a*]­isoquinolin-7-one (EDOTBN)) and AuNPs were subsequently deposited in the film using cyclic voltammetry scanning. An immobilization step using GOx and GA in solution was then carried out to complete the sensor.

Further enzymatic sensors have been developed, building on the initial GOx-based approach but targeting other analytes by using the corresponding oxidase enzymes. These sensors function similarly, making use of target analyte interactions and the resulting biochemical reaction, which produces a measurable byproduct, such as hydrogen peroxide, or generates electrons directly. A great deal of analyte possibilities exist, of which many have also been explored, including glutamate, cholesterol, lactate, acetyl choline, urea, and xanthine.

Cholesterol oxidase (COD) was electropolymerized within PPy on Pt electrodes already in 1993, utilizing the first generation sensor concept of H_2_O_2_ production and reaction at the catalytic Pt surface.^439^ While some challenges existed regarding reproducibility and response to interferants (ascorbic acid, uric acid), good response to cholesterol was observed. Flow-through and flow-injection systems were used together with the developed cholesterol sensor and a polycarbonate membrane (3 um pores) proved useful in reducing noise and improving reproducible results.

In 1995, Cooper et al. employed a 25 μm Pt microelectrode to polymerize PPy and glutamate oxidase (GLOD).^440^ Glutamate is a principal neurotransmitter in the mammalian brain and, accordingly, a main aim of this work was to enable in vivo analysis. Therefore, the 25 μm microelectrode size was used and the main goals were to investigate the detection limit, the stability, and the response to interferents. The GLOD enzyme was first adsorbed onto electrode surface, followed by further entrapment during electrochemical polymerization. Promising results regarding the goals were shown, in particular for the first two aims (LOD: ∼ 100 uM, ∼ 15% loss in in performance over 30 days), while the effect of interferents were reduced, but remained potentially problematic. It was hypothesized in this work that a thicker polymer layer would aid in reduction of interferent signals, however at the expense of the sensor response time.

Further studies have looked into reduction of interferents through methods such as utilization of an overoxidized PPy film with entrapped COD to provide anion-exclusion properties^441^ or the coimmobilization of ascorbate oxidase and coating the sensor surface with a cationic polymer, polyethylenimine.^440,442^ The latter studies, carried out by Rahman et al., utilize a polythiophene derivative, poly-5,2:5,2-terthiophene-3-carboxylic acid (poly-TTCA) to immobilize choline oxidase (ChO), a bienzyme of ChO/horseradish peroxidase (ChO/HRP) as well as GLOD and a coimmobilization of GLOD/ascorbate oxidase (AsOx). The polymer carboxylic acid groups are used to bind the enzymes to the surface, and in the study on GLOD/AsOx coimmobilization, AsOx is cleverly utilized to prevent interference from ascorbate (as AsOx does not produce H_2_O_2_). This approach along with the achieved sensor performance, enabled an in vivo study in the rat brain to monitor glutamate levels as a result of cocaine injections.

A xanthine biosensor was constructed by immobilization of the enzyme xanthine oxidase onto an electropolymerized film with a monomer composed of a dithieno­(3,2-*b*:2′,3′-*d*)­pyrrole backbone and an alkyl-tethered primary amine side chain.^175^ The sensor was able to rapidly and accurately quantify the xanthine concentration in PBS without interference from uric acid, ascorbic acid, glucose, and sodium benzoate. It also performed well in a highly complex matrix, detecting an increase in xanthine concentration with the age of chicken muscle samples.

More recent examples that make use of cross-linking methods or electrostatic interactions include studies on a superoxide biosensor^443^ and a label-free electrochemical lactose biosensor,^444^ both including carbon-based nanomaterials in their processes. In the former study by Braik et al., a PEDOT/CNT/superoxide dismutase (SOD) stack (Figure 17a) was created with GA utilized in the final step to aid in bonding the enzyme to the CNT layer embedded in a chitosan matrix. This sensor, aimed at superoxide determination in healthy and diseased tissues, as well as the antioxidant activity of food, beverages, and pharmaceutical formulations, exhibited high sensitivity compared to previous studies, minimal effect from interferents, and good stability over 2 months. Nguyen et al. investigated the coimmobilization of β-galactosidase (β-Gal) and GOx to make a lactose biosensor.^444^ In this case, graphene electrodes were fabricated using a transfer process, followed by electrochemical deposition of poly­(1,5-diaminonaphthalene) (P­(1,5-DAN)). GA was then deposited to bind to the available amino groups of the polymer and subsequently to cross-link the (β-Gal) and GOx enzymes. Improved lactose sensitivity was demonstrated, as well as successful determination of lactose content in model samples.

**Figure 17:**
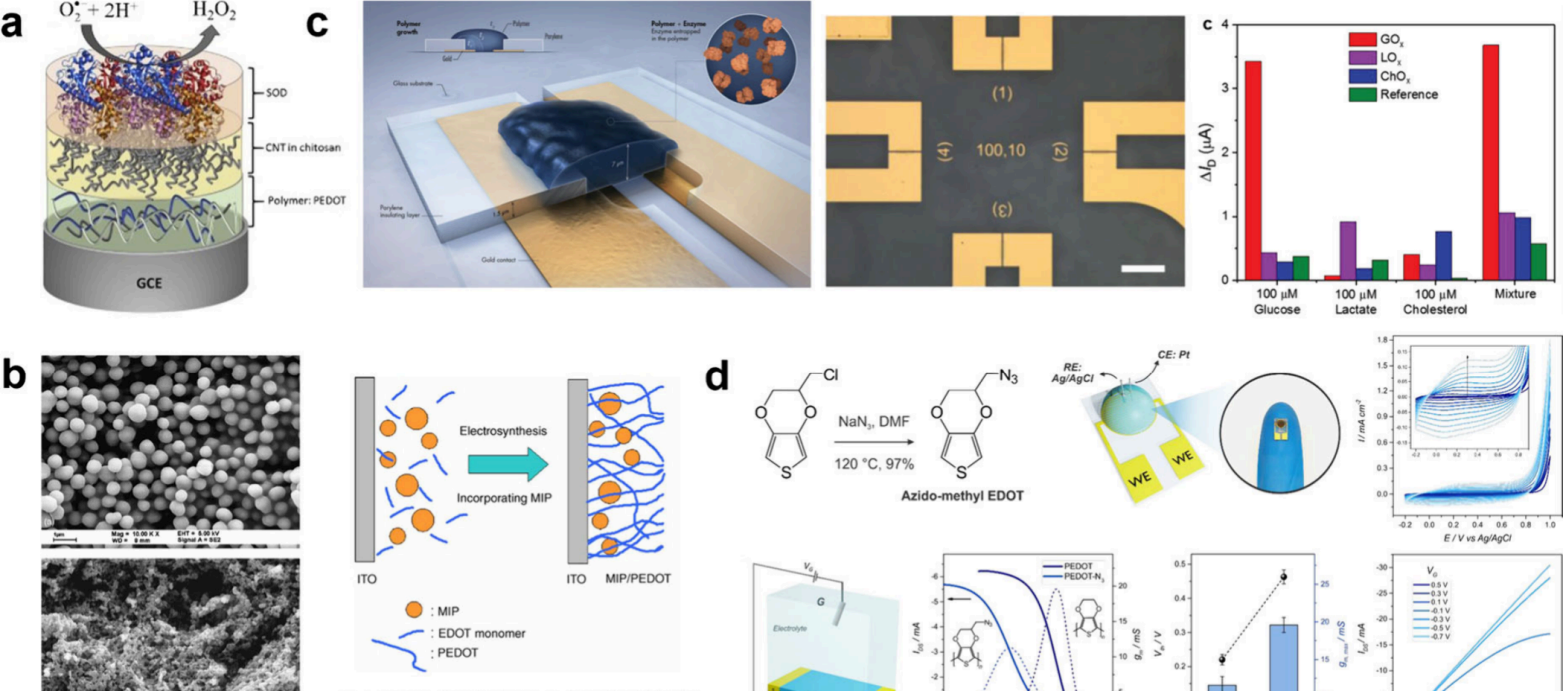
a) A superoxide sensor fabricated using a stacked approach of an electropolymerized film (EDOT with NaSS), followed by CNTs in a chitosan membrane, and last a layer including the superoxide dismutase enzyme along with glutaraldehyde to aid in bonding.^443^ b) A morphine sensor based on MIP microspheres is created first using a thermal radical polymerization step with a methacrylic acid monomer and trimethylolpropane trimethacrylate. Once the MIP microspheres are generated (upper left panel, with a comparison to bulk polymerization in the bottom panel), electropolymerization is used to embed them in a PEDOT:ClO_4_ film (right panel), improving the morphine sensitivity.^445^ c) A multimetabolite OECT sensor array is developed using electropolymerization to deposit copolymer p­(EDOT-*ran*-EDOTOH) along with various enzymes (GOx, cholesterol oxidase, lactate oxidase) across the transistor channel area in close proximity of one another (middle panel). Selectivity for the targeted analyte is observed (right panel) with oxidation of the CP film by enzymatically generated H_2_O_2_ as the suggested sensor mechanism.^446^ d) “Clickable” OECTs based on an azide-derivatized EDOT monomer are shown, utilizing the PEDOT-N_3_ functionality to allow bonding or ‘clicking’ of ethynyl-ferrocene, an alkyne-bearing biotin-PEG_4_, and a thrombin specific DBCO-derivatized HD22 aptamer.^447^ Panel a reproduced with permission from reference 443, Copyright Elsevier, Ltd., 2016. Panel b reproduced with permission from^445^, Copyright Elsevier, Ltd., 2005. Panel c reproduced with permission from reference 446, Copyright John Wiley & Sons, Inc., 2020. Panel d reproduced from reference 447 (CC BY).

#### 3.2.4 Non-enzymatic Sensing

While enzymes have the advantage of being highly specific to their target analyte, they often suffer from chemical instability, sensitivity to environmental factors, are expensive, and may require complicated purification steps.^448^ Due to these challenges, nonenzymatic sensors have also been largely explored to provide robust and durable alternatives to enzyme-based sensing. OMIEC-based nonenzymatic sensors derive selectivity through the integration of either 1) a recognition element like a tethered small molecule, aptamer, or polypeptide or 2) a molecularly imprinted polymer (MIP). An immense amount of research has been carried out across these fields and reviews may be found dedicated to specific approaches or analytes, including nanomaterial-based glucose detection,^449^ sweat analysis,^450^ cholesterol,^451^ creatinine,^452^ antibody, aptamer, and MIP-focused (for pesticide residues).^453^

##### 3.2.4.1 Material Functionalization

Functionalization approaches are well-suited to conducting polymers, whose highly tunable chemical structure enables the attachment of diverse molecules, proteins, and functional groups. In 2009, Bazaco et al. utilized this property to create an EDOT derivative covalently linked to the RNA nucleobase uracil.^454^ This functionalization enables the molecular recognition of its complementary nucleobase, adenine, which is significant in areas such as disease diagnosis and monitoring, cancer research, and environmental monitoring. Electropolymerization of the uracil-functionalized EDOT monomer on Pt electrodes resulted in successful recognition experiments in aqueous media, demonstrating the largest changes in peak potential and electroactivity for the complementary base adenine. In another study on antibody–antigen sensors, to provide sensitivity to alpha fetoprotein (AFP), an important tumor marker, Cui et al. electrodeposited a PEG doped PEDOT nanocomposite, using a 4-arm PEG terminated with thiol groups.^455^ This PEDOT/PEG composite maintains excellent biocompatibility and enables further functionalization. In this case, the Au nanoparticle (AuNP) interaction with the thiol groups is first taken advantage of, followed by an incubation step to electrostatically immobilize the AFP antibody (Ab). Using electrochemical impedance spectroscopy (EIS), the Ab/AuNPs/PEDOT/PEG/GCE biosensor showed good sensitivity (linear response from 0.001 fg/mL to 10 fg/mL), a low LOD (0.0003 fg/mL), favorable selectivity, and the possibility to assay the AFP antigen in 10% (V/V) serum samples.

##### 3.2.4.2 Molecularly Imprinted Polymers

MIP-based sensing makes use of a templating process to create specific binding sites within a synthetic polymer matrix, resulting in recognition of a target molecule through rebinding events. The polymerization and templating process may be carried out using a variety of methods. In 2005, Ho et al. combined precipitation polymerization to create morphine hydrochloride-templated microspheres and subsequently employed electropolymerization of PEDOT to immobilize the MIP spheres on an ITO electrode (Figure 17b).^445^ The MIP/PEDOT-modified electrode showed improvement compared to the use of non-MIP particles, as well as a good linear sensitivity of 41.63 A/cm^2^ in the range of 0.1 – 2 mM. Recently Zidarič et al. achieved insulin sensitivity in single droplets (50 uL) using a MIP sensor created by electropolymerization.^456^ PPy was deposited on screen-printed carbon electrodes (SPCEs) in the presence of insulin and afterward this protein template was removed by electrochemical cleaning or solvent (NaOH) extraction. The resulting MIP–SPCE, presented as a disposable sensor based on the ease of fabrication and materials cost, showed a linear range of 20.0 pM – 70.0 pM, a LOD of 1.9 pM, and was successfully used with pharmaceutical sample to detect insulin.

#### 3.2.5 Transistor-Based Sensing

Transistor-based biosensors offer a variety of advantages compared to traditional electrode-based sensors. Through the use of devices such as the electrolyte-gated field-effect transistor (EGFET) or the OECT. high signal amplification may be achieved, improving analyte sensitivity, while operating at low voltages and maintaining the possibility for flexible device structures.^24,31,457^ The high amplification is a result of the inherent transistor properties as well as the particular sensitivity to the physical properties of the channel/electrolyte and gate/electrolyte interfaces. Thus, both channel and gate interfaces have been explored for functionalization, using analogous methods to the electrode-based sensor development: adsorption, electrostatic interaction, entrapment, covalent bonding/cross-linking.^70,458^ In 2019 Wustoni et al. employed electrochemically polymerized channels of copolymer p­(EDOT-*ran*-EDOTOH) to entrap specific enzymes of interest (GOx, ChOx, and LOx) on OECT channels to create a multianalyte array for sensing glucose, cholesterol, and lactate.^446^ Although bridging the insulating region of planar OECT structures can be challenging,^459^ thick polymer layers accomplished this connection and successfully demonstrated enzyme entrapment through analyte selectivity (Figure 17c). The sensing mechanism (change in channel current, *I*_*D*_) is attributed to the oxidation of the CP film by enzymatically generated H_2_O_2_. While some interference was present, the overall specificity to the concentration of the target metabolites was shown in a common solution of complex media. Recently, Fenoy et al. developed graphene field-effect transistors functionalized with electropolymerized nanofilms for the detection of glucose in urine.^460^ Reduced graphene oxide was first deposited on Au interdigitated source/drain contacts. Afterward, copolymer poly­(3-amino-benzylamine-*co*-aniline) (PABA) was deposited on the graphene FET channel and GOx was electrostatically immobilized using the amine–imine moieties of PABA. A low LOD of 4.1 μM was shown along with a linear range of 10 μM – 1 mM and the possibility to sense glucose in complex solutions such as urine. Similarly, an effective acetylcholine biosensor was constructed by electrostatically adsorbing acetylcholine esterase (AchE) on a PABA-modified reduced graphene oxide field effect transistor.^461^ Immobilized AchE cleaves acetylcholine into choline and acetic acid, which leads to a reduction in the pH in the vicinity of the FET channel and allows for electrochemical signal transduction in the absence of enzyme-catalyzed redox reactions. The electropolymerized PABA serves several functions, including assisting in the electrostatic immobilization of the AchE, preventing denaturation of the enzyme, and improving the pH sensitivity of the device.

OECT-based sensors are advantageous in the same regard as for enzymatic sensing and have received significant attention over the last 10–15 years. Various approaches are used for conducting polymer deposition, due to the challenge of bridging the source and drain contacts with electropolymerization. In some cases, the transistor gate is functionalized, while in other cases a first channel layer may be formed by other deposition methods. The latter is the case in work by Hai et al. on detection of whole human influenza A virus, where a thin PEDOT:PSS layer is first spin-coated onto the channel area.^462^ Copolymerization of EDOT and a derivative bearing an oxylamine moiety (EDOTOA) is then carried out on the channel, followed by incubation in a solution of 2,6-sialyllacotse (Sia-α2,6′-Lac) or 2,3-sialyllacotse (Sia-α2,3′-Lac) for 12 h to allow a glycosylation reaction to take place. Once prepared, the 2,6-sialyllactose-functionalized sensor demonstrated response to hemagglutinin, a spike protein expressed on the surface of human influenza A virus (whereas the avian influenza viruses preferentially 2,3- sialyllactose). Sensitivity in the range of 0.03 – 1 hemagglutination units (HAU) was shown, with a limit of detection (0.025 HAU) nearly 2 orders of magnitude lower than conventional rapid human influenza tests. Janardhanan et al. also made use of a spin-coated PEDOT:PSS layer, with an electropolymerized upper layer of poly­(EDOT-COOH-*co*-EDOT-EG3) with and without nanotubular structure controlled by the use of solvent during deposition.^463^ This provided the basis for their sweat cortisol sensor, an adrenocorticosteroid stress hormone. The copolymer was incubated in a PBS solution of EDC/s-NHS to activate the carboxyl groups and afterward a solution of anticortisol antibodies was drop-cast onto the channel area. The completed sensor was capable of real-time cortisol detection with a greatly improved sensitivity for the nanotube polymer structure when compared to the planar film, linear in the range of 1 fg/mL to 1 μg/mL and with an LOD of 0.0088 fg/mL. In 2022, Fenoy et al. introduced the concept of “clickable” OECTs by synthesizing an azide-derivatized EDOT monomer, azidomethyl-EDOT (EDOT-N3).^447^ PEDOT-N3 OECT channels were formed via electropolymerization under a low electric field on shorted interdigitated Au source/drain contacts. To assess the availability of azide moieties for anchoring biorecognition elements, a series of “click” reactions were demonstrated. These included reactions with an ethynyl-bearing redox probe, ethynyl-ferrocene (Et-Fc); an alkyne-bearing redox couple, N-(3-butynyl)­phthalimide (N-But); acetylene-PEG4-biotin; and a thrombin-specific DBCO-derivatized HD22 aptamer (Figure 17d). Successful anchoring was confirmed through redox peaks observed in CV curves after binding of the redox probes, which also enabled the estimation of azide moiety density. In the case of biorecognition elements, a shift in OECT threshold voltage, *V*_*T*_, was observed upon binding of NeutrAvidin to the acetylene-biotin. Leveraging this platform and the stable biotin–avidin complex, the biotin-modified OECTs were exposed to streptavidin-conjugated horseradish peroxidase (HRP). Successful binding was demonstrated by measuring changes in *I*_*D*_ after the addition of varying H2O2 concentrations. Finally, the affinity of the DBCO-aptamer for thrombin was evidenced by alterations in OECT transfer characteristics, including *ΔI*_*D*_ and shifts in *V*_*T*_. Overall, PEDOT-N3 OECTs were developed and characterized, exhibiting good transistor performance and flexibility for multiple “click” reactions, underscoring their potential in biosensing applications.

MIP use in OECTs has been explored for a variety of analytes including uric acid (UA),^464^ dopamine,^465^ and glucose,^357^ typically carrying out the electropolymerization and molecular imprinting process on the gate electrode. In 2022, Tao et al. utilized reduced graphene oxide (rGO) modified cotton fibers as well as carbon fibers to create a MIP-based OECT UA sensor aimed at monitoring the concentration in urine or human blood to aid in management of diseases such as gouty arthritis, hyperuricemia and hypertension.^464^ The gate was prepared using a carbon fiber to first electropolymerize PEDOT:ClO4 and afterward a polydopamine-UA MIP membrane from a solution of dopamine as a monomer and UA as a template. To remove the UA template, the fiber was then immersed in an acetic acid/methanol solution. While the rGO increased the surface area and performance of the OECT channel, the MIP-modified carbon fiber gate attained a sensitivity of 100 μA per decade in the range of 1 nM to 500 μM and was capable of UA detection in artificial urine samples with good accuracy. The fiber-based concept additionally allows for integration into wearable detection platforms, promising for medical diagnosis applications. The same year Tang et al. developed a NIP-based process for dopamine sensors that maximizes selectivity.^465^ While PEDOT:PSS is used for the channel material, a PPy film was electrodeposited on the Pt gate in the presence of NaCl and with dopamine as the template. Afterward the dopamine is removed by immersion in ethanol and the MIP is then overoxidized, a method discussed in Section 3.2.3 to improve selectivity. The overoxidized MIP sensor indeed shows greatly enhanced selectivity (for up to 10x ascorbate acid) in the range of 0.4 μM - 10 μM dopamine, while maintaining a low overall LOD (34 nM). Very recently, Kousseff et al. utilize PEDOT–PBA for nonenzymatic glucose sensing.^357^ Making use of the strong interaction between EDOT-PBA and hexose sugars, this work explores both the sensor response of an electropolymerized film of PEDOT:PBA as the OECT gate, and a MIP film deposited with a methylated analogue of glucose (methyl α-d-glucopyranoside) present as the template. While sensor characterization using a PEDOT:PSS channel and the nontemplated PEDOT–PBA demonstrated a higher normalized, the MIP gate showed a slightly lower LOD (22.3 μM vs 28.2 μM), better reproducibility between fabricated sensors, and a single linear response over the range of 10 μM to 10 mM (while the nontemplated device had 2 linear regimes).

### 3.3 Neuromorphic Synapses Enabled by Electropolymerization

Silicon-based integrated circuits memorize and process information using binary digits. In contrast, biological neural systems develop memory, sensation, and learning through neural plasticity, which dynamically tunes the strength of synapses connecting billions of neurons. To mimic neuroplasticity, silicon electronics require complex circuitry to simulate a single synapse, consuming much more power than a biological synapse, which uses as little as ∼ 10 fJ per synaptic transmission.^466^ OECTs employing OMIECs as ion-tunable channel materials demonstrate biomimetic synaptic behaviors including short- and long-term plasticity and associated learning capability.^33^ These devices offer improved biomimicry, simpler architecture, and significantly lower energy consumption compared to silicon circuits. This section discusses a novel type of artificial synapse with evolvable synaptic weight through electropolymerization and its modular integration with artificial receptors and neurons to achieve advanced neuromorphic functionalities. Additionally, we review artificial synapses and networks featuring evolving dendritic connections and topological plasticity to guide future research in brain-inspired neural networks.

#### 3.3.1 Organic Electrochemical Synapse

An archetypical OECS adopts a three-terminal OECT design in which the gate supplies input voltage to modulate the output current in the channel OMIEC. In this configuration (Figure 18a), the gate is analogous to the presynaptic terminal, the drain emulates the postsynaptic terminal, and the conductance (G) of the channel dictates the synaptic weight. The conductance is governed by *G* = σ·(*Wt*/*L*), where σ is the OMIEC’s conductivity, and *W*, *t*, and *L* are the channel’s width, thickness, and length, respectively.

**Figure 18:**
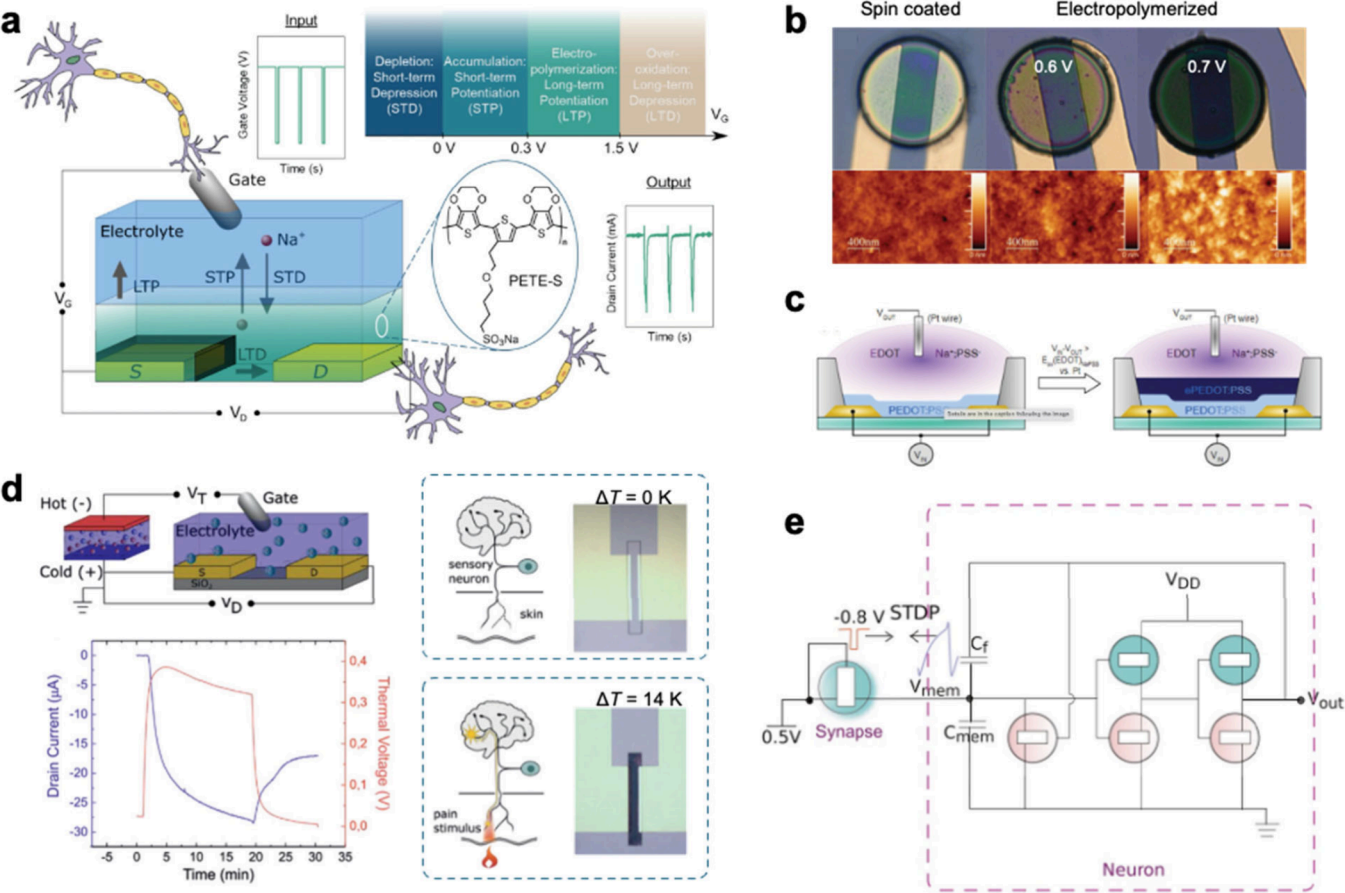
Eletropolymerization of OMIECs for evolvable neuromorphic devices. (a) Schematic illustration of a typical evolvable OECS, formed by electropolymerization of ETE-S in the transistor channel. Panel adapted from reference^42^ (CC BY 4.0). (b) Microscopic images (top row) and AFM height images (bottom row) of PEDOT:PSS films prepared by spin coating (left) and electropolymerization (middle, 0.6 V; right, 0.7 V). (c) Illustrations of an PEDOT:PSS-based OECS before and after the electropolymerization of EDOT on top of spin-coated PEDOT:PSS channel. Panels b) and c) are adapted from reference 473 (CC BY-NC-ND 4.0). (d) Schematic illustration of the iTEG-OECS device structure (top left), the recorded variation in drain current in response to the generation of thermal voltage (bottom left), and the channel formation process emulating the reinformcement of sensory neurons in response to a stimulus. Panel adapted from reference 476 (CC BY 4.0). (e) Equivalent circuitry layout showing the integration between an evolvable OECS and spiking OECN. Panel adapted from reference 43 (CC BY 4.0).

A conventional OECT has a fixed channel geometry but tunable conductivity. The voltage bias from the gate drives mobile ions from the gating electrolyte into (or out of) the OMIEC channel’s bulk, doping or dedoping the polymer chains. This yields a temporary change in the charge carrier density and mobility, which facilitates the emulation of short-term plasticity in an OECS, as the ion migration-induced conductivity variation in OMIEC is a transient effect lasting from milliseconds to seconds. However, when electrochemical doping is the sole measure, achieving long-term plasticity in an OECS becomes challenging as the injected ions tend to diffuse back to the electrolyte once the gating voltage is removed. To address this challenge, researchers have implemented engineering approaches at the circuitry level, including disconnecting the gate using a physical switch^467^ and incorporating a floating memory at the gate terminal^468^ to inhibit the self-discharge of doped OMIECs. OMIEC chemical and phase design has also been explored to control ion diffusion kinetics and extend the charge trapping period. Examples include increasing OMIEC crystallinity through thermal annealing^469^ and side-chain modification,^470^ as well as blending active OMIECs with passive ion-blocking materials like polytetrahydrofuran (PTHF)^471^ to enhance nonvolatile synaptic state retention. Despite these advances, these methods still cannot alter the fundamentally enthalpy-governed, metastable nature of doping-induced conductance states.

*Operando* electropolymerization offers a bottom-up approach for achieving long-term potentiation (LTP) and depression (LTD) in evolvable neuromorphic devices. Instead of controlling ion migration, an evolvable OECS directly alters its OMIEC channel’s geometry—typically thickness—to obtain thermodynamically stable, nonvolatile synaptic weights. This growth or removal in conducting substance enables the OECS to access numerous conductance states spanning several orders of magnitude. In practice, an evolvable OECS uses an electrolytic solution containing OMIEC monomers between the gate and channel area. Applying a positive bias to the drain (e.g., 1 V vs Ag/AgCl for ETE-S^42^) or a negative bias to the gate (e.g., −0.6 V vs Ag/AgCl for ETE-PC^355^) effectively oxidizes the monomers and initiates propagation at the drain terminal. This leads to long-lasting LTP, mimicking the expression of new receptors in biological synapses. Conversely, LTD can be achieved by applying excessively negative voltage pulses (e.g., −2 V vs Ag/AgCl for ETE-S^42^) to the gate, overoxidizing the OMIEC channel and reducing its conductance. For evolvable OECSs based on the ETE monomer family, the affinity between different ETE monomers and substrates has emerged as a critical factor controlling lateral polymer growth,^76^ leading to tunable LTP behavior of OECSs with various monomer–substrate combinations. EDOT and its derivatives have also been widely used to produce evolvable OMIEC channels.^472,473^ Studies reveal that PEDOT:PSS grown through electropolymerization exhibits different surface morphology, volumetric capacitance, and mobility compared to spin-coated PEDOT:PSS films (Figure 18b).^473^ By using a spin-coated PEDOT:PSS channel and then electropolymerizing a new PEDOT:PSS layer on top (Figure 18c), one can simultaneously tune the channel’s geometry and electrical properties, enabling various long-term plasticity behaviors in a single OECS platform. Beyond developing evolvable channels, electropolymerization has also been used to modulate the thickness and capacitance of evolving PEDOT:PSS gates in OECSs.^474^ As the gate’s capacitance increases, the gating voltage couples more effectively to the channel, increasing its transconductance. This provides an alternative approach to achieve LTP while maintaining the channel’s integrity.

The modular integration of evolvable OECSs with presynaptic artificial receptors or postsynaptic artificial neurons can yield biomimetic circuits with advanced neuromorphic functions. Human skin detects multimodal stimuli—pressure, vibration, humidity, and temperature—thanks to diverse receptors in the epidermis and dermis.^475^ To achieve biomimetic stimuli detection and processing, researchers have developed artificial receptors such as ionic thermoelectric generators (iTEGs).^476^ When coupled to an OECS, an iTEG regulates its channel growth in response to temperature changes, mimicking the reinforcement of a sensory synapse relaying thermal information to the brain (Figure 18d). By further incorporating a piezoelectric pressure sensor to the drain of the iTEG-powered OECS, both thermal and pressure stimuli control channel growth. This configuration enables coincidence detection: OECS conductance evolves only when both temperature and pressure stimuli reach their respective thresholds. Mirroring biological synapse-neuron interactions, artificial synapse and neuron integration has been demonstrated by using an evolvable OECS’s drain current as input for an organic electrochemical neuron (OECN) (Figure 18e).^43^ In this setup, the OECN’s spiking membrane voltage (*V*_mem_) serves as postsynaptic feedback to the OECS. Consequently, the synaptic weight growth of the OECS via electropolymerization is modulated by the relative timing between presynaptic pulses and *V*_mem_ peaks. This process showcases spike-timing-dependent plasticity (STDP), which controls synaptic strength and, in turn, the OECN’s firing frequency, enabling associative Hebbian learning.

#### 3.3.2 Dendritic Synapses and Networks

Organic dendritic synapses represent a distinct subclass of evolvable neuromorphic devices. While conventional organic electrochemical synapses (OECS) typically have a predefined channel shape and an OMIEC film as the channel, an organic dendritic synapse grows its channel on demand, forming a fractal pattern/morphology (Figure 19a) that closely resembles dendrites in biological neurons, leading to dynamic and reconfigurable circuits. The first demonstration of OMIEC dendrites dates back to 1993 when Curtis et al. demonstrated dendritic growth and interconnection by electropolymerizing 3-methylthiophene.^477^ Two years later, Fujii et al. constructed a three-terminal device for the first time using a polypyrrole (PPy)-based dendritic channel and reported long-term potentiation (LTP) behavior, indicative of learning and memory, in the channel current in response to gate pulses.^478^

**Figure 19:**
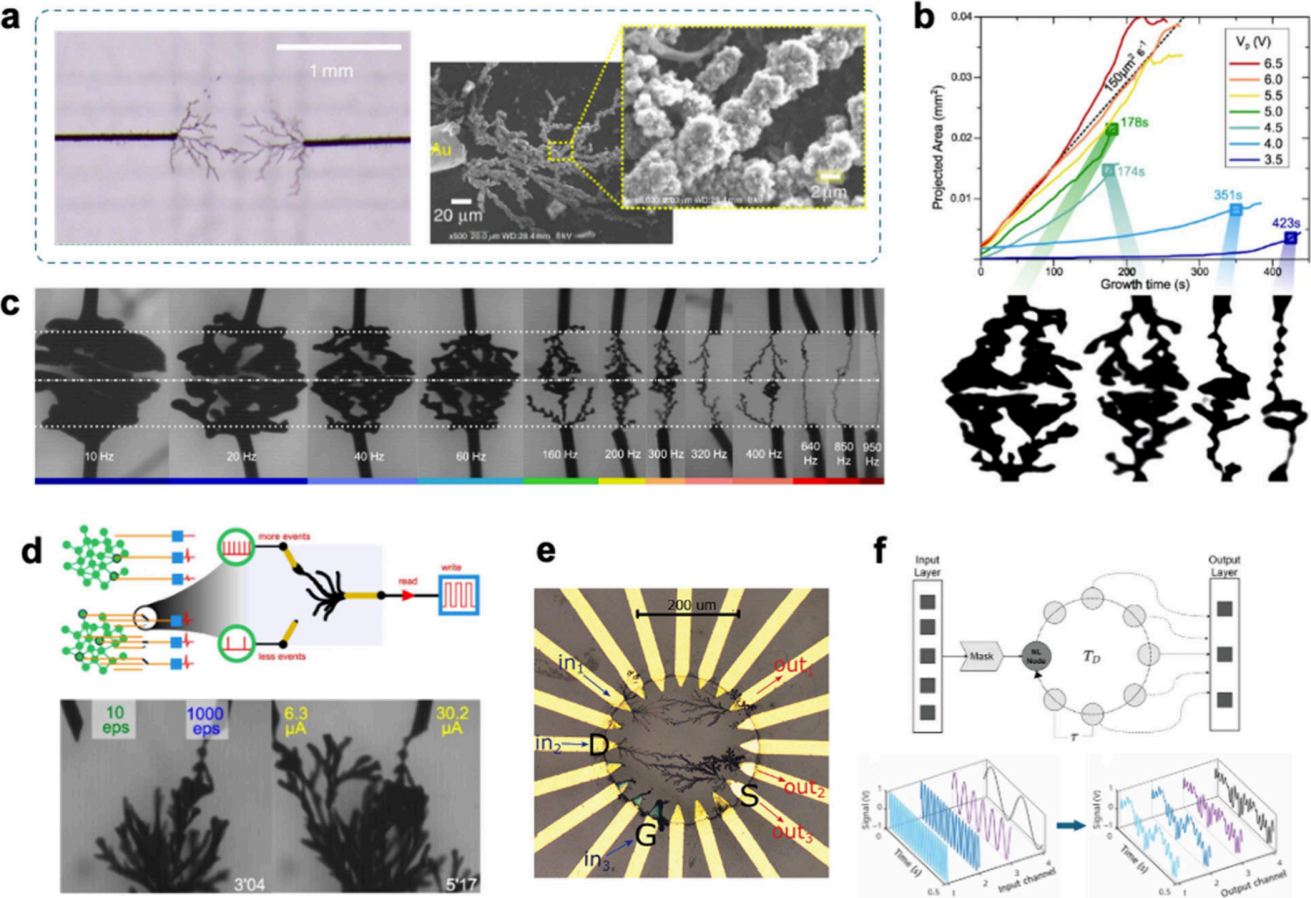
Dendritic synapses and networks. (a) Optical microscopic image (left) and SEM images of the dendritic PEDOT fibers grown by AC bipolar electropolymerization. Panel adapted from reference 71 (CC BY 4.0). (b) Projected area over time showcasing the influence of AC voltage amplitude on dendritic morphology (*f* = 80 Hz). (c) Microscopic images of dendritic formation at their completion time, grown under different frequencies from 10 to 950 Hz (voltage amplitude = 5 V). (d) Schematic illustration (top) and microscopic photos (bottom) showing the interconnectivity preference between output and input terminals based on the pulsing (events) frequency. Panels b, c, and d are adapted from reference 481 (CC BY 4.0). (e) Microscopic image of a reservoir network with 3 input and 3 output terminals. Panel adapted from reference. (f) Scheme of reservoir computing (top) based on the OMIEC dendritic network, and the diagrams showing nonlinear signal transformation (bottom) from input to output space. The top scheme is adapted from reference 488 (CC BY 4.0); the bottom diagrams are adapted from reference 485 (CC BY-NC 4.0).

Dendrite growth is typically achieved by electropolymerizing monomers in an electrolytic solution that immerses two thin terminal electrodes (e.g., gold wires), with AC bipolar pulses applied over a period of time ranging from seconds to minutes. Unlike DC input, the AC stimulus provides a controlled growth window during the oxidizing half-cycle, allowing polymerization to occur only at the electrode/OMIEC tips where the electric field is strongest. This results in directed, anisotropic growth into fibers, rather than uniform deposition of film. Under these conditions, electropolymerization initiates from both terminals, forming OMIEC branches that eventually merge in the middle. The addition of a third gate terminal creates an organic electrochemical synapse (OECS) whose channel is not only capable of evolving in thickness but also in topology. The detailed mechanism of OMIEC dendrite growth has been investigated and revealed as a synergistic effect between the tip effect (as discussed earlier) and polymer electrophoresis.^71^ In the case of EDOT polymerization, the previously deposited PEDOT fiber becomes doped and positively charged during the growing half-cycle, enabling it to electrophorese along the direction of the external electric field. This mechanism, combined with tip-preferred PEDOT growth, contributes to the dendrite-like morphology. This process requires sufficiently high conductivity in the OMIEC, which is why most studies have focused on PEDOT derivatives, while thiophene monomers failed to produce dendritic growth.^71^

The dendritic growth process is influenced by both electrical stimulation conditions and chemical factors. A higher amplitude of the AC stimulus results in an increased propagation rate, greater fractality, and a larger projected area within a given time frame^479,480^ (Figure 19b). However, this monotonic trend eventually saturates due to the limitation imposed by monomer diffusion rates. AC frequency plays another critical role in controlling morphology and directionality. Generally, lower frequencies produce thicker fibers, denser topologies, and a higher degree of branching.^481^ At higher frequencies, the dendritic network becomes less branchy and more linear. A transition from a fractal to a wire-like morphology has been observed as the stimulating frequency increases from 10 to 950 Hz (Figure 19c). The different diffusion behaviors of monomers and counterions are responsible for this morphological difference.^479,481^ However, opposite trends in network morphology versus AC frequency (i.e., higher frequency yields more branchy morphology) have been observed when using floating electrodes.^71^ The underlying reason for this discrepancy has not yet been thoroughly investigated. Additionally, the symmetry of the dendritic network between the two terminals can be adjusted by modulating the polarity of the AC input. Coupling a DC offset to the AC signal creates unequal growth windows on the two terminals, leading to an asymmetrical pattern in the OMIEC dendrites.^481^ Furthermore, the width of the fibers shows a positive correlation with the concentration of counterions,^482^ as a higher concentration increases the reaction rate.

While these growth parameters directly affect the dendritic network’s morphology and topology, they also influence its electrical properties, particularly the transconductance when used as the evolvable channel for OECSs. The capacitance of the OMIEC dendrites, which is proportional to their volume (i.e., the product of projected area and thickness), directly affects the OECT’s dynamics in doping/dedoping. Additionally, dendritic networks produced under higher frequencies exhibit higher conductivity and mobility,^479^ likely due to more organized PEDOT chains during the slower chain-growth process. Consequently, OECTs with dendritic channels of lower volumetric capacitance (i.e., lower area and thickness) and higher mobility lead to faster OECT response and more rapid memory decay for short-term plasticity.^483^ Another significant feature of OMIEC dendrite networks is that, after the OMIEC bisections make contact and form the channel, they can continue to grow and branch, emulating the reinforcement of neuronal synapses (learning-induced synaptogenesis). The resulting dendritic network exhibits nonvolatile conductance, providing LTP for neuromorphic applications. For instance, the learning curve (i.e., the rate of change in synaptic weight) of a dendritic OECS can be easily tuned by adjusting the growth frequency,^482^ with lower frequencies resulting in more effective growth and a faster increase in synaptic weight. LTD has also been demonstrated, attributed to the solubilization of the counterion dopant in the electrolyte solution, which reduces the doping level of PEDOT and thus its conductivity. Furthermore, in addition to using the dendritic network as the channel, it can also serve as an evolvable gate.^479^ Its growth through continued electropolymerization can alter the doping level in the channel and achieve LTP, similar to heterosynaptic interaction in biological neurons.

When multiple terminals are engaged in a shared electrolyte environment, a multinodal OMIEC dendritic device can be established to perform event-based sensing. This refers to the system’s ability to form multiple OMIEC connections with varying morphology and conductance, depending on the pulsing frequency (events per second) at each specific terminal. For instance, in a setup with one output terminal and multiple competing input terminals, the output will preferentially connect to the most electroactive input terminal with the highest synaptic weight (Figure 19d).^481^ By leveraging the structural plasticity of dendritic synapses, an artificial neural network (ANN) could be constructed from the bottom up, where dendritic connections are reinforced between terminals that fire closely in time (based on the mechanism of spike-timing-dependent plasticity, STDP), resulting in adaptive network connectivity reminiscent of Hebbian learning. Based on this principle, associative learning, such as Pavlovian conditioning,^482,484^ has been demonstrated. At the software level, a dendritic growth model has been simulated and applied to tasks such as the classification of electrophysiological signals.^484^

In parallel with the development of software-implemented dendritic ANNs, hardware-based ANNs have also been constructed by leveraging OMIEC dendrites to achieve reservoir computing,^485–488^ a brain-inspired ANN protocol known for its sparse connectivity and high energy efficiency. For instance, in a reservoir with 3 input and 3 output terminals (Figure 19e), the multichannel network forms a complex system where each OMIEC branch functions both as a synaptic channel and as a gate that influences the conductance of other channels.^485^ This inherent stochasticity enables the reservoir to project input signals nonlinearly into the output layer (Figure 19f), allowing for the linear classification of time-dependent data, such as electrocardiogram (ECG) waves with different abnormalities, which cannot be linearly separated in the input space. Given the biocompatibility and ion permeability of OMIEC, as well as the in-liquid operation of the device, this reservoir computing platform holds great promise for real-time, in vivo detection and classification of abnormal vital signals in physiological environments.

So far, only PEDOT derivatives, including PEDOT:PSS and PEDOT:PF_6_, have been predominantly employed for dendrite growth. EDOT derivatives with alkyl side chains exhibit unique morphology and microscopic structures that differ from those polymerized from EDOT;^71^ however, the relationship between chemical structure and morphology in this context remains unclear and requires further investigation. Additionally, the production of dendritic networks using other monomer families has not yet been explored, leaving a significant gap in the field. Reversibility in PEDOT-based dendrites also presents a significant challenge. While overoxidation has been proposed as a LTD strategy for other polymers like PETE-S,^42^ this approach may not be suitable for PEDOT due to its greater electrochemical stability. Future advancements will require the discovery of suitable OMIEC materials capable of dendritic growth, as well as the ability to easily erase and rewrite, so as to develop highly versatile ANNs with structural plasticity.

## 4 Current Challenges and Future Directions

One of the most transformative advantages of electropolymerized OMIECs is their ability to reduce interfacial impedance, a crucial factor for bioelectronics. This advancement has led to miniaturized, highly sensitive electrodes capable of recording and stimulating electrogenic cells with unprecedented precision. As bioelectronics evolves toward more seamless integration with living systems, OMIECs will play a key role in bridging the communication gap between biological and electronic components, enabling higher density electrode arrays for neural prosthetics and *in vivo* biosensing. The introduction of water-soluble monomers that can be oxidized at potentials that do not cause significant tissue damage has stimulated a renewed interest in *in vivo* electropolymerization.^16^ Several challenges remain, however, before this technology can surpass the sophistication of a low precision stimulating electrode. If electropolymerized OMIEC materials are to be used as wires to contact a biological target region, biocompatible strategies need to be developed for improving OMIEC conductivity and for insulating the wires from ionic currents that originate from outside the area of interest. Lastly, improving the durability of electropolymerized materials under physiological conditions is critical to ensuring their viability for long-term biomedical applications. Studies of *in vivo* OMIEC degradation, especially studies of how the immune system interacts with OMIEC materials may inform approaches to modulating the immune response and extend or shorten the lifetime of OMIEC-based implants, depending on need.

There are several interesting opportunities to adapt past research on electropolymerized OMIECs for in *vivo applications*. First, as Nishizawa et al. showed over 30 years ago, electropolymerized OMIEC polymer can be directed to spread along a path that is functionalized with a material that strongly interacts with the monomer.^246^ This capability highlights the potential for next generation electrode implants to be selectively targeted to specific tissue types based on their cell surface properties, membrane protein composition, or disease state. Achieving this vision will require a comprehensive understanding of the surface properties of cells and tissues. A much more recent result by Kuhn et al., which demonstrates asymmetric functionalization of microscale particles by wireless electropolymerization, illustrates another interesting direction for next generation *in vivo* electronics.^320^ While bipolar electropolymerization is not feasible to carry out *in vivo* due to the requirements for low ionic strength and high driving voltages, the functionalized microparticles, in conjunction with targeting and anchoring molecules, provide for an incredibly diverse and sophisticated set of building blocks for to design multicomponent electronics that self-assemble inside the body.

Electropolymerized OMIECs have also revealed new avenues in the field of neuromorphic engineering. Evolvable organic electrochemical synapses exhibit programmable analog conductance and mimic the key functions of biological neural networks. such as synaptic plasticity and spike-timing-dependent learning. This emerging field has the potential to revolutionize computing, providing energy-efficient, brain-inspired alternatives to conventional hardware.

Despite these promising developments within this field, several challenges remain. Precise control of polymer morphology, doping levels, and long-term stability remains an active area of research, particularly in dendritic OMIEC networks. Most importantly, for this field to advance beyond simple demonstrators, it is critical to develop large-scale integrated circuits comprising neurons with many-to-many conductivity through synapses. For large-scale integration to be feasible, it is necessary to develop a systematic approach for electrolyte handling, which can be achieved using, for example, microfluidics or patterned electrolytes.

Microfluidic solutions are especially well-suited for *in situ* training of neuromorphic circuits, as they provide an extra level of control over electrolyte composition, greatly facilitating bidirectional modulation of synaptic weight at the device level. This is because the synaptic weight can be up-regulated in the presence of monomer through oxidative electropolymerization or down-regulated in the absence of monomer through overoxidation of the polymer material. Since overoxidized material becomes brittle, it can be easily removed by fluid flow, providing for fully reversible synaptic weight modulation.

At even larger scales, microfluidics become untenable due to the need for electrolyte separation in high-density passive matrix synaptic arrays. In this case, patterned electrolytes would offer a better solution. However, implementing this approach introduces challenges related to the lack of weight reversibility and necessitates additional engineering solutions to achieve reversible weight characteristics.

Several ongoing research efforts show promise for realizing the potential of evolvable neuromorphic processors. Input-directed interconnection in three-dimensional space^484,489^ as well as the development of vertical devices^39^ are both necessary to achieve the brain’s high-density 3D integration that is the object of great admiration by circuit designers. Looking ahead, it would also be interesting to expand the principles of evolvable electronics to other neuromorphic circuit elements, like neurons. Dynamic changes to capacitive elements within some neuron models can alter the neuron’s sensitivity to all synaptic inputs without affecting the synaptic weights, introducing additional degrees of freedom to neuromorphic systems.

From a materials perspective, future research of electropolymerized OMIECs should prioritize: 1) developing novel OMIECs with enhanced stability, tunable electronic properties, and improved biocompatibility; 2) refining electropolymerization techniques to achieve precise control over polymer growth, enabling the fabrication of complex architectures; and 3) bridging the gap between lab-scale demonstrations and large-scale manufacturing to facilitate the commercialization of OMIEC-based technologies. As electropolymerization continues to push the boundaries of organic electronics, it holds great promise for biointerfaces, sensors, and neuromorphic computing. By addressing these challenges and embracing interdisciplinary collaboration, this field will remain at the forefront of next-generation electronic and bioelectronic innovations.

## References

[1] Greene J. E. (2014). Tracing
the 5000-year recorded history of inorganic thin films from ∼
3000 BC to the early 1900s AD. Applied Physics
Reviews.

[2] Hunt L. B. (1973). The early
history of gold plating. Gold Bulletin.

[3] Letheby H. (1862). XXIX.—On
the production of a blue substance by the electrolysis of sulphate
of aniline. Journal of the Chemical Society.

[4] Shirakawa H., Louis E. J., MacDiarmid A. G., Chiang C. K., Heeger A. J. (1977). Synthesis
of electrically conducting organic polymers: halogen derivatives of
polyacetylene, (CH). J. Chem. Soc., Chem. Commun..

[5] Waltman R. J., Bargon J. (1986). Electrically conducting
polymers: a review of the electropolymerization
reaction, of the effects of chemical structure on polymer film properties,
and of applications towards technology. Can.
J. Chem..

[6] Heinze J., Frontana-Uribe B. A., Ludwigs S. (2010). Electrochemistry of Conducting Polymers—Persistent
Models and New Concepts. Chem. Rev..

[7] Otero T. F., Martinez J. G. (2016). Electro-chemo-biomimetics
from conducting polymers:
fundamentals, materials, properties and devices. J. Mater. Chem. B.

[8] Kim N.-J., Kwon J.-H., Kim M. (2013). Highly Oriented
Self-Assembly of
Conducting Polymer Chains: Extended-Chain Crystallization during Long-Range
Polymerization. J. Phys. Chem. C.

[9] O’Neil K. D., Shaw B., Semenikhin O. A. (2007). On the Origin of Mesoscopic Inhomogeneity
of Conducting Polymers. J. Phys. Chem. B.

[10] Ma H., Chen Y., Li X., Li B. (2021). Advanced Applications
and Challenges of Electropolymerized Conjugated Microporous Polymer
Films. Adv. Funct. Mater..

[11] Tan S. T. M., Gumyusenge A., Quill T. J., LeCroy G. S., Bonacchini G. E., Denti I., Salleo A. (2022). Mixed Ionic-Electronic Conduction,
a Multifunctional Property in Organic Conductors. Adv. Mater..

[12] Paulsen B. D., Tybrandt K., Stavrinidou E., Rivnay J. (2020). Organic mixed ionic-electronic
conductors. Nat. Mater..

[13] Kim H., Won Y., Song H. W., Kwon Y., Jun M., Oh J. H. (2024). Organic
Mixed Ionic-Electronic Conductors for Bioelectronic Sensors: Materials
and Operation Mechanisms. Adv. Sci. (Weinh).

[14] Gkoupidenis P., Zhang Y., Kleemann H., Ling H., Santoro F., Fabiano S., Salleo A., van de Burgt Y. (2024). Organic mixed
conductors for bioinspired electronics. Nat.
Rev. Mater..

[15] Strakosas X., Biesmans H., Abrahamsson T., Hellman K., Ejneby M. S., Donahue M. J., Ekström P., Ek F., Savvakis M., Hjort M. (2023). Metabolite-induced in
vivo fabrication of substrate-free
organic bioelectronics. Science.

[16] Hjort M., Mousa A. H., Bliman D., Shameem M. A., Hellman K., Yadav A. S., Ekström P., Ek F., Olsson R. (2023). In situ assembly
of bioresorbable organic bioelectronics in the brain. Nature Comm.

[17] Richardson-Burns S. M., Hendricks J. L., Martin D. C. (2007). Electrochemical
polymerization of
conducting polymers in living neural tissue. Journal of Neural Engineering.

[18] Richardson-Burns S. M., Hendricks J. L., Foster B., Povlich L. K., Kim D.-H., Martin D. C. (2007). Polymerization
of the conducting polymer poly­(3,4-ethylenedioxythiophene)
(PEDOT) around living neural cells. Biomaterials.

[19] Yun M., Myung N. V., Vasquez R. P., Wang J., Monbouquette H. (2003). Nanowire growth
for sensor arrays. Proc.SPIE.

[20] Gerasimov J. Y., Tu D., Hitaishi V., Harikesh P. C., Yang C. Y., Abrahamsson T., Rad M., Donahue M. J., Ejneby M. S., Berggren M. (2023). A Biologically
Interfaced Evolvable Organic Pattern Classifier. Advanced Science.

[21] Riess I. (2000). Polymeric
mixed ionic electronic conductors. Solid State
Ion.

[22] Flagg L. Q., Giridharagopal R., Guo J., Ginger D. S. (2018). Anion-Dependent
Doping and Charge Transport in Organic Electrochemical Transistors. Chem. Mater..

[23] Cavassin P., Holzer I., Tsokkou D., Bardagot O., Réhault J., Banerji N. (2023). Electrochemical Doping
in Ordered and Disordered Domains
of Organic Mixed Ionic-Electronic Conductors. Adv. Mater..

[24] Torricelli F., Adrahtas D. Z., Bao Z., Berggren M., Biscarini F., Bonfiglio A., Bortolotti C. A., Frisbie C. D., Macchia E., Malliaras G. G. (2021). Electrolyte-gated transistors for enhanced
performance bioelectronics. Nature Reviews Methods
Primers.

[25] Sun H., Gerasimov J., Berggren M., Fabiano S. (2018). n-Type organic electrochemical
transistors: materials and challenges. Journal
of Materials Chemistry C.

[26] Sahalianov I., Singh S. K., Tybrandt K., Berggren M., Zozoulenko I. (2019). The intrinsic
volumetric capacitance of conducting polymers: pseudo-capacitors or
double-layer supercapacitors?. RSC Adv..

[27] Scotto J., Marmisollé W. A., Posadas D. (2019). About the capacitive currents in
conducting polymers: the case of polyaniline. J. Solid State Electrochem..

[28] Kim K., Park J., Lee J., Suh S., Kim W. (2023). Ultrafast
PEDOT:PSS/H2SO4 Electrical Double Layer Capacitors: Comparison with
Polyaniline Pseudocapacitors. ChemSusChem.

[29] Strakosas, X. ; Bongo, M. ; Owens, R. M. The organic electrochemical transistor for biological applications. J. Appl. Polym. Sci. 2015, 132 (15), 10.1002/app.41735.

[30] Huang Y., Hu Z., Zhu S., Fang B. (2025). Fibre-based organic electrochemical
transistors: principle, evaluation, and application. npj Flexible Electronics.

[31] Marks A., Griggs S., Gasparini N., Moser M. (2022). Organic Electrochemical
Transistors: An Emerging Technology for Biosensing. Advanced Materials Interfaces.

[32] Harikesh P. C., Tu D., Fabiano S. (2024). Organic electrochemical
neurons for neuromorphic perception. Nature
Electronics.

[33] Paulsen B. D., Fabiano S., Rivnay J. (2021). Mixed Ionic-Electronic
Transport
in Polymers. Annu. Rev. Mater. Res..

[34] Bruno, U. ; Mariano, A. ; Rana, D. ; Gemmeke, T. ; Musall, S. ; Santoro, F. From neuromorphic to neurohybrid: transition from the emulation to the integration of neuronal networks. Neuromorphic Computing and Engineering 2023, 3 (2), 023002. 10.1088/2634-4386/acc683

[35] Boufidis D., Garg R., Angelopoulos E., Cullen D. K., Vitale F. (2025). Bio-inspired
electronics: Soft, biohybrid, and “living” neural interfaces. Nature Comm.

[36] Zhao C., Park J., Root S. E., Bao Z. (2024). Skin-inspired
soft
bioelectronic materials, devices and systems. Nature Reviews Bioengineering.

[37] Elnathan R., Barbato M. G., Guo X., Mariano A., Wang Z., Santoro F., Shi P., Voelcker N. H., Xie X., Young J. L. (2022). Biointerface
design for vertical nanoprobes. Nat. Rev. Mater..

[38] Ohayon D., Druet V., Inal S. (2023). A guide for the characterization
of organic electrochemical transistors and channel materials. Chem. Soc. Rev..

[39] Gryszel, M. ; Byun, D. ; Burtscher, B. ; Abrahamsson, T. ; Brodsky, J. ; Simon, D. T. ; Berggren, M. ; Glowacki, E. D. ; Strakosas, X. ; Donahue, M. J. Vertical organic electrochemical transistor platforms for efficient electropolymerization of thiophene based oligomers. Journal of Materials Chemistry C 2024, 12, 5339. 10.1039/D3TC04730J 38645749 PMC11025323

[40] Keene S. T., Laulainen J. E. M., Pandya R., Moser M., Schnedermann C., Midgley P. A., McCulloch I., Rao A., Malliaras G. G. (2023). Hole-limited
electrochemical doping in conjugated polymers. Nat. Mater..

[41] Hassarati R. T., Dueck W. F., Tasche C., Carter P. M., Poole-Warren L. A., Green R. A. (2014). Improving Cochlear Implant Properties
Through Conductive
Hydrogel Coatings. IEEE Transactions on Neural
Systems and Rehabilitation Engineering.

[42] Gerasimov J. Y., Gabrielsson R., Forchheimer R., Stavrinidou E., Simon D. T., Berggren M., Fabiano S. (2019). An evolvable organic
electrochemical transistor for neuromorphic applications. Adv. Sci..

[43] Harikesh P. C., Yang C.-Y., Tu D., Gerasimov J. Y., Dar A. M., Armada-Moreira A., Massetti M., Kroon R., Bliman D., Olsson R. (2022). Organic electrochemical
neurons and synapses with ion mediated spiking. Nature Comm.

[44] Berggren M., Głowacki E. D., Simon D. T., Stavrinidou E., Tybrandt K. (2022). In Vivo Organic Bioelectronics
for Neuromodulation. Chem. Rev..

[45] Dimov I. B., Moser M., Malliaras G. G., McCulloch I. (2022). Semiconducting
Polymers for Neural Applications. Chem. Rev..

[46] Tseng H., Weissbach A., Kucinski J., Solgi A., Nair R., Bongartz L. M., Ciccone G., Cucchi M., Leo K., Kleemann H. (2023). Threshold Voltage Control in Dual-Gate Organic Electrochemical
Transistors. Advanced Materials Interfaces.

[47] Tan S. T. M., Lee G., Denti I., LeCroy G., Rozylowicz K., Marks A., Griggs S., McCulloch I., Giovannitti A., Salleo A. (2022). Tuning Organic Electrochemical
Transistor
Threshold Voltage using Chemically Doped Polymer Gates. Adv. Mater..

[48] Shim J.-S., Rogers J. A., Kang S.-K. (2021). Physically transient electronic materials
and devices. Materials Science and Engineering:
R: Reports.

[49] Wang X.-S., Tang H.-P., Li X.-D., Hua X. (2009). Investigations on the
Mechanical Properties of Conducting Polymer Coating-Substrate Structures
and Their Influencing Factors. International
Journal of Molecular Sciences.

[50] Holze, R. Overoxidation of Intrinsically Conducting Polymers. Polymers 2022, 14, 1584. 10.3390/polym14081584 35458334 PMC9027932

[51] Gueskine, V. ; Vagin, M. ; Berggren, M. ; Crispin, X. ; Zozoulenko, I. Oxygen reduction reaction at conducting polymer electrodes in a wider context: Insights from modelling concerning outer and inner sphere mechanisms. Electrochemical Science Advances 2023, 3 (2), 10.1002/elsa.202100191.

[52] Zhang S., Ding P., Ruoko T.-P., Wu R., Stoeckel M.-A., Massetti M., Liu T., Vagin M., Meli D., Kroon R. (2023). Toward Stable p-Type Thiophene-Based Organic Electrochemical
Transistors. Adv. Funct. Mater..

[53] Yalcin D., Bamford S., Espiritu M., Rigopoulos N., Martinez-Botella I., Alexander D., Gozukara Y., Greaves M., Bruton E. A., Kinlen P. J. (2023). New insight into degradation
mechanisms of conductive and thermally resistant polyaniline films. Polym. Degrad. Stab..

[54] Vitoratos E., Sakkopoulos S., Dalas E., Paliatsas N., Karageorgopoulos D., Petraki F., Kennou S., Choulis S. A. (2009). Thermal
degradation mechanisms of PEDOT:PSS. Org. Electron..

[55] Zhu D., Zhu Z., Ma X., Xu J., Zhou W. (2023). Substitution effects
of cyano group on the electropolymerization, thermal stability, morphology,
and supercapacitor performance of indole based polymers. Synth. Met..

[56] Liu Y.-C., Tsai C.-J. (2003). Enhancements
in Conductivity and Thermal and Conductive
Stabilities of Electropolymerized Polypyrrole with Caprolactam-Modified
Clay. Chem. Mater..

[57] Gong H., Xiang J., Xu L., Song X., Dong Z., Peng R., Liu Z. (2015). Stimulation
of immune systems by
conjugated polymers and their potential as an alternative vaccine
adjuvant. Nanoscale.

[58] Qian S., Lin H.-A., Pan Q., Zhang S., Zhang Y., Geng Z., Wu Q., He Y., Zhu B. (2023). Chemically
revised conducting polymers with inflammation resistance for intimate
bioelectronic electrocoupling. Bioactive Materials.

[59] Cihaner A., Önal A. M. (2007). Electrochemical
synthesis of poly­(3-bromo-4-methoxythiophene)
and its device application. J. Electroanal.
Chem..

[60] Ratcliff E. L., Jenkins J. L., Nebesny K., Armstrong N. R. (2008). Electrodeposited,
“Textured” Poly­(3-hexyl-thiophene) (e-P3HT) Films for
Photovoltaic Applications. Chem. Mater..

[61] Lankinen E., Sundholm G., Talonen P., Granö H., Sundholm F. (1999). Synthesis, electropolymerization
and electrochemical
characterization of some new acrylate substituted thiophene derivatives. J. Electroanal. Chem..

[62] Swathi M., Chetri R., Ahipa T. N. (2025). Electropolymerization
Strategies
on Thiophene Derivatives: An Overview. ChemistrySelect.

[63] Kassahun G. S., Farias E. D., Benizri S., Mortier C., Gaubert A., Salinas G., Garrigue P., Kuhn A., Zigah D., Barthélémy P. (2022). Electropolymerizable Thiophene-Oligonucleotides
for Electrode Functionalization. ACS Appl. Mater.
Interfaces.

[64] Du X., Wang Z. (2003). Effects of polymerization potential on the properties of electrosynthesized
PEDOT films. Electrochim. Acta.

[65] Mantione D., Istif E., Dufil G., Vallan L., Parker D., Brochon C., Cloutet E., Hadziioannou G., Berggren M., Stavrinidou E., Pavlopoulou E. (2020). Thiophene-Based
Trimers for In Vivo Electronic Functionalization of Tissues. ACS Applied Electronic Materials.

[66] Dietrich M., Heinze J., Heywang G., Jonas F. (1994). Electrochemical and
spectroscopic characterization of polyalkylenedioxythiophenes. J. Electroanal. Chem..

[67] Wang R., Li J., Gao L., Yu J. (2022). One-step electropolymerized thieno­[3,2-b]­thiophene-based
bifunctional electrode with controlled color conversion for electrochromic
energy storage application. Chemical Engineering
Journal.

[68] Poverenov E., Li M., Bitler A., Bendikov M. (2010). Major Effect of Electropolymerization
Solvent on Morphology and Electrochromic Properties of PEDOT Films. Chem. Mater..

[69] Chen H., Wang W., Zhu J., Han Y., Liu J. (2022). Electropolymerization
of D-A type EDOT-based monomers consisting of camphor substituted
quinoxaline unit for electrochromism with enhanced performance. Polymer.

[70] Mantione D., Del Agua I., Sanchez-Sanchez A., Mecerreyes D. (2017). Poly­(3,4-ethylenedioxythiophene)
(PEDOT) Derivatives: Innovative Conductive Polymers for Bioelectronics. Polymers.

[71] Koizumi Y., Shida N., Ohira M., Nishiyama H., Tomita I., Inagi S. (2016). Electropolymerization on wireless
electrodes towards conducting polymer microfibre networks. Nature Comm.

[72] Kumar A., Welsh D. M., Morvant M. C., Piroux F., Abboud K. A., Reynolds J. R. (1998). Conducting Poly­(3,4-alkylenedioxythiophene) Derivatives
as Fast Electrochromics with High-Contrast Ratios. Chem. Mater..

[73] Darmanin T., Guittard F. (2011). Superhydrophobic Fiber Mats by Electrodeposition of
Fluorinated Poly­(3,4-ethyleneoxythiathiophene). J. Am. Chem. Soc..

[74] Taleb S., Darmanin T., Guittard F. (2014). Superhydrophobic
conducting polymers
with switchable water and oil repellency by voltage and ion exchange. RSC Adv..

[75] Ghazal M., Susloparova A., Lefebvre C., Daher Mansour M., Ghodhbane N., Melot A., Scholaert C., Guérin D., Janel S., Barois N. (2023). Electropolymerization
processing of side-chain engineered EDOT for high performance microelectrode
arrays. Biosens. Bioelectron..

[76] Gerasimov J., Halder A., Mousa A. H., Ghosh S., Harikesh P. C., Abrahamsson T., Bliman D., Strandberg J., Massetti M., Zozoulenko I. (2022). Rational materials design
for in operando electropolymerization of evolvable organic electrochemical
transistors. Adv. Funct. Mater..

[77] Salinas G., Del-Oso J.-A., Espinoza-Montero P.-J., Heinze J., Frontana-Uribe B. A. (2018). Electrochemical
polymerization, characterization and in-situ conductivity studies
of poly-3,4-ortho-xylendioxythiophene (PXDOT). Synth. Met..

[78] Blanchard P., Cappon A., Levillain E., Nicolas Y., Frère P., Roncali J. (2002). Thieno­[3,4-b]-1,4-oxathiane: An Unsymmetrical Sulfur
Analogue of 3,4-Ethylenedioxythiophene (EDOT) as a Building Block
for Linear π-Conjugated Systems. Org.
Lett..

[79] Roncali J. (1992). Conjugated
poly­(thiophenes): synthesis, functionalization, and applications. Chem. Rev..

[80] Feng Z., Mo D., Wang Z., Zhen S., Xu J., Lu B., Ming S., Lin K., Xiong J. (2015). Low-potential electrosynthesis
of a novel nitrogen analog of PEDOT in an ionic liquid and its optoelectronic
properties. Electrochim. Acta.

[81] Ermiş E., Yiğit D., Güllü M. (2013). Synthesis of poly­(N-alkyl-3,4-dihydrothieno­[3,4-b]­[1,4]­oxazine)
derivatives and investigation of their supercapacitive performances
for charge storage applications. Electrochim.
Acta.

[82] Yadav P., Patra A. (2020). Recent advances in poly­(3,4-ethylenedioxyselenophene) and related
polymers. Polym. Chem..

[83] Patra A., Bendikov M., Chand S. (2014). Poly­(3,4-ethylenedioxyselenophene)
and Its Derivatives: Novel Organic Electronic Materials. Acc. Chem. Res..

[84] Kaloni T. P., Schreckenbach G., Freund M. S. (2016). Band gap modulation in polythiophene
and polypyrrole-based systems. Sci. Rep..

[85] Zade S. S., Bendikov M. (2006). From Oligomers to Polymer:
Convergence in the HOMO-LUMO
Gaps of Conjugated Oligomers. Org. Lett..

[86] Patra A., Wijsboom Y. H., Zade S. S., Li M., Sheynin Y., Leitus G., Bendikov M. (2008). Poly­(3,4-ethylenedioxyselenophene). J. Am. Chem. Soc..

[87] Ertan S., Cihaner A. (2018). Designing a Solution
Processable Poly­(3,4-ethylenedioxyselenophene)
Analogue. Macromolecules.

[88] Scheuble M., Goll M., Ludwigs S. (2015). Branched Terthiophenes
in Organic
Electronics: From Small Molecules to Polymers. Macromol. Rapid Commun..

[89] Yassin A., Mallet R., Leriche P., Roncali J. (2014). Production of Nanostructured
Conjugated Polymers by Electropolymerization of Tailored Tetrahedral
Precursors. ChemElectroChem..

[90] Zhang Y., Li R., Chang L., Ma Y., Hou Y., Niu H. (2022). Electropolymerization
of Thiophene-Based Monomers with Different Spatial Structures: The
Impact of Monomer Structure on Electrochromic Properties. Macromol. Chem. Phys..

[91] Sabouraud G., Sadki S., Brodie N. (2000). The mechanisms of pyrrole electropolymerization. Chem. Soc. Rev..

[92] Dubal D. P., Lee S. H., Kim J. G., Kim W. B., Lokhande C. D. (2012). Porous
polypyrrole clusters prepared by electropolymerization for a high
performance supercapacitor. J. Mater. Chem..

[93] Korent A., Žagar Soderžnik K., Šturm S., Žužek Rožman K. (2020). A Correlative
Study of Polyaniline
Electropolymerization and its Electrochromic Behavior. J. Electrochem. Soc..

[94] Kulandaivalu S., Zainal Z., Sulaiman Y. (2016). Influence of Monomer
Concentration
on the Morphologies and Electrochemical Properties of PEDOT, PANI,
and PPy Prepared from Aqueous Solution. International
Journal of Polymer Science.

[95] Bruckenstein S., Sharkey J. W. (1988). Interpretation of polyazulene electropolymerization
considering faradaic current efficiency and capacitive current effects
during the growth and redox. Journal of Electroanalytical
Chemistry and Interfacial Electrochemistry.

[96] Agrawal V., Shahjad, Bhardwaj D., Bhargav R., Sharma G. D., Bhardwaj R. K., Patra A., Chand S. (2016). Morphology
and Doping Level of Electropolymerized Biselenophene-Flanked
3,4- Ethylenedioxythiophene Polymer: Effect of Solvents and Electrolytes. Electrochim. Acta.

[97] Saraji M., Bagheri A. (1998). Electropolymerization
of indole and study of electrochemical
behavior of the polymer in aqueous solutions. Synth. Met..

[98] Hsiao S.-H., Lin S.-W. (2016). Electrochemical synthesis of electrochromic
polycarbazole
films from N-phenyl-3,6-bis­(N-carbazolyl)­carbazoles. Polym. Chem..

[99] Guo D., Li X., Ming F., Zhou Z., Liu H., Hedhili M. N., Tung V., Alshareef H. N., Li Y., Lai Z. (2020). Electropolymerization
growth of an ultrathin, compact, conductive and microporous (UCCM)
polycarbazole membrane for high energy Li-S batteries. Nano Energy.

[100] Shao S., Shi J., Murtaza I., Xu P., He Y., Ghosh S., Zhu X., Perepichka I. F., Meng H. (2017). Exploring the electrochromic
properties of poly­(thieno­[3,2-b]­thiophene)­s
decorated with electron-deficient side groups. Polym. Chem..

[101] Xue Y., Xue Z., Zhang W., Zhang W., Chen S., Lin K., Xu J. (2018). Enhanced electrochromic performances of Polythieno­[3,2-b]­thiophene
with multicolor conversion via embedding EDOT segment. Polymer.

[102] Ramos Chagas G., Akbari R., Godeau G., Mohammadizadeh M., Guittard F., Darmanin T. (2017). Electrodeposited Poly­(thieno­[3,2-b]­thiophene)
Films for the Templateless Formation of Porous Structures by Galvanostatic
and Pulse Deposition. ChemPlusChem..

[103] Bronstein H., Chen Z., Ashraf R. S., Zhang W., Du J., Durrant J. R., Shakya Tuladhar P., Song K., Watkins S. E., Geerts Y. (2011). Thieno­[3,2-b]­thiophene-Diketopyrrolopyrrole-Containing
Polymers for High-Performance Organic Field-Effect Transistors and
Organic Photovoltaic Devices. J. Am. Chem. Soc..

[104] Xia C., Advincula R. C., Baba A., Knoll W. (2004). Electrochemical Patterning
of a Polyfluorene Precursor Polymer from a Microcontact Printed (μCP)
Monolayer. Chem. Mater..

[105] Guo F., Zhang M., Zhao S., Hu L., Xiao B., Ying L., Yang R. (2022). Efficient polyfluorene
derivatives
for blue light-emitting diodes enabled by tuning conjugation length
of bulky chromophores. Dyes Pigm..

[106] Liu B., Yu W.-L., Lai Y.-H., Huang W. (2001). Blue-Light-Emitting
Fluorene-Based Polymers with Tunable Electronic Properties. Chem. Mater..

[107] Adams R. N. (1969). Anodic oxidation pathways of aromatic
hydrocarbons
and amines. Acc. Chem. Res..

[108] Ambrose J. F., Nelson R. F. (1968). Anodic Oxidation
Pathways of Carbazoles:
I. Carbazole and N-Substituted Derivatives. J. Electrochem. Soc..

[109] Cauquis G., Genies M. (1968). Electrochemical oxidation of some
Pentaphenylr pyrrole in organic medium. Appropriate radical cations. Chemisches Zentralblatt.

[110] Audebert P., Catel J. M., Le Coustumer G., Duchenet V., Hapiot P. (1998). Electrochemistry and Polymerization
Mechanisms of Thiophene-Pyrrole-Thiophene Oligomers and Terthiophenes.
Experimental and Theoretical Modeling Studies. J. Phys. Chem. B.

[111] Audebert P., Hapiot P. (1995). Fast electrochemical
studies of the
polymerization mechanisms of pyrroles and thiophenes. Identification
of the first steps. Existence of π-dimers in solution. Synth. Met..

[112] Zettersten C., Sjoberg P. J., Nyholm L. (2009). Oxidation of 4-chloroaniline
studied by on-line electrochemistry electrospray ionization mass spectrometry. Anal. Chem..

[113] Alberola A., Farley R. D., Humphrey S. M., McManus G. D., Murphy D. M., Rawson J. M. (2005). EPR studies on the
thiophenodithiazolyl
radical, C4H2S3N. Dalton Trans.

[114] Kirste B., Tian P., Kossmehl G., Engelmann G., Jugelt W. (1995). EPR study of Bi-, Ter- and quaterthiophene
radical
cations. Magn. Reson. Chem..

[115] Rajca A., Shu C., Zhang H., Zhang S., Wang H., Rajca S. (2021). Thiophene-Based Double
Helices: Radical
Cations with SOMO-HOMO Energy Level Inversion. Photochem. Photobiol..

[116] Moise G., Tejerina L., Rickhaus M., Anderson H. L., Timmel C. R. (2019). Spin Delocalization in the Radical
Cations of Porphyrin
Molecular Wires: A New Perspective on EPR Approaches. J. Phys. Chem. Lett..

[117] Roessler M. M., Salvadori E. (2018). Principles
and applications of EPR
spectroscopy in the chemical sciences. Chem.
Soc. Rev..

[118] Streeter I., Wain A. J., Thompson M., Compton R. G. (2005). In situ
electrochemical ESR and voltammetric studies on the anodic oxidation
of para-haloanilines in acetonitrile. J. Phys.
Chem. B.

[119] Eberson, L. ; Hartshorn, M. P. ; Persson, O. 1,1,1,3,3,3-Hexafluoropropan-2-ol as a solvent for the generation of highly persistent radical cations. Journal of the Chemical Society, Perkin Transactions 2 1995, (9), 1735. 10.1039/p29950001735

[120] Tabakovic, I. ; Maki, T. ; Miller, L. L. ; Yu, Y. Persistent thiophene cation radicals. Chem. Commun. 1996, (16), 1911. 10.1039/cc9960001911

[121] Aleman C., Brillas E., Davies A. G., Fajari L., Giro D., Julia L., Perez J. J., Rius J. (1993). 2,2’-Bithienyl
derivatives: EPR investigation of their radical ions in solution,
electrochemical properties, and crystal structure. Journal of Organic Chemistry.

[122] Bäuerle P. (1992). End-capped oligothiophenes—new
model compounds
for polythiophenes. Adv. Mater..

[123] Waltman R. J., Bargon J. (1984). Reactivity/structure
correlations
for the electropolymerization of pyrrole: An INDO/CNDO study of the
reactive sites of oligomeric radical cations. Tetrahedron.

[124] Takakubo M. (1989). Molecular orbital study of the initial reaction paths
in the electrochemical polymerization of aniline. Synth. Met..

[125] Lacroix J. C., Harvard G., Aaron J. J., Taha-Bouamri K., Lacaze P. C. (1997). Modeling the growth and molecular structure of conductive
polymers: Application to poly­(dialkoxybenzene)­s. Structural Chemistry.

[126] Fréchette M., Belletete M., Bergeron J. Y., Durocher G., Leclerc M. (1997). Monomer reactivity
vs regioregularity in polythiophene
derivatives: A joint synthetic and theoretical investigation. Synth. Met..

[127] Yurtsever M., Yurtsever E. (2004). Density functional
theory study of
the electrochemical oligomerization of thiophene: transition states
for radical-radical and radical-neutral pathways. Polymer.

[128] Pang, S.-K. Comprehensive study of polymerization of pyrrole: A theoretical approach. J. Electroanal. Chem. 2020, 859, 113886. 10.1016/j.jelechem.2020.113886

[129] D’Aprano G., Proynov E., Lebœuf M., Leclerc M., Salahub D. R. (1996). Spin Densities and Polymerizabilities
of Aniline Derivatives Deduced from Density Functional Calculations. J. Am. Chem. Soc..

[130] Heth C. L., Tallman D. E., Rasmussen S. C. (2010). Electrochemical
study of 3-(N-alkylamino)­thiophenes: experimental and theoretical
insights into a unique mechanism of oxidative polymerization. J. Phys. Chem. B.

[131] Smith, J. R. ; Cox, P. A. ; Campbell, S. A. ; Ratcliffe, N. M. Application of density functional theory in the synthesis of electroactive polymers. Journal of the Chemical Society, Faraday Transactions 1995, 91 (15), 2331. 10.1039/ft9959102331

[132] Nalawade P., Naumov S., Kapoor S. (2015). Hidden chemistry
of
substituted aniline radical cations in water: a mechanistic study. J. Phys. Org. Chem..

[133] Sah C., Yadav A. K., Venkataramani S. (2018). Deciphering
Stability of Five-Membered
Heterocyclic Radicals: Balancing Act Between Delocalization and Ring
Strain. J. Phys. Chem. A.

[134] Davies, A. G. ; Julia, L. ; Yazdi, S. N. An electron spin resonance study of the radical cations of pyrroles, furans, and thiophenes in liquid solution. Journal of the Chemical Society, Perkin Transactions 2 1989, (3), 239. 10.1039/p29890000239

[135] Rao, D. N. R. ; Symons, M. C. R. Unstable intermediates. Part 205. Radical cations of pyrrole, furan, and thiophen derivatives: an electron spin resonance study. Journal of the Chemical Society, Perkin Transactions 2 1983, (2), 135. 10.1039/p29830000135

[136] Avila, D. V. ; Davies, A. G. Electron paramagnetic resonance spectroscopy of hetero[5]­annulenes. Journal of the Chemical Society, Faraday Transactions 1990, 86 (19), 3243. 10.1039/ft9908603243

[137] Emmi S. S., D’Angelantonio M., Poggi G., Beggiato G., Camaioni N., Geri A., Martelli A., Pietropaolo D., Zotti G. (1998). The spectral characterization
of
thiophene radical cation generated by pulse radiolysis. Res. Chem. Intermed..

[138] Ishigaki A., Koizumi H. (2012). Radiation-induced polymerization
of 3-octylthiophene. Radiat. Phys. Chem..

[139] Conner N. R., Holubowitch N. E. (2025). Shining Light on Electropolymerization:
Spectroelectrochemistry Reveals Electrochemical-Chemical Dynamics
in the Diffusion Layer and Their Impact on Polymer Film Quality. ACS Electrochemistry.

[140] Andrieux C. P., Audebert P., Hapiot P., Saveant J. M. (1990). Observation
of the cation radicals of pyrrole and of some substituted pyrroles
in fast-scan cyclic voltammetry. Standard potentials and lifetimes. J. Am. Chem. Soc..

[141] Andrieux C. P., Hapiot P., Audebert P., Guyard L., Dinh An M. N., Groenendaal L., Meijer E. W. (1997). Substituent Effects
on the Electrochemical Properties of Pyrroles and Small Oligopyrroles. Chem. Mater..

[142] Eickenscheidt M., Singler E., Stieglitz T. (2019). Pulsed electropolymerization
of PEDOT enabling controlled branching. Polym.
J..

[143] Heinze J., John H., Dietrich M., Tschuncky P. (2001). σ-“Dimers”
- key intermediates and products during generation and redox switching
of conjugated oligomers and polymers. Synth.
Met..

[144] Smie A., Synowczyk A., Heinze J., Alle R., Tschuncky P., Götz G., Bäuerle P. (1998). β,β-Disubstituted
oligothiophenes, a new oligomeric approach towards the synthesis of
conducting polymers. J. Electroanal. Chem..

[145] Correia J. P., Vieil E., Abrantes L. M. (2004). Electropolymerization
of 3-methylthiophene studied by multiflux convolution. J. Electroanal. Chem..

[146] Sidorov D. A., Pud A. A. (2010). In situ spectroelectrochemical
study
of dissolved oligomer products formed in the electrochemical polymerization
of 3-methylthiophene. Theor. Exp. Chem..

[147] Casado N., Hernández G., Veloso A., Devaraj S., Mecerreyes D., Armand M. (2016). PEDOT Radical Polymer with Synergetic
Redox and Electrical Properties. ACS Macro Lett..

[148] Jadamiec M., Lapkowski M., Matlengiewicz M., Brembilla A., Henry B., Rodehüser L. (2007). Electrochemical
and spectroelectrochemical evidence of dimerization and oligomerization
during the polymerization of terthiophenes. Electrochim. Acta.

[149] Deng H., Van Berkel G. J. (1999). Electrochemical
Polymerization of
Aniline Investigated Using On-Line Electrochemistry/Electrospray Mass
Spectrometry. Anal. Chem..

[150] Lang P., Chao F., Costa M., Garnier F. (1987). Electrochemical
grafting of poly­(methylthiophene) onto platinum in acetonitrile. Polymer.

[151] Wei Y., Chan C. C., Tian J., Jang G. W., Hsueh K. F. (1991). Electrochemical
polymerization of thiophenes in the presence of bithiophene or terthiophene:
kinetics and mechanism of the polymerization. Chem. Mater..

[152] Kenner J. (1968). Benzidine Rearrangement. Nature.

[153] Hand R. L., Nelson R. F. (1974). Anodic oxidation
pathways of N-alkylanilines. J. Am. Chem. Soc..

[154] Guay J., Kasai P., Diaz A., Wu R., Tour J. M., Dao L. H. (1992). Chain-length dependence of electrochemical
and electronic properties of neutral and oxidized soluble.alpha.,.alpha.-coupled
thiophene oligomers. Chem. Mater..

[155] Wei Y., Tian J., Glahn D., Wang B., Chu D. (1993). Kinetics and
activation parameters of electrochemical polymerization of 3-alkylthiophenes
in the presence of various aromatic additives. J. Phys. Chem..

[156] Wei Y., Jang G. W., Chan C. C., Hsueh K. F., Hariharan R., Patel S. A., Whitecar C. K. (1990). Polymerization
of aniline and alkyl
ring-substituted anilines in the presence of aromatic additives. J. Phys. Chem..

[157] Lin C.-W., Mak W. H., Chen D., Wang H., Aguilar S., Kaner R. B. (2019). Catalytic Effects
of Aniline Polymerization
Assisted by Oligomers. ACS Catal..

[158] Tan Y., Ghandi K. (2013). Kinetics and mechanism
of pyrrole chemical polymerization. Synth. Met..

[159] Li, Y. ; Yu, H. ; Zhang, Y. ; Zhou, N. ; Tan, Z. Kinetics and characterization of preparing conductive nanofibrous membrane by In-situ polymerization of Polypyrrole on electrospun nanofibers. Chemical Engineering Journal 2022, 433, 133531. 10.1016/j.cej.2021.133531

[160] Camarada M. B., Jaque P., Díaz F. R., del Valle M. A. (2011). Oxidation potential of thiophene oligomers: Theoretical
and experimental approach. J. Polym. Sci., Part
B: Polym. Phys..

[161] Cosnier, S. ; Karyakin, A. Electropolymerization; Wiley, 2010.

[162] Lin, S. X. ; Wu, Q. P. ; Lu, Y. Recent Progress of the Application of Electropolymerization in Batteries and Supercapacitors: Specific Design of Functions in Electrodes. Chemelectrochem 2024, 11 (12), 10.1002/celc.202300776.

[163] Rashti A., Moncada J., Zhang X., Carrero C. A., Oh T.-S. (2019). Thermally grown copper nanowire electrodes
modified by electropolymerization. Mater. Chem.
Phys..

[164] Otero T. F., Rodríguez J. (1991). Polythiophene electrogeneration on
a rotating disk electrode: The influence of water on polymerization
and polymer properties. Journal of Electroanalytical
Chemistry and Interfacial Electrochemistry.

[165] Town J. L., MacLaren F., Dewald H. D. (1991). Rotating
disk voltammetry
experiment. J. Chem. Educ..

[166] Lim K., Goines S., Deng M., McCormick H., Kauffmann P. J., Dick J. E. (2023). A troubleshooting
guide for laser
pulling platinum nanoelectrodes. Analyst.

[167] Liu Y., Li M., Zhang F., Zhu A., Shi G. (2015). Development
of Au Disk Nanoelectrode Down to 3 nm in Radius for Detection of Dopamine
Release from a Single Cell. Anal. Chem..

[168] Li Y., Bergman D., Zhang B. (2009). Preparation
and electrochemical response
of 1–3 nm Pt disk electrodes. Anal. Chem..

[169] Xue J. A., Xian Y. Z., Ying X. Y., Chen J. S., Wang L., Jin L. T. (2000). Fabrication of an
ultramicrosensor
for measurement of extracellular myocardial superoxide. Anal. Chim. Acta.

[170] Khani H., Wipf D. O. (2019). Fabrication of Tip-Protected
Polymer-Coated
Carbon-Fiber Ultramicroelectrodes and pH Ultramicroelectrodes. J. Electrochem. Soc..

[171] Welle T. M., Alanis K., Colombo M. L., Sweedler J. V., Shen M. (2018). A high spatiotemporal study of somatic
exocytosis with scanning electrochemical
microscopy and nanoITIES electrodes. Chem. Sci..

[172] Fletcher B. L., Fern J. T., Rhodes K., McKnight T. E., Fowlkes J. D., Retterer S. T., Keffer D. J., Simpson M. L., Doktycz M. J. (2009). Effects
of ultramicroelectrode dimensions on the electropolymerization
of polypyrrole. J. Appl. Phys..

[173] David M., Barsan M. M., Brett C. M. A., Florescu M. (2018). Improved glucose
label-free biosensor with layer-by-layer architecture and conducting
polymer poly­(3,4-ethylenedioxythiophene). Sens.
Actuators, B.

[174] Johnson B. J., Park S. M. (1996). Electrochemistry of Conductive Polymers:
XX. Early Stages of Aniline Polymerization Studied by Spectroelectrochemical
and Rotating Ring Disk Electrode Techniques. J. Electrochem. Soc..

[175] Dervisevic M., Dervisevic E., Azak H., Çevik E., Senel M., Yildiz H. B. (2016). Novel amperometric xanthine biosensor
based on xanthine oxidase immobilized on electrochemically polymerized
10­[4-dithieno­(3,2-:2′,3′-)­pyrrole-4-yl] decane-1-amine
film. Sens Actuators B Chem..

[176] Abdel-Aziz A. M., Hassan H. H., Badr I. H. A. (2020). Glassy
Carbon
Electrode Electromodification in the Presence of Organic Monomers:
Electropolymerization versus Activation. Anal.
Chem..

[177] Kong Y. T., Boopathi M., Shim Y. B. (2003). Direct electrochemistry
of horseradish peroxidase bonded on a conducting polymer modified
glassy carbon electrode. Biosens Bioelectron.

[178] Sharma, E. ; Rathi, R. ; Misharwal, J. ; Sinhmar, B. ; Kumari, S. ; Dalal, J. ; Kumar, A. Evolution in Lithography Techniques: Microlithography to Nanolithography. Nanomaterials (Basel) 2022, 12 (16), 2754. 10.3390/nano12162754 36014619 PMC9414268

[179] Snopok, B. ; Laroussi, A. ; Cafolla, C. ; Voïtchovsky, K. ; Snopok, T. ; Mirsky, V. M. Gold surface cleaning by etching polishing: Optimization of polycrystalline film topography and surface functionality for biosensing. Surfaces and Interfaces 2021, 22, 100818. 10.1016/j.surfin.2020.100818

[180] Lee J., Suh H. N., Park H. B., Park Y. M., Kim H. J., Kim S. (2023). Regenerative Strategy of Gold Electrodes
for Long-Term Reuse of Electrochemical
Biosensors. ACS Omega.

[181] Gonçalves D., Irene E. A. (2001). A study of gold-coated
glass as electrodes
for electropolymerization of 3-methylthiophene. Langmuir.

[182] Berkes B. B., Bandarenka A. S., Inzelt G. (2015). Electropolymerization:
Further Insight into the Formation of Conducting Polyindole Thin Films. J. Phys. Chem. C.

[183] Camalet J. L., Lacroix J. C., Nguyen T. D., Aeiyach S., Pham M. C., Petitjean J., Lacaze P. C. (2000). Aniline electropolymerization
on platinum and mild steel from neutral aqueous media. J. Electroanal. Chem..

[184] Ghazal M., Daher Mansour M., Scholaert C., Dargent T., Coffinier Y., Pecqueur S., Alibart F. (2022). Bio-Inspired
Adaptive Sensing through Electropolymerization of Organic Electrochemical
Transistors. Adv. Electron Mater..

[185] Sun H., Lu B. Y., Duan X. M., Xu J. K., Dong L. Q., Zhu X. F., Zhang K. X., Hu D. F., Ming S. L. (2015). Electrosynthesis
and Characterization of a New Conducting Copolymer from 2′-aminomethyl-3,4-ethylenedioxythiophene
and 3,4-ethylenedioxythiophene. Int. J. Electrochem.
Sci..

[186] Kim P., Epstein A. K., Khan M., Zarzar L. D., Lipomi D. J., Whitesides G. M., Aizenberg J. (2012). Structural
transformation by electrodeposition
on patterned substrates (STEPS): a new versatile nanofabrication method. Nano Lett..

[187] Hu G., Kang J., Ng L. W. T., Zhu X., Howe R. C. T., Jones C. G., Hersam M. C., Hasan T. (2018). Functional inks and
printing of two-dimensional materials. Chem.
Soc. Rev..

[188] Chang J. S., Facchetti A. F., Reuss R. (2017). A Circuits and Systems
Perspective of Organic/Printed Electronics: Review, Challenges, and
Contemporary and Emerging Design Approaches. Ieee Journal on Emerging and Selected Topics in Circuits and Systems.

[189] Park Y. G., Yun I., Chung W. G., Park W., Lee D. H., Park J. U. (2022). High-Resolution
3D Printing for Electronics. Adv. Sci. (Weinh).

[190] Silva B. V.M., Rodriguez B. A.G., Sales G. F., Sotomayor M. D. P. T., Dutra R. F. (2016). An ultrasensitive
human cardiac troponin T graphene
screen-printed electrode based on electropolymerized-molecularly imprinted
conducting polymer. Biosens Bioelectron.

[191] Swain, G. M. 5 - Solid Electrode Materials: Pretreatment and Activation. In Handbook of Electrochemistry; Zoski, C. G. , Ed.; Elsevier, 2007; pp 111–153.

[192] Berman D., Krim J. (2012). Impact of oxygen and
argon plasma
exposure on the roughness of gold film surfaces. Thin Solid Films.

[193] Cumpson P., Sano N. (2013). Stability of reference
masses V:
UV/ozone treatment of gold and platinum surfaces. Metrologia.

[194] Raiber K., Terfort A., Benndorf C., Krings N., Strehblow H.-H. (2005). Removal
of self-assembled monolayers of alkanethiolates
on gold by plasma cleaning. Surf. Sci..

[195] Cui X. T., Zhou D. D. (2007). Poly (3,4-ethylenedioxythiophene)
for chronic neural stimulation. IEEE Trans Neural
Syst. Rehabil Eng..

[196] Green R. A., Hassarati R. T., Bouchinet L., Lee C. S., Cheong G. L., Yu J. F., Dodds C. W., Suaning G. J., Poole-Warren L. A., Lovell N. H. (2012). Substrate dependent
stability of conducting polymer coatings on medical electrodes. Biomaterials.

[197] Láng G. G., Ujvári M., Bazsó F., Vesztergom S., Ujhelyi F. (2012). In situ monitoring
of the electrochemical
degradation of polymer films on metals using the bending beam method
and impedance spectroscopy. Electrochim. Acta.

[198] Pranti A. S., Schander A., Bödecker A., Lang W. (2018). PEDOT:PSS coating on gold microelectrodes with excellent stability
and high charge injection capacity for chronic neural interfaces. Sens. Actuators B Chem..

[199] Ganji M., Hossain L., Tanaka A., Thunemann M., Halgren E., Gilja V., Devor A., Dayeh S. A. (2018). Monolithic
and Scalable Au Nanorod Substrates Improve PEDOT-Metal Adhesion and
Stability in Neural Electrodes. Adv. Healthc
Mater..

[200] Boehler C., Oberueber F., Schlabach S., Stieglitz T., Asplund M. (2017). Long-Term Stable Adhesion for Conducting
Polymers in Biomedical Applications: IrOx and Nanostructured Platinum
Solve the Chronic Challenge. ACS Appl. Mater.
Interfaces.

[201] Mandal H. S., Knaack G. L., Charkhkar H., McHail D. G., Kastee J. S., Dumas T. C., Peixoto N., Rubinson J. F., Pancrazio J. J. (2014). Improving the performance of poly­(3,4-ethylenedioxythiophene)
for brain-machine interface applications. Acta
Biomater.

[202] Chhin D., Polcari D., Guen C. B.-L., Tomasello G., Cicoira F., Schougaard S. B. (2018). Diazonium-Based Anchoring of PEDOT
on Pt/Ir Electrodes via Diazonium Chemistry. J. Electrochem. Soc..

[203] Wei B., Liu J., Ouyang L., Kuo C. C., Martin D. C. (2015). Significant
enhancement of PEDOT thin film adhesion to inorganic solid substrates
with EDOT-acid. ACS Appl. Mater. Interfaces.

[204] Ouyang L., Wei B., Kuo C. C., Pathak S., Farrell B., Martin D. C. (2017). Enhanced
PEDOT adhesion on solid
substrates with electrografted P­(EDOT-NH(2)). Sci. Adv..

[205] Kim S., Jang L. K., Park H. S., Lee J. Y. (2016). Electrochemical
deposition of conductive and adhesive polypyrrole-dopamine films. Sci. Rep.

[206] Inoue A., Yuk H., Lu B., Zhao X. (2020). Strong adhesion
of wet conducting polymers on diverse substrates. Sci. Adv..

[207] Ferreira C. A., Aeiyach S., Aaron J. J., Lacaze P. C. (1996). Electrosynthesis
of strongly adherent polypyrrole coatings on iron and mild steel in
aqueous media. Electrochim. Acta.

[208] Nishizawa M., Miwa Y., Matsue T., Uchida I. (1993). Surface Pretreatment
for Electrochemical Fabrication of Ultrathin Patterned Conducting
Polymers. J. Electrochem. Soc..

[209] Watanabe T., Ohira M., Koizumi Y., Nishiyama H., Tomita I., Inagi S. (2018). In-Plane Growth
of Poly­(3,4-ethylenedioxythiophene)
Films on a Substrate Surface by Bipolar Electropolymerization. ACS Macro Lett..

[210] Zou F., Huang X. (2018). Electropolymerization
in proton-functionalized anilinium
salts/glycol deep eutectic solvents. J. Mater.
Sci..

[211] Zou F., Yu X., Zhang J., Cheng N., Huang X. (2015). Electropolymerization
in a novel proton functionalized room temperature ionic liquid anilinium
acetate. Synth. Met..

[212] Seki Y., Takahashi M., Takashiri M. (2019). Effects of
different electrolytes and film thicknesses on structural and thermoelectric
properties of electropolymerized poly­(3,4-ethylenedioxythiophene)
films. RSC Adv..

[213] Gvozdenović, M. M. ; Jugović, B. Z. ; Stevanović, J. S. ; Trišović, T. L. ; Grgur, B. N. Electrochemical Polymerization of Aniline. In Electropolymerization; Schab-Balcerzak, E. , Ed.; 2011; Chapter 4, pp 77–96.

[214] Damlin P., Kvarnström C., Ivaska A. (2004). Electrochemical synthesis
and in situ spectroelectrochemical characterization of poly­(3,4-ethylenedioxythiophene)
(PEDOT) in room temperature ionic liquids. J.
Electroanal. Chem..

[215] Wagner K., Pringle J. M., Hall S. B., Forsyth M., MacFarlane D. R., Officer D. L. (2005). Investigation of
the electropolymerisation
of EDOT in ionic liquids. Synth. Met..

[216] Lu W., Fadeev A. G., Qi B., Smela E., Mattes B. R., Ding J., Spinks G. M., Mazurkiewicz J., Zhou D., Wallace G. G. (2002). Use
of Ionic Liquids
for π-Conjugated Polymer Electrochemical Devices. Science.

[217] Ahmad S., Deepa M., Singh S. (2007). Electrochemical
Synthesis
and Surface Characterization of Poly­(3,4-ethylenedioxythiophene) Films
Grown in an Ionic Liquid. Langmuir.

[218] Pringle J.
M., Forsyth M., MacFarlane D. R., Wagner K., Hall S. B., Officer D. L. (2005). The influence
of
the monomer and the ionic liquid on the electrochemical preparation
of polythiophene. Polymer.

[219] Chiang T.-Y., Huang M.-C., Tsai C.-H. (2014). The effects
of solvent
on the electrochromic properties of poly­(3,4-ethylenedioxythiophene). RSC Adv..

[220] Bodart C., Rossetti N., Hagler J. E., Chevreau P., Chhin D., Soavi F., Schougaard S. B., Amzica F., Cicoira F. (2019). Electropolymerized Poly­(3,4-ethylenedioxythiophene)
(PEDOT) Coatings for Implantable Deep-Brain-Stimulating Microelectrodes. ACS Appl. Mater. Interfaces.

[221] Ming S., Feng Z., Mo D., Wang Z., Lin K., Lu B., Xu J. (2016). Solvent effects
on electrosynthesis,
morphological and electrochromic properties of a nitrogen analog of
PEDOT. Phys. Chem. Chem. Phys..

[222] Yadav P., Naqvi S., Patra A. (2020). Poly­(3,4-ethylenedioxyselenophene):
effect of solvent and electrolyte on electrodeposition, optoelectronic
and electrochromic properties. RSC Adv..

[223] Krukiewicz K., Jarosz T., Herman A. P., Turczyn R., Boncel S., Zak J. K. (2016). The effect of solvent on the synthesis
and physicochemical properties of poly­(3,4-ethylenedioxypyrrole). Synth. Met..

[224] Singhal S., Patra A. (2020). Benzothiadiazole bridged
EDOT based
donor-acceptor polymers with tunable optical, electrochemical, morphological
and electrochromic performance: effects of solvents and electrolytes. Phys. Chem. Chem. Phys..

[225] Zhu D., Mo D., Ma X., Zhou Q., Liu H., Xu J., Zhou W., Zhao F. (2016). Effect of polymerization solvent,
potential, and temperature on morphology and capacitance properties
of poly­(thieno­[3,2-b]­thiophene) films. Synth.
Met..

[226] Zhang Y., Lu B., Dong L., Sun H., Hu D., Xing H., Duan X., Chen S., Xu J. (2016). Solvent effects
on the synthesis, characterization and electrochromic properties of
acetic acid modified polyterthiophene. Electrochim.
Acta.

[227] Danielsson P., Bobacka J., Ivaska A. (2004). Electrochemical synthesis
and characterization of poly­(3,4-ethylenedioxythiophene) in ionic
liquids with bulky organic anions. J. Solid
State Electrochem..

[228] Chen Z., Zhou Y., Villani E., Shida N., Tomita I., Inagi S. (2023). AC-Bipolar Electropolymerization
of 3,4-Ethylenedioxythiophene in Ionic Liquids. Langmuir.

[229] Fernández R. A., Benedetti T. M., Torresi R. M. (2015). Comparative electrochemical
performance of electrodeposited polypyrrole in protic and aprotic
ionic liquids. J. Electroanal. Chem..

[230] Snook G. A., Greaves T. L., Best A. S. (2011). A comparative
study
of the electrodeposition of polyaniline from a protic ionic liquid,
an aprotic ionic liquid and neutral aqueous solution using anilinium
nitrate. J. Mater. Chem..

[231] Shen L., Huang X. (2018). Electrochemical polymerization
of
aniline in a protic ionic liquid with high proton activity. Synth. Met..

[232] Ji, J. ; Zhu, X. ; Han, D. ; Li, M. ; Zhang, Q. ; Shu, Y. ; Cheng, Z. ; Zhang, W. ; Hua, E. ; Sang, S. AC Electrodeposition of PEDOT Films in Protic Ionic Liquids for Long-Term Stable Organic Electrochemical Transistors. Molecules 2019, 24 (22), 4105. 10.3390/molecules24224105 31739407 PMC6891491

[233] Abdelhamid M. E., Snook G. A., O’Mullane A. P. (2015). Electropolymerisation
of Catalytically Active PEDOT from an Ionic Liquid on a Flexible Carbon
Cloth Using a Sandwich Cell Configuration. ChemPlusChem..

[234] Liu K., Hu Z., Xue R., Zhang J., Zhu J. (2008). Electropolymerization
of high stable poly­(3,4-ethylenedioxythiophene) in ionic liquids and
its potential applications in electrochemical capacitor. J. Power Sources.

[235] Sekiguchi K., Atobe M., Fuchigami T. (2003). Electrooxidative
polymerization of aromatic compounds in 1-ethyl-3-methylimidazolium
trifluoromethanesulfonate room-temperature ionic liquid. J. Electroanal. Chem..

[236] Lu W., Fadeev A. G., Qi B., Mattes B. R. (2004). Fabricating
Conducting
Polymer Electrochromic Devices Using Ionic Liquids. J. Electrochem. Soc..

[237] Nancarrow P., Al-Othman A., Mital D. K., Döpking S. (2021). Comprehensive
analysis and correlation of ionic liquid conductivity data for energy
applications. Energy.

[238] Innocenti M., Loglio F., Pigani L., Seeber R., Terzi F., Udisti R. (2005). In situ atomic force microscopy in
the study of electrogeneration of polybithiophene on Pt electrode. Electrochim. Acta.

[239] Liu Q., Wang Y., Zhang Y., Xu S., Wang J. (2012). Effect of
dopants on the adsorbing performance of polypyrrole/graphite electrodes
for capacitive deionization process. Synth.
Met..

[240] Páramo-García U., Ibanez J. G., Batina N. (2011). Electrochemical
Modulation of the Thickness of Polypyrrole Films by Using Different
Anionic Dopants. Int. J. Electrochem. Sci..

[241] Paramo-García U., Ibanez J. G., Batina N. (2013). AFM Analysis of Polypyrrole
Films Synthesized in the Presence of Selected Doping Agents. Int. J. Electrochem. Sci..

[242] Jakhar P., Shukla M., Singh V. (2019). Investigation
of dopant
effect on the electrochemical performance of 1-D polypyrrole nanofibers
based glucose biosensor. Journal of Materials
Science: Materials in Electronics.

[243] Krukiewicz K., Kruk A., Turczyn R. (2018). Evaluation
of drug
loading capacity and release characteristics of PEDOT/naproxen system:
Effect of doping ions. Electrochim. Acta.

[244] Srinives S., Sarkar T., Mulchandani A. (2013). Nanothin Polyaniline
Film for Highly Sensitive Chemiresistive Gas Sensing. Electroanalysis.

[245] Kemp N. T., Cochrane J. W., Newbury R. (2007). Patterning
of conducting
polymer nanowires on gold/platinum electrodes. Nanotechnology.

[246] Nishizawa M., Shibuya M., Sawaguchi T., Matsue T., Uchida I. (1991). Electrochemical preparation of ultrathin
polypyrrole film at microarray electrodes. J.
Phys. Chem..

[247] Kemp N. T., McGrouther D., Cochrane J. W., Newbury R. (2007). Bridging the
Gap: Polymer Nanowire Devices. Adv. Mater..

[248] Pothipor C., Lertvachirapaiboon C., Shinbo K., Kato K., Ounnunkad K., Baba A. (2020). Detection of creatinine using silver
nanoparticles on a poly­(pyrrole) thin film-based surface plasmon resonance
sensor. Jpn. J. Appl. Phys..

[249] Zhao W., Hanson L., Lou H.-Y., Akamatsu M., Chowdary P. D., Santoro F., Marks J. R., Grassart A., Drubin D. G., Cui Y., Cui B. (2017). Nanoscale
manipulation
of membrane curvature for probing endocytosis in live cells. Nat. Nanotechnol..

[250] Liu Y., McGuire A. F., Lou H.-Y., Li T. L., Tok J. B. H., Cui B., Bao Z. (2018). Soft conductive
micropillar electrode
arrays for biologically relevant electrophysiological recording. Proc. Natl. Acad. Sci. U. S. A..

[251] Wang B., Kong Y., Zhang S., Wu Z., Wang S., Ren J., Woo H. Y., Li Y., Ma W. (2024). Face-on Orientation Matches Vertical Organic Electrochemical Transistors
for High Transconductance and Superior Non-Volatility. Adv. Funct. Mater..

[252] Fukami K., Nakanishi S., Yamasaki H., Tada T., Sonoda K., Kamikawa N., Tsuji N., Sakaguchi H., Nakato Y. (2007). General Mechanism for the Synchronization of Electrochemical
Oscillations and Self-Organized Dendrite Electrodeposition of Metals
with Ordered 2D and 3D Microstructures. J. Phys.
Chem. C.

[253] Marken, F. ; Neudeck, A. ; Bond, A. M. Cyclic Voltammetry. In Electroanalytical Methods: Guide to Experiments and Applications; Scholz, F. ; Bond, A. M. ; Compton, R. G. ; Fiedler, D. A. ; Inzelt, G. ; Kahlert, H. , Komorsky-Lovrić, Š. ; Lohse, H. ; Lovrić, M. ; Marken, F. , et al., Eds.; Springer Berlin Heidelberg, 2010; pp 57–106.

[254] Uppalapati D., Boyd B. J., Garg S., Travas-Sejdic J., Svirskis D. (2016). Conducting polymers with defined micro- or nanostructures
for drug delivery. Biomaterials.

[255] Pan L., Qiu H., Dou C., Li Y., Pu L., Xu J., Shi Y. (2010). Conducting polymer
nanostructures: template synthesis
and applications in energy storage. International
journal of molecular sciences.

[256] Chen Z., Villani E., Inagi S. (2021). Recent progress
in
bipolar electropolymerization methods toward one-dimensional conducting
polymer structures. Current Opinion in Electrochemistry.

[257] Diouf K., Dramé A., Diouf A., Orange F., Guittard F., Perepichka I. F., Darmanin T. (2023). Directional formation
of microtubes by soft-template electropolymerization from fully conjugated
triphenylamine-based monomers. J. Electroanal.
Chem..

[258] Wang W., You S., Gong X., Qi D., Chandran B. K., Bi L., Cui F., Chen X. (2016). Bioinspired
Nanosucker Array for Enhancing Bioelectricity Generation in Microbial
Fuel Cells. Adv. Mater..

[259] Li C., Bai H., Shi G. (2009). Conducting
polymer nanomaterials:
electrosynthesis and applications. Chem. Soc.
Rev..

[260] Wakita J.-i., Ràfols I., Itoh H., Matsuyama T., Matsushita M. (1998). Experimental Investigation on the Formation of Dense-Branching-Morphology-Like
Colonies in Bacteria. J. Phys. Soc. Jpn..

[261] Iber D., Menshykau D. (2013). The control of branching morphogenesis. Open Biology.

[262] Meakin, P. ; Fowler, A. D. Diffusion-limited Aggregation in the Earth Sciences. In Fractals in Petroleum Geology and Earth Processes; Barton, C. C. , La Pointe, P. R. , Eds.; Springer US, 1995; pp 227–261.

[263] Lupo C., Schlettwein D. (2019). Modeling of
Dendrite Formation as
a Consequence of Diffusion-Limited Electrodeposition. J. Electrochem. Soc..

[264] Bozzini B., Lacitignola D., Mele C., Sgura I. (2012). Coupling of
Morphology and Chemistry Leads to Morphogenesis in Electrochemical
Metal Growth: A Review of the Reaction-Diffusion Approach. Acta Applicandae Mathematicae.

[265] Dhara T., Ghosh U. U., Ghosh A., Vishnugopi B. S., Mukherjee P. P., DasGupta S. (2022). Mechanistic Underpinnings
of Morphology
Transition in Electrodeposition under the Application of Pulsatile
Potential. Langmuir.

[266] Witten T. A., Sander L. M. (1983). Diffusion-limited
aggregation. Phys. Rev. B.

[267] Witten T. A., Sander L. M. (1981). Diffusion-Limited
Aggregation, a
Kinetic Critical Phenomenon. Phys. Rev. Lett..

[268] Mu S., Chen C., Wang J. (1997). The kinetic behavior for the electrochemical
polymerization of aniline in aqueous solution. Synth. Met..

[269] Kaufman J. H., Melroy O. R., Abraham F. F., Nazzal A. I. (1986). Growth
instability in diffusion controlled polymerization. Solid State Commun..

[270] Fujii M., Arii K., Yoshino K. (1990). Electrochemical
growth
of poly­(3-dodecylthiophene) and its interpretation as a fractal. J. Phys.: Condens. Matter.

[271] Kaufman J. H., Nazzal A. I., Melroy O. R., Kapitulnik A. (1987). Onset of fractal
growth: Statics and dynamics of diffusion-controlled polymerization. Phys. Rev. B.

[272] Shi Y., Luo S.-C., Fang W., Zhang K., Ali E. M., Boey F. Y. C., Ying J. Y., Wang J., Yu H.-h., Li L.-J. (2008). Work function engineering
of electrodes via electropolymerization
of ethylenedioxythiophenes and its derivatives. Org. Electron..

[273] Ciccone G., Cucchi M., Gao Y., Kumar A., Seifert L. M., Weissbach A., Tseng H., Kleemann H., Alibart F., Leo K. (2022). Growth and design strategies of organic
dendritic networks. Discover Materials.

[274] Barisci J. N., Stella R., Spinks G. M., Wallace G. G. (2000). Characterisation
of the topography and surface potential of electrodeposited conducting
polymer films using atomic force and electric force microscopies. Electrochim. Acta.

[275] Kumar A., Janzakova K., Coffinier Y., Pecqueur S., Alibart F. (2022). Theoretical modeling
of dendrite
growth from conductive wire electro-polymerization. Sci. Rep..

[276] Zwanzig R., Harrison A. K. (1985). Modifications of the Stokes-Einstein
formula. J. Chem. Phys..

[277] Anderson J. L., Rauh F., Morales A. (1978). Particle diffusion
as a function of concentration and ionic strength. J. Phys. Chem..

[278] Paul, A. ; Laurila, T. ; Vuorinen, V. ; Divinski, S. V. Fick’s Laws of Diffusion. In Thermodynamics, Diffusion and the Kirkendall Effect in Solids; Paul, A. , Laurila, T. , Vuorinen, V. , Divinski, S. V. , Eds.; Springer International Publishing, 2014; pp 115–139.

[279] Guo J., Lindner E. (2009). Cyclic Voltammograms at Coplanar and Shallow Recessed
Microdisk Electrode Arrays: Guidelines for Design and Experiment. Anal. Chem..

[280] Shida N., Inagi S. (2020). Bipolar electrochemistry in synergy
with electrophoresis: electric field-driven electrosynthesis of anisotropic
polymeric materials. Chem. Commun..

[281] Muñoz E., Colina Á., Heras A., Ruiz V., Palmero S., López-Palacios J. (2006). Electropolymerization
and characterization of polyaniline films using a spectroelectrochemical
flow cell. Anal. Chim. Acta.

[282] Lin Y., Wallace G. G. (1994). Electropolymerisation
of pyrrole under hydrodynamic
conditions—effect of solution additives. Electrochim. Acta.

[283] Ren X., Pickup P. G. (2001). Simulation and analysis
of the impedance behaviour
of electroactive layers with non-uniform conductivity and capacitance
profiles. Electrochim. Acta.

[284] Leventis N., Dass A., Chandrasekaran N. (2007). Mass transfer
effects on the electropolymerization current efficiency of 3-methylthiophene
in the magnetic field. J. Solid State Electrochem..

[285] Ramos Chagas G., Darmanin T., Guittard F. (2016). One-Step and Templateless
Electropolymerization Process Using Thienothiophene Derivatives To
Develop Arrays of Nanotubes and Tree-like Structures with High Water
Adhesion. ACS Appl. Mater. Interfaces.

[286] Bai S., Hu Q., Zeng Q., Wang M., Wang L. (2018). Variations
in Surface Morphologies, Properties, and Electrochemical Responses
to Nitro-Analyte by Controlled Electropolymerization of Thiophene
Derivatives. ACS Appl. Mater. Interfaces.

[287] Najafisayar P., Bahrololoom M. E. (2013). The effect of pulse electropolymerization
on the electrochemical properties of polythiophene films. Electrochim. Acta.

[288] Peng X.-Y., Luan F., Liu X.-X., Diamond D., Lau K.-T. (2009). pH-controlled morphological structure
of polyaniline
during electrochemical deposition. Electrochim.
Acta.

[289] Stejskal J., Sapurina I., Trchová M. (2010). Polyaniline
nanostructures and the role of aniline oligomers in their formation. Prog. Polym. Sci..

[290] Das A., Lei C. H., Elliott M., Macdonald J. E., Turner M. L. (2006). Non-lithographic fabrication of PEDOT
nano-wires between
fixed Au electrodes. Org. Electron..

[291] Musumeci C., Hutchison J. A., Samorì P. (2013). Controlling
the morphology of conductive PEDOT by in situ electropolymerization:
from thin films to nanowires with variable electrical properties. Nanoscale.

[292] Ohira M., Koizumi Y., Nishiyama H., Tomita I., Inagi S. (2017). Synthesis of linear PEDOT fibers
by AC-bipolar electropolymerization in a micro-space. Polym. J..

[293] Klein M., Waldvogel S. R. (2022). Counter Electrode Reactions-Important
Stumbling Blocks on the Way to a Working Electro-organic Synthesis. Angew. Chem., Int. Ed. Engl..

[294] Vij V., Sultan S., Harzandi A. M., Meena A., Tiwari J. N., Lee W.-G., Yoon T., Kim K. S. (2017). Nickel-Based Electrocatalysts
for Energy-Related Applications: Oxygen Reduction, Oxygen Evolution,
and Hydrogen Evolution Reactions. ACS Catal..

[295] Huo, L. ; Jin, C. ; Jiang, K. ; Bao, Q. ; Hu, Z. ; Chu, J. Applications of Nickel-Based Electrocatalysts for Hydrogen Evolution Reaction. Advanced Energy and Sustainability Research 2022, 3 (4), 10.1002/aesr.202100189.

[296] Song F., Li W., Han G., Sun Y. (2018). Electropolymerization
of Aniline on Nickel-Based Electrocatalysts Substantially Enhances
Their Performance for Hydrogen Evolution. ACS
Applied Energy Materials.

[297] Park H., Vecitis C. D., Hoffmann M. R. (2008). Solar-powered
electrochemical
oxidation of organic compounds coupled with the cathodic production
of molecular hydrogen. J. Phys. Chem. A.

[298] Koizumi Y., Ohira M., Watanabe T., Nishiyama H., Tomita I., Inagi S. (2018). Synthesis of Poly­(3,4-ethylenedioxythiophene)-Platinum
and Poly­(3,4-ethylenedioxythiophene)-Poly­(styrenesulfonate) Hybrid
Fibers by Alternating Current Bipolar Electropolymerization. Langmuir.

[299] del Olmo D., Pavelka M., Kosek J. (2021). Open-Circuit
Voltage
Comes from Non-Equilibrium Thermodynamics. Journal
of Non-Equilibrium Thermodynamics.

[300] Berman J. M., Awayda M. S. (2013). Redox artifacts
in electrophysiological
recordings. Am. J. Physiol Cell Physiol.

[301] Matarrese, R. ; Mascia, M. ; Vacca, A. ; Mais, L. ; Usai, E. M. ; Ghidelli, M. ; Mascaretti, L. ; Bricchi, B. R. ; Russo, V. ; Casari, C. S. Integrated Au/TiO2 Nanostructured Photoanodes for Photoelectrochemical Organics Degradation. Catalysts 2019, 9 (4), 340. 10.3390/catal9040340

[302] Bard, A. J. ; Faulkner, L. R. ; White, H. S. Electrochemical methods: fundamentals and applications; John Wiley & Sons, 2000.

[303] Priyadarshini D., Li C., Rilemark R., Abrahamsson T., Donahue M. J., Strakosas X., Ek F., Olsson R., Musumeci C., Fabiano S. (2025). Tuning
the Organic Electrochemical
Transistor (OECT) Threshold Voltage with Monomer Blends. Adv. Electron Mater..

[304] Zhou Y., Shida N., Koizumi Y., Endo K., Tomita I., Inagi S. (2020). Fabrication of One-Dimensional Polymer
Nanowires by Templated Bipolar Electropolymerization Promoted by Electrophoretic
Effect. Macromolecules.

[305] Chen H., Anderson J. L., Anand R. K. (2022). Electropolymerization
of Pyrrole-Based Ionic Liquids on Selected Wireless Bipolar Electrodes. ACS Appl. Mater. Interfaces.

[306] Kong S., Fontaine O., Roche J., Bouffier L., Kuhn A., Zigah D. (2014). Electropolymerization
of Polypyrrole
by Bipolar Electrochemistry in an Ionic Liquid. Langmuir.

[307] Lee J., Yoon T. W., Chung S., Cho K., Zhang G., Kang B. (2024). Remote-Controllable Lateral Electropolymerization of Conducting Polymers
via Dual-Electrode ADC-Bipolar Electrochemistry. Adv. Funct. Mater..

[308] Fosdick S. E., Knust K. N., Scida K., Crooks R. M. (2013). Bipolar
Electrochemistry. Angew. Chem., Int. Ed..

[309] Bouffier L., Sojic N., Kuhn A. (2017). Capillary-assisted
bipolar electrochemistry: A focused mini review. ELECTROPHORESIS.

[310] Shida N., Zhou Y., Inagi S. (2019). Bipolar Electrochemistry:
A Powerful Tool for Electrifying Functional Material Synthesis. Acc. Chem. Res..

[311] Karimian N., Hashemi P., Afkhami A., Bagheri H. (2019). The principles
of bipolar electrochemistry and its electroanalysis applications. Current Opinion in Electrochemistry.

[312] Loget G., Lapeyre V., Garrigue P., Warakulwit C., Limtrakul J., Delville M.-H., Kuhn A. (2011). Versatile
Procedure
for Synthesis of Janus-Type Carbon Tubes. Chem.
Mater..

[313] Villani E., Inagi S. (2024). Electrosynthesis with split-bipolar
electrodes. Current Opinion in Electrochemistry.

[314] Alkire R. (1973). A Theoretical Study of Bipolar Porous
Electrodes. J. Electrochem. Soc..

[315] Fleischmann M., Ghoroghchian J., Rolison D., Pons S. (1986). Electrochemical
behavior of dispersions of spherical ultramicroelectrodes. J. Phys. Chem..

[316] Goodridge F., King C., Wright A. (1977). Performance
studies
on a bipolar fluidised bed electrode. Electrochim.
Acta.

[317] Oloman C., Watkinson A. P. (1979). Hydrogen peroxide production in trickle-bed
electrochemical reactors. J. Appl. Electrochem..

[318] Mavré F., Anand R. K., Laws D. R., Chow K.-F., Chang B.-Y., Crooks J. A., Crooks R. M. (2010). Bipolar Electrodes:
A Useful Tool for Concentration, Separation, and Detection of Analytes
in Microelectrochemical Systems. Anal. Chem..

[319] Babu S., Ndungu P., Bradley J.-C., Rossi M. P., Gogotsi Y. (2005). Guiding water into carbon nanopipes
with the aid of
bipolar electrochemistry. Microfluid. Nanofluid..

[320] Chassagne P., Garrigue P., Kuhn A. (2024). Bulk Electrosynthesis
of Patchy Particles with Highly Controlled Asymmetric Features. Adv. Mater..

[321] Yan Z.-B., Huang F.-F., Shi J.-Q., Li F., Li B., Guo Z.-G., Xie J.-H., He J.-B. (2022). Bipolar electrodeposition
of gradient polypyrrole films as a catalyst matrix for anodic ethanol
oxidation. Mater. Chem. Phys..

[322] Dumitrescu I., Anand R. K., Fosdick S. E., Crooks R. M. (2011). Pressure-Driven
Bipolar Electrochemistry. J. Am. Chem. Soc..

[323] Iwai S., Suzuki T., Sakagami H., Miyamoto K., Chen Z., Konishi M., Villani E., Shida N., Tomita I., Inagi S. (2022). Electropolymerization
without an
electric power supply. Communications Chemistry.

[324] Gschwend G. C., Girault H. H. (2020). Discrete Helmholtz
charge distribution
at liquid-liquid interfaces: Electrocapillarity, capacitance and non-linear
spectroscopy studies. J. Electroanal. Chem..

[325] Gschwend G. C., Olaya A., Peljo P., Girault H. H. (2020). Structure
and reactivity of the polarised liquid-liquid interface: what we know
and what we do not. Current Opinion in Electrochemistry.

[326] Gorgy K., Fusalba F., Evans U., Kontturi K., Cunnane V. J. (2001). Electropolymerization of 2,2′:5′,2″
terthiophene at an electrified liquid-liquid interface. Synth. Met..

[327] Vignali M., Edwards R. A. H., Serantoni M., Cunnane V. J. (2006). Electropolymerized polythiophene layer extracted from
the interface between two immiscible electrolyte solutions: Current-time
analysis. J. Electroanal. Chem..

[328] Lehane R. A., Gamero-Quijano A., Malijauskaite S., Holzinger A., Conroy M., Laffir F., Kumar A., Bangert U., McGourty K., Scanlon M. D. (2022). Electrosynthesis
of Biocompatible Free-Standing PEDOT Thin Films at a Polarized Liquid|Liquid
Interface. J. Am. Chem. Soc..

[329] Zhou Z., He D. L., Guo Y. N., Cui Z. D., Wang M. H., Li G. X., Yang R. H. (2009). Fabrication
of polyaniline-silver
nanocomposites by chronopotentiometry in different ionic liquid microemulsion
systems. Thin Solid Films.

[330] Moshrefi, R. ; Connors, E. P. ; Merschrod, E. ; Stockmann, T. J. Simultaneous electropolymerization/Au nanoparticle generation at an electrified liquid/liquid micro-interface. Electrochim. Acta 2022, 426, 140749. 10.1016/j.electacta.2022.140749

[331] Moshrefi R., Ryan K., Connors E. P., Walsh J. C., Merschrod E., Bodwell G. J., Stockmann T. J. (2023). Electrosynthesis
of Au nanocluster embedded conductive polymer films at soft interfaces
using dithiafulvenyl-functionalized pyrene. Nanoscale.

[332] Jetmore H. D., Anupriya E. S., Cress T. J., Shen M. (2022). Interface
between Two Immiscible Electrolyte Solutions Electrodes for Chemical
Analysis. Anal. Chem..

[333] Bard A. J., Zoski C. G. (2000). Voltammetry Retrospective. Anal. Chem..

[334] Prejza J., Lundström I., Skotheim T. (1982). Electropolymerization
of Pyrrole in the Presence of Fluoborate. J.
Electrochem. Soc..

[335] Kissinger P. T., Heineman W. R. (1983). Cyclic voltammetry. J. Chem.
Educ..

[336] Elgrishi N., Rountree K. J., McCarthy B. D., Rountree E. S., Eisenhart T. T., Dempsey J. L. (2018). A Practical Beginner’s Guide
to Cyclic Voltammetry. J. Chem. Educ..

[337] Batchelor-McAuley C. (2023). Defining the
onset potential. Current Opinion in Electrochemistry.

[338] Agarwal, R. Dos and Dont’s in Determination of Electrochemical Kinetic Parameters of Reversible and Irreversible Redox couples using Cyclic Voltammetry. J. Chem. Educ. 2025, 102, 66. 10.1021/acs.jchemed.4c01224 40580121

[339] Et Taouil A., Contal E., Lakard S., Lakard B. (2021). Investigation
of electrochemical oxidative coupling of 3 and 6 substituted carbazoles. J. Electroanal. Chem..

[340] Heinze J., Rasche A., Pagels M., Geschke B. (2007). On the Origin
of the So-Called Nucleation Loop during Electropolymerization of Conducting
Polymers. J. Phys. Chem. B.

[341] Oehzelt M., Koch N., Heimel G. (2014). Organic semiconductor
density of states controls the energy level alignment at electrode
interfaces. Nature Comm.

[342] Tybrandt, K. ; Zozoulenko, I. V. ; Berggren, M. Chemical potential-electric double layer coupling in conjugated polymer-polyelectrolyte blends. Sci. Adv. 2017, 3 (12), 10.1126/sciadv.aao3659. PMC5734606 29260000

[343] Volkov, A. V. ; Wijeratne, K. ; Mitraka, E. ; Ail, U. ; Zhao, D. ; Tybrandt, K. ; Andreasen, J. W. ; Berggren, M. ; Crispin, X. ; Zozoulenko, I. V. Understanding the Capacitance of PEDOT:PSS. Adv. Funct. Mater. 2017, 27 (28), 10.1002/adfm.201700329.

[344] Costentin C., Savéant J.-M. (2019). Energy
storage: pseudocapacitance
in prospect. Chem. Sci..

[345] Proctor C. M., Rivnay J., Malliaras G. G. (2016). Understanding
volumetric capacitance in conducting polymers. J. Polym. Sci., Part B: Polym. Phys..

[346] Hillman A. R., Daisley S. J., Bruckenstein S. (2007). Kinetics and
mechanism of the electrochemical p-doping of PEDOT. Electrochem. Commun..

[347] Liu T., Finn L., Yu M., Wang H., Zhai T., Lu X., Tong Y., Li Y. (2014). Polyaniline and Polypyrrole Pseudocapacitor
Electrodes with Excellent Cycling Stability. Nano Lett..

[348] Salinas G., Frontana-Uribe B. A. (2019). Analysis of Conjugated Polymers Conductivity
by in situ Electrochemical-Conductance Method. ChemElectroChem..

[349] Kittlesen G. P., White H. S., Wrighton M. S. (1984). Chemical
derivatization
of microelectrode arrays by oxidation of pyrrole and N-methylpyrrole:
fabrication of molecule-based electronic devices. J. Am. Chem. Soc..

[350] Genies E. M., Hany P., Lapkowski M., Santier C., Olmedo L. (1988). In situ conductivity and photoconductivity
measurements of polyaniline films. Synth. Met..

[351] Paul E. W., Ricco A. J., Wrighton M. S. (1985). Resistance of polyaniline
films as a function of electrochemical potential and the fabrication
of polyaniline-based microelectronic devices. J. Phys. Chem..

[352] Thackeray J. W., White H. S., Wrighton M. S. (1985). Poly­(3-methylthiophene)-coated
electrodes: optical and electrical properties as a function of redox
potential and amplification of electrical and chemical signals using
poly­(3-methylthiophene)-based microelectrochemical transistors. J. Phys. Chem..

[353] Kankare J., Kupila E.-L. (1992). In-situ conductance
measurement during
electropolymerization. J. Electroanal. Chem..

[354] Schiavon G., Sitran S., Zotti G. (1989). A simple two-band electrode
for in situ conductivity measurements of polyconjugated conducting
polymers. Synth. Met..

[355] Gerasimov J. Y., Tu D., Hitaishi V., Harikesh P. C., Yang C.-Y., Abrahamsson T., Rad M., Donahue M. J., Ejneby M. S., Berggren M. (2023). A Biologically
Interfaced
Evolvable Organic Pattern Classifier. Adv. Sci..

[356] Easley A. D., Ma T., Eneh C. I., Yun J., Thakur R. M., Lutkenhaus J. L. (2022). A practical guide to quartz crystal
microbalance with dissipation monitoring of thin polymer films. J. Polym. Sci..

[357] Kousseff C. J., Wustoni S., Silva R. K. S., Lifer A., Savva A., Frey G. L., Inal S., Nielsen C. B. (2024). Single-Component
Electroactive Polymer Architectures for Non-Enzymatic Glucose Sensing. Adv. Sci..

[358] Palma-Cando, A. ; Rendón-Enríquez, I. ; Tausch, M. ; Scherf, U. Thin Functional Polymer Films by Electropolymerization. In Nanomaterials, 2019, 9, 1125. 10.3390/nano9081125 31382661 PMC6723103

[359] Luo J., Liu M., Zhao Q., Zhao J., Zhang Y., Tan L., Tang H., Xie Q., Li H., Yao S. (2010). A study on
the electro-oxidation and electropolymerization of a new OPE linear
molecule by EQCM and in situ FTIR spectroelectrochemistry. Electrochim. Acta.

[360] Pigani L., Heras A., Colina Á., Seeber R., López-Palacios J. (2004). Electropolymerisation
of 3,4-ethylenedioxythiophene
in aqueous solutions. Electrochem. Commun..

[361] Krikstolaityte V., Ding R., Ruzgas T., Björklund S., Lisak G. (2020). Characterization of nano-layered solid-contact ion selective electrodes
by simultaneous potentiometry and quartz crystal microbalance with
dissipation. Anal. Chim. Acta.

[362] Bandey H. L., Robert Hillman A., Brown M. J., Martin S. J. (1997). Viscoelastic
characterization of electroactive polymer films at the electrode/solution
interface. Faraday Discuss..

[363] Donavan K. C., Arter J. A., Weiss G. A., Penner R. M. (2012). Virus-Poly­(3,4-ethylenedioxythiophene)
Biocomposite Films. Langmuir.

[364] Zhao, M. ; Tang, X. ; Zhang, H. ; Gu, C. ; Ma, Y. Characterization of complicated electropolymerization using UV-vis spectroelectrochemistry and an electrochemical quartz-crystal microbalance with dissipation: A case study of tricarbazole derivatives. Electrochem. Commun. 2021, 123, 106913. 10.1016/j.elecom.2020.106913

[365] Gribkova, O. L. ; Nekrasov, A. A. Spectroelectrochemistry of Electroactive Polymer Composite Materials Polymers (Basel) 2022, 14 (15), 3201. 10.3390/polym14153201 35956715 PMC9370871

[366] Furukawa Y. (1996). Electronic Absorption and Vibrational Spectroscopies
of Conjugated Conducting Polymers. J. Phys.
Chem..

[367] Malinauskas A., Holze R. (1999). An in situ UV—vis spectroelectrochemical
investigation of the initial stages in the electrooxidation of selected
ring- and nitrogen-alkylsubstituted anilines. Electrochim. Acta.

[368] Zhang G., Zhang A., Liu X., Zhao S., Zhang J., Lu J. (2010). Investigation of the
electropolymerization
of o-toluidine and p-phenylenediamine and their electrocopolymerization
by in situ ultraviolet-visible spectroelectrochemistry. J. Appl. Polym. Sci..

[369] Genies E. M., Lapkowski M. (1987). Spectroelectrochemical
evidence for
an intermediate in the electropolymerization of aniline. Journal of Electroanalytical Chemistry and Interfacial Electrochemistry.

[370] Furukawa, Y. Vibrational Spectroscopy of Conducting Polymers: Fundamentals and Applications. In Handbook of Vibrational Spectroscopy, 2001. 10.1002/9780470027325.s6107.pub2.

[371] Trchová, M. ; Stejskal, J. Polyaniline: The infrared spectroscopy of conducting polymer nanotubes (IUPAC Technical Report). 2011, 83 (10), 1803–1817. 10.1351/PAC-REP-10-02-01

[372] Kvarnström C., Neugebauer H., Blomquist S., Ahonen H. J., Kankare J., Ivaska A. (1999). In situ spectroelectrochemical
characterization of poly­(3,4-ethylenedioxythiophene). Electrochim. Acta.

[373] Yang Q., Zhang Y., Li H., Zhang Y., Liu M., Luo J., Tan L., Tang H., Yao S. (2010). Electrochemical
copolymerization study of o-toluidine and o-aminophenol by the simultaneous
EQCM and in situ FTIR spectroelectrochemisty. Talanta.

[374] Langer J., Jimenez de Aberasturi D., Aizpurua J., Alvarez-Puebla R. A., Auguié B., Baumberg J. J., Bazan G. C., Bell S. E. J., Boisen A., Brolo A. G. (2020). Present
and Future of Surface-Enhanced Raman Scattering. ACS Nano.

[375] Mažeikienė, R. ; Niaura, G. ; Malinauskas, A. In situ time-resolved Raman spectroelectrochemical study of aniline polymerization at platinum and gold electrodes. Chemija 2018, 29 (2), 10.6001/chemija.v29i2.3710.

[376] Olk C. H., Beetz C. P., Heremans J. (1988). Raman spectra
during
the electropolymerization of polypyrrole. J.
Mater. Res..

[377] Saçak M., Akbulut U., Batchelder D. N. (1999). Monitoring
of electroinitiated polymerization of aniline by Raman microprobe
spectroscopy. Polymer.

[378] Bandeira M. C. E., Crayston J. A., Gonçalves N. S., Noda L. K., Glidle A., Franco C. V. (2006). Electropolymerization
of trans-[RuCl2­(vpy)­4] complex—EQCM and Raman studies. J. Solid State Electrochem..

[379] Kim Y. T., Allara D. L., Collins R. W., Vedam K. (1990). Real-time
spectroscopic ellipsometry study of theelectrochemical deposition
of polypyrrole thin films. Thin Solid Films.

[380] Correia J. P., Graczyk M., Abrantes L. M., Vorotyntsev M. A. (2007). Polypyrrole
films functionalized with pendant titanocene dichloride complexes:
Ellipsometric study of the electropolymerization process. Electrochim. Acta.

[381] Monnin A. F., Buron C. C., Guyard L., Filiâtre C. (2012). In situ investigations
of electrogenerated polybithiophene film growth on indium tin oxide
substrate using optical fixed-angle reflectometry. Thin Solid Films.

[382] Chabot V., Miron Y., Grandbois M., Charette P. G. (2012). Long range surface plasmon resonance for increased
sensitivity in living cell biosensing through greater probing depth. Sens. Actuators, B.

[383] Jing J.-Y., Wang Q., Zhao W.-M., Wang B.-T. (2019). Long-range
surface plasmon resonance and its sensing applications: A review. Optics and Lasers in Engineering.

[384] Balciunas D., Plausinaitis D., Ratautaite V., Ramanaviciene A., Ramanavicius A. (2022). Towards electrochemical
surface plasmon
resonance sensor based on the molecularly imprinted polypyrrole for
glyphosate sensing. Talanta.

[385] Reggente M., Passeri D., Rossi M., Tamburri E., Terranova M. L. (2017). Electrochemical atomic force microscopy: In situ monitoring
of electrochemical processes. AIP Conf. Proc..

[386] Baba A., Knoll W., Advincula R. (2006). Simultaneous
in situ electrochemical, surface plasmon optical, and atomic force
microscopy measurements: Investigation of conjugated polymer electropolymerization. Rev. Sci. Instrum..

[387] Chen C., Peng X.-P., Yau S. (2022). The adsorption and
electropolymerization of terthiophene on Au(111) electrode - Probed
by in situ STM. J. Electroanal. Chem..

[388] Lapitan L. D. S., Tongol B. J. V., Yau S.-L. (2010). Molecular
Assembly and Electropolymerization of 3,4-Ethylenedioxythiophene on
Au(111) Single Crystal Electrode as Probed by In Situ Electrochemical
STM in 0.10 M HClO4. Langmuir.

[389] Chen S., Hwuang C., Tu H., Wu C., Yau S., Fan L., Yang Y. (2010). In situ STM study of
the adsorption
and electropolymerization of o-, m-, and p-ethylaniline molecules
on Au(111) electrode. Phys. Chem. Chem. Phys..

[390] Rivnay J., Wang H., Fenno L., Deisseroth K., Malliaras G. G. (2017). Next-generation probes, particles,
and proteins for
neural interfacing. Sci. Adv..

[391] Cogan S. F. (2008). Neural Stimulation and Recording
Electrodes. Annu. Rev. Biomed. Eng..

[392] Boehler C., Carli S., Fadiga L., Stieglitz T., Asplund M. (2020). Tutorial: guidelines for standardized
performance tests
for electrodes intended for neural interfaces and bioelectronics. Nat. Protoc..

[393] Nikiforidis G., Wustoni S., Routier C., Hama A., Koklu A., Saleh A., Steiner N., Druet V., Fiumelli H., Inal S. (2020). Benchmarking the Performance
of Electropolymerized
Poly­(3,4-ethylenedioxythiophene) Electrodes for Neural Interfacing. Macromol. Biosci..

[394] Cui X., Lee V. A., Raphael Y., Wiler J. A., Hetke J. F., Anderson D. J., Martin D. C. (2001). Surface modification of neural recording
electrodes with conducting polymer/biomolecule blends. J. Biomed. Mater. Res..

[395] Green R. A., Hassarati R. T., Goding J. A., Baek S., Lovell N. H., Martens P. J., Poole-Warren L. A. (2012). Conductive
Hydrogels: Mechanically Robust Hybrids for Use as Biomaterials. Macromol. Biosci..

[396] Jones P. D., Moskalyuk A., Barthold C., Gutöhrlein K., Heusel G., Schröppel B., Samba R., Giugliano M. (2020). Low-Impedance
3D PEDOT:PSS Ultramicroelectrodes. Frontiers
in Neuroscience.

[397] Schmidt C. E., Shastri V. R., Vacanti J. P., Langer R. (1997). Stimulation
of neurite outgrowth using an electrically conducting polymer. Proc. Natl. Acad. Sci. U. S. A..

[398] Ludwig K. A., Uram J. D., Yang J., Martin D. C., Kipke D. R. (2006). Chronic neural recordings using silicon
microelectrode
arrays electrochemically deposited with a poly­(3,4-ethylenedioxythiophene)
(PEDOT) film. Journal of Neural Engineering.

[399] Cui X., Martin D. C. (2003). Electrochemical deposition and characterization of
poly­(3,4-ethylenedioxythiophene) on neural microelectrode arrays. Sens. Actuators, B.

[400] Green, R. A. ; Lim, K. S. ; Henderson, W. C. ; Hassarati, R. T. ; Martens, P. J. ; Lovell, N. H. ; Poole-Warren, L. A. Living electrodes: Tissue engineering the neural interface. In 2013 35th Annual International Conference of the IEEE Engineering in Medicine and Biology Society (EMBC), 2013/07//, 2013; IEEE: Osaka, pp 6957–6960. 10.1109/EMBC.2013.6611158 24111345

[401] Green R. A., Matteucci P. B., Hassarati R. T., Giraud B., Dodds C. W. D., Chen S., Byrnes-Preston P. J., Suaning G. J., Poole-Warren L. A., Lovell N. H. (2013). Performance of conducting
polymer electrodes for stimulating neuroprosthetics. Journal of Neural Engineering.

[402] Chik G. K. K., Xiao N., Ji X., Tsang A. C. O., Leung G. K. K., Zhang S., Tin C., Chan P. K. L. (2022). Flexible
Multichannel Neural Probe Developed by Electropolymerization for Localized
Stimulation and Sensing. Advanced Materials
Technologies.

[403] Yang J., Martin D. C. (2004). Microporous conducting polymers on
neural microelectrode arrays. Sens. Actuators,
B.

[404] Yang J., Kim D. H., Hendricks J. L., Leach M., Northey R., Martin D. C. (2005). Ordered surfactant-templated
poly­(3,4-ethylenedioxythiophene) (PEDOT) conducting polymer on microfabricated
neural probes. Acta Biomaterialia.

[405] Abidian M. R., Kim D. H., Martin D. C. (2006). Conducting-Polymer
Nanotubes for Controlled Drug Release. Adv.
Mater..

[406] Pham J., Forget A., Bridonneau N., Mattana G., Stavrinidou E., Zrig S., Piro B., Noel V. (2022). In vivo electrochemically-assisted polymerization of conjugated functionalized
terthiophenes inside the vascular system of a plant. Electrochem. Commun..

[407] Lai J., Yi Y., Zhu P., Shen J., Wu K., Zhang L., Liu J. (2016). Polyaniline-based glucose biosensor:
A review. J. Electroanal. Chem..

[408] Wallace, G. G. ; Adeloju, S. B. ; Shaw, S. J. Electroassembly of smart polymer structures (role of polyelectrolytes). 1997/2/14/, SPIE: 1997; Vol. 3040, pp 160–167.

[409] Nicolini T., Shinde S., El-Attar R., Salinas G., Thuau D., Abbas M., Raoux M., Lang J., Cloutet E., Kuhn A. (2024). Fine-Tuning the Optoelectronic
and
Redox Properties of an Electropolymerized Thiophene Derivative for
Highly Selective OECT-Based Zinc Detection. Advanced Materials Interfaces.

[410] Mariani F., Gualandi I., Tessarolo M., Fraboni B., Scavetta E. (2018). PEDOT: Dye-Based, Flexible Organic
Electrochemical Transistor for High ly Sensitive pH Monitoring. ACS Appl. Mater. Interfaces.

[411] Wustoni S., Combe C., Ohayon D., Akhtar M. H., McCulloch I., Inal S. (2019). Membrane-Free Detection
of Metal
Cations with an Organic Electrochemic al Transistor. Adv. Funct. Mater..

[412] Salinas G., Villarroel Marquez A., Idir M., Shinde S., Frontana-Uribe B. A., Raoux M., Lang J., Cloutet E., Kuhn A. (2020). Sodium-Ion Selectivity Study of a Crown-Ether-Functionalized PEDOT
Ana log. ChemElectroChem..

[413] Villarroel Marquez A., Salinas G., Abarkan M., Idir M., Brochon C., Hadziioannou G., Raoux M., Kuhn A., Lang J., Cloutet E. (2020). Design of
Potassium-Selective Mixed
Ion/Electron Conducting Polymers. Macromol.
Rapid Commun..

[414] Yun M., Myung N. V., Vasquez R. P., Lee C., Menke E., Penner R. M. (2004). Electrochemically Grown Wires for Individually Addressable
Sensor Arrays. Nano Lett..

[415] Ramanathan K., Bangar M. A., Yun M., Chen W., Mulchandani A., Myung N. V. (2004). Individually Addressable
Conducting
Polymer Nanowires Array. Nano Lett..

[416] Sulka G. D., Hnida K., Brzózka A. (2013). pH sensors
based on polypyrrole nanowire arrays. Electrochim.
Acta.

[417] Demuru S., Kunnel B. P., Briand D. (2021). Thin film organic electrochemical
transistors based on hybrid PANI/PED. Biosensors
and Bioelectronics: X.

[418] Nowacki M., Wałȩsa-Chorab M. (2023). Influence of temperature
on electrochemical and electrochromic properties of naphthalenediimide-triphenylamine-based
polymer. Prog. Org. Coat..

[419] Satyanarayana M., Yugender Goud K., Koteshwara Reddy K., Vengatajalabathy Gobi K. (2017). Conducting Polymer-Layered
Carbon
Nanotube as Sensor Interface for Electrochemical Detection of Dacarbazine
In-Vitro. Electrocatalysis.

[420] Farshadinia A., Kolahdoozan M. (2019). A new porous
copolymer electrocatalyst:
the optimal synthesis, characterization, and application for the measurement
of amoxicillin. J. Appl. Electrochem..

[421] Castagnola E., Robbins E. M., Krahe D. D., Wu B., Pwint M. Y., Cao Q., Cui X. T. (2023). Stable *in-vivo* electrochemical sensing
of tonic serotonin level s using PEDOT/CNT-coated
glassy carbon flexible microelectrode arrays. Biosens. Bioelectron..

[422] Vidal J.-C., Garcia-Ruiz E., Castillo J.-R. (2003). Recent Advances
in Electropolymerized Conducting Polymers in Amperomet ric Biosensors. Microchimica Acta.

[423] Lyu X., Gonzalez R., Horton A., Li T. (2021). Immobilization of Enzymes
by Polymeric Materials. Catalysts.

[424] Foulds N. C., Lowe C. R. (1986). Enzyme entrapment
in electrically
conducting polymers. Immobilisation of glucose oxidase in polypyrrole
and its application in amperometric glucose sensors. Journal of the Chemical Society, Faraday Transactions 1: Physical
Chem. istry in Condensed Phases.

[425] Umana M., Waller J. (1986). Protein-modified electrodes. The
glucose oxidase/polypyrrole system. Anal. Chem..

[426] Bartlett P. N., Whitaker R. G. (1987). Strategies for the development of
amperometric enzyme electrodes. Biosensors.

[427] Cosnier S. (2003). Biosensors
based on electropolymerized films: new trends. Anal. Bioanal. Chem..

[428] Ghorbani Zamani F., Moulahoum H., Ak M., Odaci Demirkol D., Timur S. (2019). Current trends in the
development of conducting polymers-based biosens
ors. TrAC Trends in Analytical Chemistry.

[429] Shinohara H., Chiba T., Aizawa M. (1988). Enzyme microsensor
for glucose with an electrochemically synthesized e nzyme-polyaniline
film. Sens. Actuators.

[430] Yang G., Kampstra K. L., Abidian M. R. (2014). High-Performance
Conducting Polymer Nanofiber Biosensors for Detection of Biomolecules. Advanced materials (Deerfield Beach, Fla.).

[431] Chen J., Zheng X., Li Y., Zheng H., Liu Y., Suye S.-i. (2020). A Glucose Biosensor
Based on Direct Electron Transfer
of Glucose Oxidase on PEDOT Modified Microelectrode. J. Electrochem. Soc..

[432] Kuwahara T., Oshima K., Shimomura M., Miyauchi S. (2005). Immobilization of glucose oxidase and electron-mediating
groups on the film of 3-methylthiophene/thiophene-3-acetic acid copolymer
and its a pplication to reagentless sensing of glucose. Polymer.

[433] Invernale M. A., Tang B. C., York R. L., Le L., Hou D. Y., Anderson D. G. (2014). Microneedle Electrodes Toward an
Amperometric Glucose-Sensing Smart Pa tch. Adv.
Healthc Mater..

[434] Patil D., Gaikwad A. B., Patil P. (2007). Poly­(o-anisidine) films
on mild steel: electrochemical synthesis and b iosensor application. J. Phys. D: Appl. Phys..

[435] Singh S., Solanki P. R., Pandey M. K., Malhotra B. D. (2006). Covalent
immobilization of cholesterol esterase and cholesterol oxidas e on
polyaniline films for application to cholesterol biosensor. Anal. Chim. Acta.

[436] Emre F. B., Ekiz F., Balan A., Emre S., Timur S., Toppare L. (2011). Conducting polymers
with benzothiadiazole
and benzoselenadiazole units for biosensor applications. Sens. Actuators, B.

[437] Tamer U., Seçkin A. İ., Temur E., Torul H. (2011). Fabrication
of Biosensor Based on Polyaniline/Gold Nanorod Composite. International Journal of Electrochemistry.

[438] Tan B., Baycan F. (2024). A novel PEDOT-derived
electroactive polymer-based enzymatic
glucose biosensor enriched with gold nanoparticles. Polym. Bull..

[439] Trettnak W., Lionti I., Mascini M. (1993). Cholesterol
biosensors
prepared by electropolymerization of pyrrole. Electroanalysis.

[440] Rahman M. A., Kwon N.-H., Won M.-S., Choe E. S., Shim Y.-B. (2005). Functionalized Conducting Polymer
as an Enzyme-Immobilizing
Substrate: An Amperometric Glutamate Microbiosensor for in Vivo Measurements. Anal. Chem..

[441] Vidal J. C., Garcıa E., Castillo J. R. (1999). In situ preparation
of a cholesterol biosensor: entrapment of choleste rol oxidase in
an overoxidized polypyrrole film electrodeposited in a flow system:
Determination of total cholesterol in serum. Anal. Chim. Acta.

[442] Rahman M. A., Park D.-S., Shim Y.-B. (2004). A performance comparison
of choline biosensors: anodic or cathodic det ections of H2O2 generated
by enzyme immobilized on a conducting polyme r. Biosens. Bioelectron..

[443] Braik M., Barsan M. M., Dridi C., Ben Ali M., Brett C. M. A. (2016). Highly sensitive amperometric enzyme
biosensor for
detection of supero xide based on conducting polymer/CNT modified
electrodes and superoxid e dismutase. Sens.
Actuators, B.

[444] Nguyen B. H., Nguyen B. T., Van Vu H., Van Nguyen C., Nguyen D. T., Nguyen L. T., Vu T. T., Tran L. D. (2016). Development
of label-free electrochemical lactose biosensor based on g raphene/poly­(1,5-diaminonaphthalene)
film. Curr. Appl. Phys..

[445] Ho K.-C., Yeh W.-M., Tung T.-S., Liao J.-Y. (2005). Amperometric
detection of morphine based on poly­(3,4-ethylenedioxythio phene) immobilized
molecularly imprinted polymer particles prepared by precipitation
polymerization. Anal. Chim. Acta.

[446] Wustoni S., Hidalgo T. C., Hama A., Ohayon D., Savva A., Wei N., Wehbe N., Inal S. (2020). In Situ Electrochemical
Synthesis of a Conducting Polymer Composite for Multimetabolite Sensing. Advanced Materials Technologies.

[447] Fenoy G. E., Hasler R., Quartinello F., Marmisollé W. A., Lorenz C., Azzaroni O., Bäuerle P., Knoll W. (2022). “Clickable” Organic Electrochemical Transistors. JACS Au.

[448] Alexander S., Baraneedharan P., Balasubrahmanyan S., Ramaprabhu S. (2017). Highly sensitive and selective non
enzymatic electrochemical
glucose s ensors based on Graphene Oxide-Molecular Imprinted Polymer. Materials Science and Engineering: C.

[449] Mohamad Nor N., Ridhuan N. S., Abdul
Razak K. (2022). Progress of
Enzymatic and Non-Enzymatic Electrochemical Glucose Biosen sor Based
on Nanomaterial-Modified Electrode. Biosensors.

[450] Liu Y., Liu T., Jiang D. (2023). Non-enzymatic
electrochemical sensor
for wearable monitoring of sweat biomarkers: A mini-review. Current Research in Biotechnology.

[451] Amiri M., Arshi S. (2020). An Overview on Electrochemical Determination
of Cholesterol. Electroanalysis.

[452] Rakesh Kumar R. K., Shaikh M. O., Chuang C.-H. (2021). A review of recent
advances in non-enzymatic electrochemical creatinin e biosensing. Anal. Chim. Acta.

[453] Majdinasab M., Daneshi M., Louis Marty J. (2021). Recent developments
in non-enzymatic (bio)­sensors for detection of pes ticide residues:
Focusing on antibody, aptamer and molecularly imprint ed polymer. Talanta.

[454] Bazaco R. B., Gómez R., Seoane C., Bäuerle P., Segura J. L. (2009). Specific recognition of a nucleobase-functionalized
poly­(3,4-ethylened ioxithiophene) (PEDOT) in aqueous media. Tetrahedron Lett..

[455] Cui M., Song Z., Wu Y., Guo B., Fan X., Luo X. (2016). A highly sensitive biosensor for
tumor maker alpha fetoprotein based
o n poly­(ethylene glycol) doped conducting polymer PEDOT. Biosens. Bioelectron..

[456] Zidarič T., Majer D., Maver T., Finšgar M., Maver U. (2023). The development of an electropolymerized,
molecularly imprinted polyme
r (MIP) sensor for insulin determination using single-drop analysis. Analyst.

[457] Wang N., Yang A., Fu Y., Li Y., Yan F. (2019). Functionalized Organic Thin Film Transistors for Biosensing. Acc. Chem. Res..

[458] Strakosas X., Sessolo M., Hama A., Rivnay J., Stavrinidou E. G., Malliaras G. M., Owens R. (2014). A facile biofunctionalisation
route for solution processable conductin g polymer devices. J. Mater. Chem. B.

[459] Gryszel M., Byun D., Burtscher B., Abrahamsson T., Brodsky J., Theodore Simon D., Berggren M., Daniel Glowacki E., Strakosas X., Jocelyn Donahue M. (2024). Vertical organic electrochemical transistor platforms
for efficient el ectropolymerization of thiophene based oligomers. Journal of Materials Chemistry C.

[460] Fenoy G. E., Marmisollé W.
A., Knoll W., Azzaroni O. (2022). Highly sensitive urine glucose detection with graphene
field-effect transistors functionalized with electropolymerized nanofilms. Sensors & Diagnostics.

[461] Fenoy G. E., Marmisollé W.
A., Azzaroni O., Knoll W. (2020). Acetylcholine biosensor based on the electrochemical functionalization
of graphene field-effect transistors. Biosens.
Bioelectron..

[462] Hai W., Goda T., Takeuchi H., Yamaoka S., Horiguchi Y., Matsumoto A., Miyahara Y. (2018). Human influenza virus detection using
sialyllactose-functionalized org anic electrochemical transistors. Sens. Actuators, B.

[463] Aerathupalathu Janardhanan J., Chen Y. L., Liu C. T., Tseng H. S., Wu P. I., She J. W., Hsiao Y. S., Yu H. H. (2022). Sensitive Detection of Sweat Cortisol Using an Organic Electrochemical
Transistor Featuring Nanostructured Poly­(3,4-Ethylenedioxythiophene)
Derivatives in the Channel Layer. Anal. Chem..

[464] Tao Y., Wang Y., Zhu R., Chen Y., Liu X., Li M., Yang L., Wang Y., Wang D. (2022). Fiber based organic
electrochemical transistor integrated with molecul arly imprinted
membrane for uric acid detection. Talanta.

[465] Tang K., Turner C., Case L., Mehrehjedy A., He X., Miao W., Guo S. (2022). Organic Electrochemical
Transistor
with Molecularly Imprinted Polymer- Modified Gate for the Real-Time
Selective Detection of Dopamine. ACS Applied
Polymer Materials.

[466] Lee Y., Park H.-L., Kim Y., Lee T.-W. (2021). Organic electronic
synapses with low energy consumption. Joule.

[467] van de Burgt Y., Lubberman E., Fuller E. J., Keene S. T., Faria G. C., Agarwal S., Marinella M. J., Talin A. A., Salleo A. (2017). A non-volatile
organic electrochemical
device as a low-voltage artificial synapse for neuromorphic computing. Nat. Mater..

[468] Fuller E. J., Keene S. T., Melianas A., Wang Z., Agarwal S., Li Y., Tuchman Y., James C. D., Marinella M. J., Yang J. J. (2019). Parallel programming
of an ionic floating-gate memory array for scalable neuromorphic computing. Science.

[469] Wang S., Chen X., Zhao C., Kong Y., Lin B., Wu Y., Bi Z., Xuan Z., Li T., Li Y. (2023). An organic electrochemical
transistor for multi-modal sensing, memory
and processing. Nature Electronics.

[470] Flagg L. Q., Bischak C. G., Onorato J. W., Rashid R. B., Luscombe C. K., Ginger D. S. (2019). Polymer Crystallinity
Controls Water
Uptake in Glycol Side-Chain Polymer Organic Electrochemical Transistors. J. Am. Chem. Soc..

[471] Gkoupidenis, P. ; Schaefer, N. ; Strakosas, X. ; Fairfield, J. A. ; Malliaras, G. G. Synaptic plasticity functions in an organic electrochemical transistor. Appl. Phys. Lett. 2015, 107, 10.1063/1.4938553.

[472] Hagiwara N., Sekizaki S., Kuwahara Y., Asai T., Akai-Kasaya M. (2021). Long- and
Short-Term Conductance Control of Artificial
Polymer Wire Synapses. Polymers (Basel).

[473] Ghazal M., Daher Mansour M., Scholaert C., Dargent T., Coffinier Y., Pecqueur S., Alibart F. (2022). Bio-Inspired
Adaptive Sensing through Electropolymerization of Organic Electrochemical
Transistors. Adv. Electron Mater..

[474] Mariani F., Decataldo F., Bonafè F., Tessarolo M., Cramer T., Gualandi I., Fraboni B., Scavetta E. (2024). High-Endurance
Long-Term Potentiation in Neuromorphic
Organic Electrochemical Transistors by PEDOT:PSS Electrochemical Polymerization
on the Gate Electrode. ACS Appl. Mater. Interfaces.

[475] Kandel, E. R. ; Schwartz, J. H. ; Jessell, T. M. ; Siegelbaum, S. ; Hudspeth, A. J. ; Mack, S. Principles of neural science; McGraw-hill New York, 2000.

[476] Gerasimov J. Y., Zhao D., Sultana A., Abrahamsson T., Han S., Bliman D., Tu D., Simon D. T., Olsson R., Crispin X. (2021). A Biomimetic
Evolvable Organic Electrochemical Transistor. Adv. Electron Mater..

[477] Curtis C. L., Ritchie J. E., Sailor M. J. (1993). Fabrication of Conducting
Polymer Interconnects. Science.

[478] Fujii M., Arii K., Yoshino K. (1995). Neuron-type polypyrrole
device prepared by electrochemical polymerization method and its properties. Synth. Met..

[479] Janzakova K., Ghazal M., Kumar A., Coffinier Y., Pecqueur S., Alibart F. (2021). Dendritic Organic Electrochemical
Transistors Grown by Electropolymerization for 3D Neuromorphic Engineering. Adv. Sci..

[480] Akai-Kasaya M., Hagiwara N., Hikita W., Okada M., Sugito Y., Kuwahara Y., Asai T. (2020). Evolving conductive
polymer neural networks on wetware. Jpn. J.
Appl. Phys..

[481] Janzakova K., Kumar A., Ghazal M., Susloparova A., Coffinier Y., Alibart F., Pecqueur S. (2021). Analog programing of
conducting-polymer dendritic interconnections and control of their
morphology. Nat. Commun..

[482] Cucchi M., Kleemann H., Tseng H., Ciccone G., Lee A., Pohl D., Leo K. (2021). Directed Growth
of Dendritic Polymer
Networks for Organic Electrochemical Transistors and Artificial Synapses. Adv. Electron Mater..

[483] Scholaert C., Janzakova K., Coffinier Y., Alibart F., Pecqueur S. (2022). Plasticity of conducting polymer
dendrites to bursts of voltage spikes in phosphate buffered saline. Neuromorphic Computing and Engineering.

[484] Janzakova K., Balafrej I., Kumar A., Garg N., Scholaert C., Rouat J., Drouin D., Coffinier Y., Pecqueur S., Alibart F. (2023). Structural plasticity
for neuromorphic
networks with electropolymerized dendritic PEDOT connections. Nat. Commun..

[485] Cucchi M., Gruener C., Petrauskas L., Steiner P., Tseng H., Fischer A., Penkovsky B., Matthus C., Birkholz P., Kleemann H., Leo K. (2021). Reservoir
computing with biocompatible organic electrochemical networks for
brain-inspired biosignal classification. Sci.
Adv..

[486] Cucchi M., Abreu S., Ciccone G., Brunner D., Kleemann H. (2022). Hands-on reservoir computing: a tutorial for practical
implementation. Neuromorphic Computing and Engineering.

[487] Ghazal M., Kumar A., Garg N., Pecqueur S., Alibart F. (2024). Neuromorphic Signal Classification
Using Organic Electrochemical
Transistor Array and Spiking Neural Simulations. IEEE Sensors Journal.

[488] Petrauskas L., Cucchi M., Grüner C., Ellinger F., Leo K., Matthus C., Kleemann H. (2022). Nonlinear
Behavior of Dendritic Polymer Networks for Reservoir Computing. Adv. Electron Mater..

[489] Hagiwara N., Asai T., Ando K., Akai-Kasaya M. (2023). Fabrication
and Training of 3D Conductive Polymer Networks for Neuromorphic Wetware. Adv. Funct. Mater..

